# Quantification of Interactions between Dynamic Cellular Network Functionalities by Cascaded Layering

**DOI:** 10.1371/journal.pcbi.1004235

**Published:** 2015-05-01

**Authors:** Thomas P. Prescott, Moritz Lang, Antonis Papachristodoulou

**Affiliations:** 1 Department of Engineering Science, University of Oxford, Oxford, United Kingdom; 2 Life Sciences Interface Doctoral Training Centre, University of Oxford, Oxford, United Kingdom; 3 Department of Biosystems Science and Engineering, ETH Zürich, Basel, Switzerland; 4 Swiss Institute of Bioinformatics, Basel, Switzerland; University of Tokyo, JAPAN

## Abstract

Large, naturally evolved biomolecular networks typically fulfil multiple functions. When modelling or redesigning such systems, functional subsystems are often analysed independently first, before subsequent integration into larger-scale computational models. In the design and analysis process, it is therefore important to quantitatively analyse and predict the dynamics of the interactions between integrated subsystems; in particular, how the incremental effect of integrating a subsystem into a network depends on the existing dynamics of that network. In this paper we present a framework for simulating the contribution of any given functional subsystem when integrated together with one or more other subsystems. This is achieved through a cascaded layering of a network into functional subsystems, where each layer is defined by an appropriate subset of the reactions. We exploit symmetries in our formulation to exhaustively quantify each subsystem’s incremental effects with minimal computational effort. When combining subsystems, their isolated behaviour may be amplified, attenuated, or be subject to more complicated effects. We propose the concept of mutual dynamics to quantify such nonlinear phenomena, thereby defining the incompatibility and cooperativity between all pairs of subsystems when integrated into any larger network. We exemplify our theoretical framework by analysing diverse behaviours in three dynamic models of signalling and metabolic pathways: the effect of crosstalk mechanisms on the dynamics of parallel signal transduction pathways; reciprocal side-effects between several integral feedback mechanisms and the subsystems they stabilise; and consequences of nonlinear interactions between elementary flux modes in glycolysis for metabolic engineering strategies. Our analysis shows that it is not sufficient to just specify subsystems and analyse their pairwise interactions; the environment in which the interaction takes place must also be explicitly defined. Our framework provides a natural representation of nonlinear interaction phenomena, and will therefore be an important tool for modelling large-scale evolved or synthetic biomolecular networks.

## Author Summary

To better understand the dynamic behaviour of cells and their interaction with the environment, mathematical models describing the interplay between proteins, metabolites or signalling molecules are used extensively in Systems Biology. Typically, such models focus on single functional subsystems and neglect the rest of the biochemical reaction network. However, the behaviour of multiple functional subsystems when integrated together can differ significantly from each subsystem’s isolated behaviour. In this article we describe a methodology for assessing the nonlinear effects of combining multiple functional subsystems of a biological system. This is key for answering questions related to Systems and Synthetic Biology as well as Metabolic Engineering. For example, if we can identify the isolated behaviours of two subsystems, we can determine if they persist when the subsystems interact. Similarly, we can show how modifications to single functional subsystems (such as increasing particular metabolic yields) have different effects in the context of the integrated system.

This is a *PLOS Computational Biology* Methods paper.

## Introduction

Complex biochemical reaction networks serve a broad variety of different tasks within the cell. Systems Biology researchers apply a range of systems analysis techniques to these networks to identify and model functional subsystems and their interaction structure. In the context of biomolecular networks, the subsystems that can be identified often have biological interpretations: for example, the heat shock response and the chemotaxis pathways represent two functional subsystems within a model describing the complete biomolecular reaction network of *Escherichia coli*; the synthesis pathways of individual products represent distinct functional subsystems within a metabolic network; and so on. Other functional subsystems may also have system-theoretic interpretations: for example, interacting, distributed feedback control mechanisms; or subsystems which can sense, compute, or actuate on the cell and its environment. This tangle of different objectives within the same network leads to trade-off situations: evolutionary or synthetic changes to one functional subsystem can lead to declining performance or unexpected side effects with respect to another.

A fundamental challenge of Systems Biology is to not only establish the behaviour of each functional subsystem in isolation, but also to understand how they dynamically influence one another. This problem is particularly acute when applying the modelling and analysis tools of Systems Biology to adapt and redesign modular biomolecular networks in Synthetic Biology [1–3]. The dynamics of many functional subsystems, whether evolved biochemical networks or synthetic devices, often do not proceed as modelled when integrated into a cell due to their interactions with one another and the environment of their cellular host. Possible sources of nonlinear interactions between pairs of functional subsystems and the cellular environment include retroactivity in genetic [4–6] and signalling [7] networks, crosstalk between parallel signalling pathways [8, 9], and the coupling of multiple transcription or translation rates through competition for shared resources [10–12]. In each of these settings, the change in input–output behaviour of a given subsystem upon integration with its context is examined. There are two complementary goals of this paper. First, we investigate the behaviour of each subsystem between the two extremes of ‘isolated’ or ‘integrated’, when it is integrated with any subset of the other subsystems. The second goal, which is achieved as a consequence of the first, is to then systematically quantify each of the pairwise interactions between the network’s subsystems.

**Fig 1:**
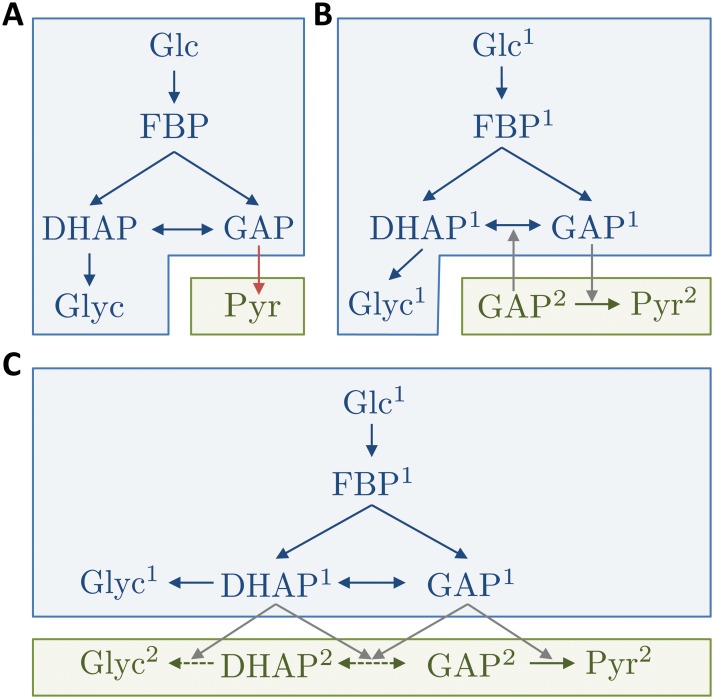
Modularization, non-cascaded and cascaded layering of a simplified model of the glycolytic pathway (see [46] p.88 ff.). Nodes represent metabolites, coloured arrows reactions, and grey arrows information transfer. Intermediate metabolites and cofactors are omitted, and several enzymatic reactions are merged. Modules (A) or layers (B and C) are delineated by blue and green boxes. In each panel the blue subnetwork corresponds to the single functional subsystem Glc→Glyc of glycolysis, where the conversion from GAP into Pyr has been removed. All panels consider the integration of the Glc→Glyc pathway (blue arrows) with the missing (merged) reaction GAP→Pyr. A) Modularization of the pathway [18–26]. Modules are typically defined by non-overlapping sets of species, and can be interconnected by mass flow (red arrow, representing the load of the new species) and information transfer. Here, there is mass flow when the blue module is integrated with the green module, leading to retroactive effects [4]. The combined dynamics will be in feedback, and hence both modules must be considered simultaneously. B) Non-cascaded layering [16, 17]. Layers are defined by non-overlapping sets of reactions, while species may take part in multiple layers; species’ affiliation to layers is indicated in superscript. The dynamics of species in multiple layers are summed (e.g. GAP(*t*) = GAP^1^(*t*) + GAP^2^(*t*) + GAP(*t* = 0)). Layers are interconnected only by information transfer (grey arrows) and their models are not modified when combined. However, as in the modular case, the layers are in feedback and must be considered simultaneously. C) Cascaded layering of the pathway, as introduced in this article. By allowing the layers to overlap in ‘altered reactions’ (green broken arrows) as well as species, the layers become cascaded as information (grey arrows) is only transferred in one direction. The states of the green layer directly capture the incremental effect of extending the isolated Glc→Glyc pathway with the additional (merged) reaction GAP→Pyr. Since the information transfer between the layers is cascaded, they can be numerically integrated and analysed sequentially.

The approach we will take in this work is to define a *functionality* as a group of reactions which corresponds to an identified functional subsystem of a biomolecular network. The reactions that determine each functionality can be selected either through biological insight, or by applying existing computational approaches such as elementary flux mode (EFM) analysis [13–15] (see also Results). We characterise the behaviour, or effect, of a functionality as the solution of an ordinary differential equation (ODE) model determined by the particular group of reactions. This approach exploits the recently-introduced decomposition technique known as *layering* [16, 17]. As depicted in Fig 1, such an approach is distinct from established modular approaches to network decomposition, which are characterised by identifying sets of species with a high connectivity inside the module, and significantly lower connectivity to species in other modules [18–26]. While often many species and reactions in a given network are implicated in multiple network functions, these modular approaches generally do not allow for such a high degree of overlap between modules. For example, if a network of two pathways responds to two external signals with a single output species, a modular decomposition of this network requires the common output species to be assigned to a module representing exactly one of the pathways, or potentially to an additional separate module. Either way, the input–output behaviour of both pathways cannot be easily defined. However, in the layered framework, the common output is associated with both layers, and hence the output of each layer can be defined in terms of its biological function. Thus, in some cases, layers are preferable to modules for defining the functional subsystems of the network, since the layered framework explicitly allows for overlap in species and reaction subsets [16, 17], as will be illustrated further in ‘Mapping Functionalities to Layers’ below.

Most importantly, we make it explicit that the behaviour of any functionality also depends on the other functionalities with which it is integrated, to which we refer as the context of the functionality. This contextual dependence is formalised by developing a notational framework that will unambiguously define a functionality’s behaviour in a particular context. The resulting concept of *conditional dynamics* will be key to our understanding of each functionality as being defined only in the context of others, allowing us to systematically investigate the interdependence of an entire network’s functionalities.

The subsequent aim of this framework is to characterise all of the interactions between each pair of functionalities, each of which is also context-dependent. Our approach is a formalisation and extension of previous investigations into additive (i.e. independent), synergistic, or antagonistic subsystem interactions. Examples of these phenomena include the cytokine secretion by macrophages in response to stimulation with different sets of ligands [27], the response of bacteria to different combinations of drugs [28], or calcium signalling responses to different stimuli [29]. Importantly, we demonstrate how the strength and the type of interactions between functionalities depends on mediated indirect interactions with the other functionalities comprising their context. The relationship of our approach to the concepts in [27–29] is further discussed in the section “Calculating With Layers”.

In addition to the previous literature on context-dependent dynamics, our theoretical framework is also related to steady-state methods. For instance, the third of our examples will exploit EFMs, a technique designed to analyse the steady-state flux distribution in metabolic networks [13–15]. Furthermore, modular and hierarchical control/response analysis is concerned with the different behaviour of subsystems both in isolation and integrated in larger systems, with particular reference to the steady-state responses of biochemical networks to parameter perturbations [6, 30–34]. The key distinction between our method and these is that we analyse the dynamics of kinetic models [35, 36] represented by sets of ODEs, rather than steady states. Moreover, our method is not based on linearisation, allowing us to adequately capture nonlinear interactions between functionalities. Quantifying the dynamic interactions of each pair of functionalities in all possible contexts requires multiple ODE simulations; for its practical applicability, it is important to minimise the computational effort involved.

This paper is structured as follows: in the Methods section we show how to use a layered decomposition to identify the incremental effect of a functionality, making its context-dependence explicit. We continue by defining the interdependence, or *mutual dynamics*, between any two functionalities. We summarise this interdependence by the incompatibility and the cooperativity between functionalities. In the final part of the Methods section we describe how to analyse all functionalities and their dependencies with minimal computation. We demonstrate our method on three familiar biomolecular networks in the Results section. The first example is of two signalling pathways with two crosstalk mechanisms, in which we use our approach to quantify the nonlinear interactions between crosstalk mechanisms. In the second example we analyse an unstable pathway stabilised by two integral feedback loops, finding the interactions between each controller and the pathway, and also between the controllers. Finally, we consider the glycolytic pathway in *Saccharomyces cerevisiae*, with functionalities defined by an EFM analysis. We apply our approach to compare how different knock-out strategies in metabolic engineering influence the yield of ethanol, industrially relevant in biofuel production.

## Methods

### Network Representation and Layering

Consider a biochemical reaction network with *N*_*X*_ species *X*_*i*_ of time-varying concentrations *x*_*i*_(*t*), taking part in *N*_*R*_ reactions *R*_1_, …, *R*_*N*_*R*__, each of which proceeds at the concentration-dependent rate *v*_*j*_(*x*_1_, …, *x*_*N*_*X*__) for *j* = 1, …, *N*_*R*_. Forming vectors *v* = (*v*_1_, …, *v*_*N*_*R*__)^*T*^ and *x* = (*x*_1_, …, *x*_*N*_*X*__)^*T*^ gives an ODE model of the system

$$\begin{array}{ccc} \dot{x}\left(t\right) & = & Sv(x(t)),\\ x(0) & = & x_{0}, \end{array}$$ (1)

where the stoichiometric matrix *S* maps reaction rates to the rate of change of concentrations.

The layered decomposition strategy [16, 17] defines *N*_*L*_ new stoichiometric matrices $S^{1},\ldots ,S^{N_{L}}$ such that $S=S^{1}+\cdots +S^{N_{L}}$, and defines *N*_*L*_ associated state variables *x* ^*l*^ taking values $x^{l}(t)\in {\mathbb{R}}^{N_{X}}$ (see Fig 1B). Each layer’s state *x* ^*l*^ has dynamics

$$\begin{array}{c} {\dot{x}}^{l}\left(t\right)=S^{l}v\;(x_{0}+x^{l}\left(t\right)+\sum_{k\neq l}x^{k}\left(t\right)), \end{array}$$ (2)

from initial conditions *x* ^*l*^(0) = 0, for *l* = 1, …, *N*_*L*_. The original state’s dynamics are recovered by summing the layers’ states $x(t)=x_{0}+\sum_{l=1}^{N_{L}}x^{l}(t)$. Denote by *r* = rank(*S*) the dimension of the original system, which defines the dimension of the manifold in ${\mathbb{R}}^{N_{X}}$ in which *x*(*t*) evolves. It follows that *r* ^*l*^ = rank(*S* ^*l*^) defines the dimension of the state space of each layer. Hence, even though the state space of each layer is also embedded in ${\mathbb{R}}^{N_{X}}$, each layer is a lower-dimensional system than the original system if *r* ^*l*^ < *r*.

In our previous work, we have applied this decomposition strategy by choosing the matrices *S* ^*l*^ to reflect timescale separation [17], and to reflect the propagation of steady-state responses to parametric perturbations [16]. A feature of both of these approaches was that, in the form (2), each layer’s dynamics depend on all other layers’ states (as in Fig 1B, for example). Consequently, all layers had to be numerically integrated together, and the effect of one specific layer on all others could not be easily determined. Also, the approach was constrained to define layers by strict partitions of the reaction set, somewhat limiting its flexibility to capture the widest possible range of functional subsystems. In this article, we significantly extend the layering framework in two ways. First, we introduce the concept of functionalities, which are possibly overlapping sets of reactions working together for a common purpose. To enforce a cascade structure between the functionalities, we adapt the layered dynamics corresponding to each functionality depending on its position in the cascade. The following section will use this cascade structure to define the incremental dynamic effect of each functionality.

### Mapping Functionalities to Layers

Let a functionality *F* ^*i*^ of a network be defined for *i* = 1, …, *N*_*L*_ to be a subset of $N_{R}^{i}$ reactions *F* ^*i*^ ⊆ {*R*_1_, …, *R*_*N*_*R*__} necessary to fulfil a given task of the network, where superscript integers index functionalities and their properties. It is assumed for the remainder of this section that these subsets are given, and that all reactions take part in at least one functionality.

The question of how to choose each subset *F* ^*i*^ ⊆ {*R*_1_, …, *R*_*N*_*R*__} remains out of the scope of this work. Nevertheless, there are numerous non-modular decomposition strategies taken in recent related research that we can use to justify this definition of a functionality. For example, Oishi and Klavins [37] identify control blocks as specific groups of reactions, connected by shared species. Kurata *et al*. [38] identified reaction groups forming ‘flux modules’ in the *Escherichia coli* heat shock response system. Similarly, the decomposition of signalling networks into component pathways exhibiting crosstalk [8] also identifies functionalities as groups of reactions. Finally, we can also consider elementary flux modes (EFMs) of metabolic networks [13] as being sets of reactions with the specific ‘task’ of converting one or more substrates into given products. Several of these examples are explored further in the Results section of this paper.

In this section, we will assume that the functionalities are ordered by their index $F^{1},\ldots ,F^{N_{L}}$. We first identify the dynamics of the isolated functionality *F* ^1^ as the dynamics of a biomolecular network consisting of only the reactions associated with *F* ^1^. We then identify the *conditional dynamics* of the next functionality in the cascade as the effect of extending the pre-existing network with the reactions in the new functionality.

First consider, without loss of generality, the network defined by only the subset of reactions making up functionality *F* ^1^ ⊆ {*R*_1_, …, *R*_*N*_*R*__}, taken in isolation from the other reactions. For given initial conditions *x*_0_, we now identify the isolated dynamics of this functionality as the solution to the layer

$$\begin{array}{ccc} {\dot{x}}^{1}\left(t\right) & = & S^{1}v\left(x_{0}+x^{1}\left(t\right)\right),\\ x^{1}\left(0\right) & = & 0. \end{array}$$ (3)

Here, the stoichiometric matrix *S* ^1^ is defined

$$\begin{array}{c} S_{jk}^{1}=\{\begin{array}{cc} S_{jk} & R_{k}\in F^{1},\\ 0 & \text{otherwise,} \end{array} \end{array}$$

by copying the columns of the original stoichiometric matrix *S* in (1) corresponding to the reactions in *F* ^1^ and setting the other columns to zero. We will denote this trajectory *x* ^1^ = *L*(*F* ^1^), where the notation *L* represents a map from the functionality *F* ^1^ to the solution *x* ^1^ of the dynamics (3) from initial conditions *x* ^1^(0) = 0.

Note that *L*(*F* ^1^) depends on the specific initial condition *x*_0_ of the network (3), which is in general distinct from the initial condition *x* ^1^(0) = 0 of the layer’s state. To make this dependence explicit, it is sometimes helpful (see Examples 2 and 3) to define a ‘zero layer’ *F* ^0^ with constant trajectory *L*(*F* ^0^) = *x*_0_. We can then make clear that *L*(*F* ^1^) is dependent on the initial conditions by writing it as *L*(*F* ^1^∣*F* ^0^). The layered framework also implies that the absolute concentrations in this network are modelled by the translated trajectory *x*_0_ + *L*(*F* ^1^∣*F* ^0^).

We next consider extending the functionality *F* ^1^ by combining it with the reactions in *F* ^2^. The extended network can be simulated through a similar process to the original network above, as follows. Define *S* ^1,2^ as

$$\begin{array}{c} S_{jk}^{1,2}=\{\begin{array}{cc} S_{jk} & R_{k}\in F^{1}\cup F^{2},\\ 0 & \text{otherwise,} \end{array} \end{array}$$

considering only the reactions in at least one of *F* ^1^ or *F* ^2^. Using *S* ^1,2^ we can then simulate the layer corresponding to the extended network

$$\begin{array}{ccc} {\dot{x}}^{1,2}\left(t\right) & = & S^{1,2}v\left(x_{0}+x^{1,2}\left(t\right)\right),\\ x^{1,2}\left(0\right) & = & 0, \end{array}$$ (4)

the solution of which can be written *L*(*F* ^1^, *F* ^2^∣*F* ^0^) = *x* ^1,2^. This denotes the trajectory of the combined functionalities *F* ^1^ and *F* ^2^.

The fact that each of (3) and (4) are layers with states in ${\mathbb{R}}^{N_{X}}$ implies that we can calculate the difference between each of the trajectories. This difference is clearly interpreted as the incremental effect of extending a network made up of the initial conditions *F* ^0^ and the isolated functionality *F* ^1^, by also including *F* ^2^. We thus define

$$\begin{array}{c} L\left(F^{2}|F^{1},F^{0}\right)=L\left(F^{1},F^{2}|F^{0}\right)-L\left(F^{1}|F^{0}\right) \end{array}$$ (5)

as the *conditional dynamics* of *F* ^2^, given the specified context of *F* ^1^ and the initial condition layer *F* ^0^.

However, rather than simulating the layer (4) representing the combined functionalities, we may further exploit the layered framework described above to directly simulate *L*(*F* ^2^∣*F* ^1^, *F* ^0^). Suppose we already have *x* ^1^ = *L*(*F* ^1^∣*F* ^0^), found as the solution to the dynamics (3). We now define the layer

$$\begin{array}{ccc} {\dot{x}}^{2}\left(t\right) & = & S^{1,2}v\left(x_{0}+x^{1}\left(t\right)+x^{2}\left(t\right)\right)-S^{1}v\left(x_{0}+x^{1}\left(t\right)\right),\\ x^{2}\left(0\right) & = & 0, \end{array}$$ (6)

with *S* ^1^ and *S* ^1,2^ given above. Note that this layer is downstream of (3), since it depends on the state *x* ^1^. It is clear from summing the vector fields in (3) and (6) that the sum (*x* ^1^ + *x* ^2^) of the layers’ states follows exactly the same dynamics as the combined network’s state *x* ^1,2^ in (4). Thus, since *x* ^2^ = *x* ^1,2^ − *x* ^1^ it follows that the dynamics (6) directly simulate *L*(*F* ^2^∣*F* ^1^, *F* ^0^), with the input *L*(*F* ^1^∣*F* ^0^) simulated by (3).

We can rewrite the dynamics (6) corresponding to the simulation of *L*(*F* ^2^∣*F* ^1^, *F* ^0^) as

$$\begin{array}{c} {\dot{x}}^{2}\left(t\right)=S^{2}v\left(x_{0}+x^{1}\left(t\right)+x^{2}\left(t\right)\right)+S^{1}v_{alt}\left(x_{0}+x^{1}\left(t\right),x^{2}\left(t\right)\right) \end{array}$$ (7a)

where *S* ^2^ = *S* ^1,2^ − *S* ^1^ corresponds to the reactions in *F* ^2^\*F* ^1^, and

$$\begin{array}{c} v_{alt}\left(x_{0}+x^{1},x^{2}\right)=v\left(x_{0}+x^{1}+x^{2}\right)-v\left(x_{0}+x^{1}\right) \end{array}$$ (7b)

are the rates of the ‘altered reactions’: the rates of reactions in *F* ^1^ which are modified by the presence of *F* ^2^ (shown as broken green arrows in Fig 1C). This description allows us to see the degree to which *F* ^2^ is ‘downstream’ of *F* ^1^. For example, if *v*_*alt*_ = 0, then we can say that the reactions in *F* ^1^ are independent of those in *F* ^2^ and that *F* ^2^ is strictly downstream of *F* ^1^. Note that, especially for larger networks, many of the altered reaction rates in *v*_*alt*_ are zero and can be omitted (see Example 3), simplifying simulation. Given that the trajectory of *x* ^1^ is already determined from simulating (3), we can simulate either (6) or (7a), using *x* ^1^(*t*) as a time-dependent input to obtain the conditional dynamics *L*(*F* ^2^∣*F* ^1^). The latter approach is taken in our examples (see Results section).

The definitions above easily extend to larger combinations of functionalities. In full generality, we can consider the network defined by the combination of *n*_1_ functionalities $F^{1},\ldots ,F^{n_{1}}$, and its extension through the additional *n*_2_ functionalities $F^{n_{1}+1},\ldots ,F^{n_{1}+n_{2}}$. By writing ${\bar{F}}^{1}=F^{1}\cup \cdots \cup F^{n_{1}}$ and ${\bar{F}}^{2}=F^{n_{1}+1}\cup \cdots \cup F^{n_{1}+n_{2}}$, the definitions above can apply in the simulation of

$$\begin{array}{ccc} L(F^{1},\ldots ,F^{n_{1}}|F^{0}) & = & L({\bar{F}}^{1}|F^{0}),\\ L(F^{n_{1}+1},\ldots ,F^{n_{1}+n_{2}}|F^{0},F^{1},\ldots ,F^{n}) & = & L({\bar{F}}^{2}|F^{0},{\bar{F}}^{1}). \end{array}$$

Here we have defined a notation for the trajectory $L(F^{1},\ldots ,F^{n_{1}}|F^{0})$ of the biochemical network made up of the reactions which comprise an arbitrary combination of functionalities $F^{1},\ldots ,F^{n_{1}}$. We have also defined the change in trajectory $L(F^{n_{1}+1},\ldots ,F^{n_{1}+n_{2}}|F^{0},F^{1},\ldots ,F^{n_{1}})$ incurred by extending that network with the additional reactions in $F^{n_{1}+1},\ldots ,F^{n_{1}+n_{2}}$. Finally, we have shown how to identify the dynamical systems that can simulate these trajectories.

Consider the two layers *L*(*F* ^2^∣*F* ^1^) and *L*(*F* ^2^) that both describe the effect of the functionality *F* ^2^. This effect is different depending on the presence or absence of *F* ^1^. The difference between these two trajectories defines how the presence of *F* ^1^ changes the behaviour of *F* ^2^; that is, the dependence of *F* ^2^ on *F* ^1^. We will now demonstrate how our layered analysis allows us to define the interdependence between two functionalities, thereby capturing the nonlinear effects arising from modelling a biomolecular network as being constructed from a combination of functional subsystems.

### Calculating with Layers

In order to quantify the interactions between functionalities, we can exploit the layered formulation above. For simplicity, from this point on we suppress the *F* ^0^ notation, with the acknowledgement that all of the trajectories depend on the system’s initial conditions *L*(*F* ^0^) = *x*_0_. The definition of conditional dynamics in (5) implies that *L*(*F* ^1^, *F* ^2^) = *L*(*F* ^2^∣*F* ^1^) + *L*(*F* ^1^). This represents a layered cascade, where the dynamics of an integrated network are the linear combination of the conditional dynamics of its functionalities. There are two natural questions associated with this approach. First, how is the contribution of functionality *F* ^2^, considered in isolation, different from the conditional dynamics of *F* ^2^ when integrated with *F* ^1^? Secondly, how is the behaviour of the integrated *F* ^1^, *F* ^2^ network different from the linear combination of the isolated functionalities? That is, how different is *L*(*F* ^1^, *F* ^2^) from *L*(*F* ^1^) + *L*(*F* ^2^)?

The answers to these two questions are the same. We denote the error incurred by approximating the integrated system as the linear combination of the isolated dynamics by the quantity *M*(*F* ^1^; *F* ^2^), defined as

$$\begin{array}{c} M\left(F^{1};F^{2}\right)=L\left(F^{1}\right)+L\left(F^{2}\right)-L\left(F^{1},F^{2}\right), \end{array}$$

which we call *mutual dynamics*. This can be interpreted as the nonlinearity that arises from integrating the two functionalities together. Note that, since *M* is defined symmetrically, we can use (5) to rewrite *M* as

$$\begin{array}{c} M\left(F^{1};F^{2}\right)=L\left(F^{2}\right)-L\left(F^{2}|F^{1}\right)=L\left(F^{1}\right)-L\left(F^{1}|F^{2}\right). \end{array}$$ (8)

Therefore *M* measures how the function of *F* ^2^ (or *F* ^1^) is changed when considered in the context of *F* ^1^ (or *F* ^2^). Thus *M*(*F* ^1^; *F* ^2^) is a symmetric measure of the interdependence between the two functionalities. We use (8) to calculate the the mutual dynamics between two functionalities, which requires us to first obtain either both trajectories *L*(*F* ^1^) and *L*(*F* ^1^∣*F* ^2^), or alternatively both trajectories *L*(*F* ^2^) and *L*(*F* ^2^∣*F* ^1^). These trajectories can be simulated, as described in the previous section, or calculated by the methods described in ‘Reducing Computational Burden’ below.

**Fig 2:**
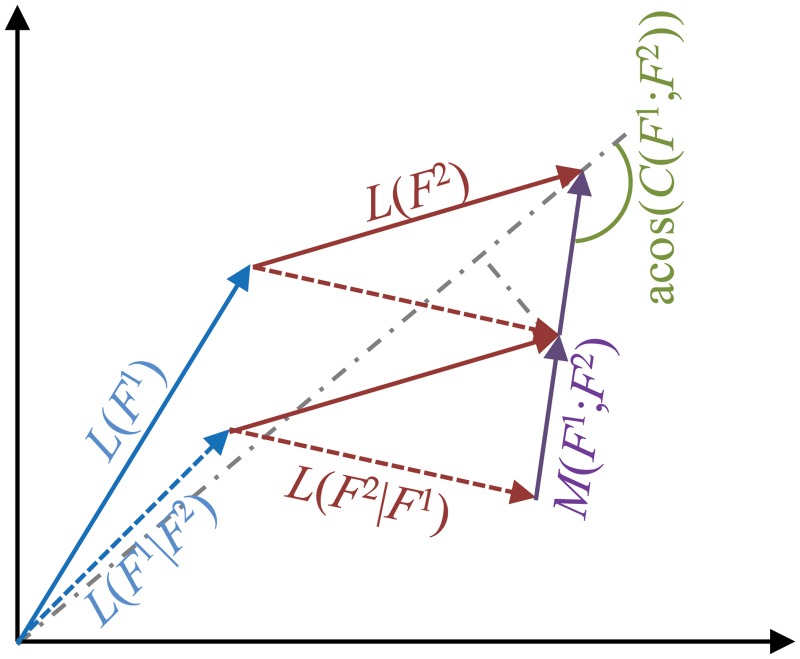
(Conditional) layer dynamics, mutual dynamics, incompatibility, and cooperativity. The joint dynamics *L*(*F* ^1^, *F* ^2^) is equal to the sum of the isolated dynamics *L*(*F* ^1^) [solid blue] and the conditional dynamics *L*(*F* ^2^∣*F* ^1^) [dashed red], or *vice versa* *L*(*F* ^2^) + *L*(*F* ^1^∣*F* ^2^) [solid red and dashed blue]. The mutual dynamics *M*(*F* ^1^; *F* ^2^) [solid lilac] corresponds to the error made when approximating the joint dynamics *L*(*F* ^1^, *F* ^2^) with the sum of isolated dynamics *L*(*F* ^1^) + *L*(*F* ^2^). The cooperativity *C*(*F* ^1^; *F* ^2^) is the cosine of the angle between −*M* and the sum of the isolated dynamics. In the example shown, the cooperativity is negative, indicating that the isolated behaviour *L*(*F* ^1^) + *L*(*F* ^2^) is attenuated when *F* ^1^ and *F* ^2^ are integrated together, although also with some orthogonal effects. The strength of their interaction is proportional to the incompatibility *I*(*F* ^1^; *F* ^2^), defined as the ratio between the lengths of *M*(*F* ^1^; *F* ^2^) and *L*(*F* ^1^) + *L*(*F* ^2^).

We have been careful to make explicit through our Bayesian-style notation that the dynamics of all functionalities are context-dependent. It is also the case that the interdependence between any two functionalities is context-dependent. Therefore, we need to extend the definition of mutual dynamics to consider how the interdependence between *F* ^1^ and *F* ^2^ is dependent on the wider context of the network, which we denote by another functionality, *F* ^3^. Similarly to the definition above, we can define the *conditional mutual dynamics* between *F* ^1^ and *F* ^2^, given *F* ^3^, with the formula

$$\begin{array}{c} M\left(F^{1};F^{2}|F^{3}\right)=L\left(F^{1}|F^{3}\right)+L\left(F^{2}|F^{3}\right)-L\left(F^{1},F^{2}|F^{3}\right), \end{array}$$

to quantify the difference between the dynamics of the integrated and isolated functionalities *F* ^1^ and *F* ^2^, in the context of *F* ^3^. As before, this can also be expressed in terms of layered dynamics as

$$\begin{array}{c} M\left(F^{1};F^{2}|F^{3}\right)=L\left(F^{2}|F^{3}\right)-L\left(F^{2}|F^{1},F^{3}\right)=L\left(F^{1}|F^{3}\right)-L\left(F^{1}|F^{2},F^{3}\right), \end{array}$$

to quantify how the effect of *F* ^2^ on its context changes with the presence of *F* ^1^, and vice versa. The geometric intuition underlying the conditional mutual dynamics can be seen in Fig 2. The key interpretation of *M* is that it captures the nonlinearities that arise from combining *F* ^1^ and *F* ^2^ into a single network, conditioned on *F* ^3^ if necessary.

The conditional mutual dynamics *M*(*F* ^1^; *F* ^2^∣*F* ^3^) is a time-varying, vector trajectory. We can base on *M* the following time-varying scalar, which we call the *incompatibility* and denote *I*(*F* ^1^; *F* ^2^∣*F* ^3^) with formula

$$\begin{array}{c} I\left(F^{1};F^{2}|F^{3}\right)=\frac{∥M(F^{1};F^{2}|F^{3})∥}{∥L\left(F^{1}|F^{3}\right)+L\left(F^{2}|F^{3}\right)∥}. \end{array}$$ (9)

Here, ‖.‖ represents the Euclidean norm. In this paper we use the unweighted Euclidean norm, but in certain cases it might be appropriate to introduce a weight, for example if the concentrations of the species in a network are at different orders of magnitude. One might also decide to set the weight of certain intermediate species of limited interest to zero (see below).

This incompatibility measures the relative size of the error made by approximating the integration of two functionalities as the sum of their individual behaviour. To gain some intuition about this number, we can consider a number of special cases. If *I*(*F* ^1^; *F* ^2^∣*F* ^3^) = 0, then this indicates that the trajectory of the integrated functionalities is simply the sum of the isolated functionalities’ trajectories: *L*(*F* ^1^, *F* ^2^∣*F* ^3^) = *L*(*F* ^1^∣*F* ^3^) + *L*(*F* ^2^∣*F* ^3^). If the incompatibility is nonzero but small then *L*(*F* ^1^, *F* ^2^∣*F* ^3^) ≈ *L*(*F* ^1^∣*F* ^3^) + *L*(*F* ^2^∣*F* ^3^) is a reasonable approximation, since the incurred error is relatively small. However, if *I* is of significant size, then the dynamics of the integrated functionalities can be expected to significantly differ from their individual behaviours.

Besides *I*, which measures the relative size of the mutual dynamics *M*, the direction of *M* is also important, since this determines if the integration of two functionalities together enhances or attenuates their individual dynamics, or causes other effects. We define the cooperativity *C* as the cosine of the angle between −*M* and the sum of the isolated layers:

$$\begin{array}{c} C\left(F^{1};F^{2}|F^{3}\right)=-\frac{M\left(F^{1};F^{2}|F^{3}\right)\cdot \left(L\left(F^{1}|F^{3}\right)+L\left(F^{2}|F^{3}\right)\right)}{∥M\left(F^{1};F^{2}|F^{3}\right)∥∥L\left(F^{1}|F^{3}\right)+L\left(F^{2}|F^{3}\right)∥}, \end{array}$$ (10)

with ⋅ denoting the scalar product. See Fig 2 for a geometric representation of *C*. Note that when using a weighted norm, the scalar product should be weighted accordingly. Again, we consider a number of special cases. Suppose that *C*(*F* ^1^; *F* ^2^∣*F* ^3^) equals or is close to minus one, so that *M* is approximately parallel to and pointing in the same direction as *L*(*F* ^1^∣*F* ^3^) + *L*(*F* ^2^∣*F* ^3^). In this case the integrated dynamics can be approximated as an attenuation of the isolated behaviour

$$\begin{array}{ccc} L(F^{1},F^{2}|F^{3}) & = & L\left(F^{1}|F^{3}\right)+L\left(F^{2}|F^{3}\right)-M\left(F^{1};F^{2}|F^{3}\right)\\ & \approx & (1-I(F^{1};F^{2}|F^{3}))(L\left(F^{1}|F^{3}\right)+L\left(F^{2}|F^{3}\right)). \end{array}$$

Conversely, if the cooperativity equals or is close to one, the two functionalities enhance each other, in the sense that we can approximate the integrated behaviour as an amplification of the isolated behaviours

$$\begin{array}{c} L\left(F^{1},F^{2}|F^{3}\right)\approx (1+I(F^{1};F^{2}|F^{3}))(L\left(F^{1}|F^{3}\right)+L\left(F^{2}|F^{3}\right)). \end{array}$$

However, once the cooperativity *C* equals or is close to zero, the mutual dynamics *M* are orthogonal to *L*(*F* ^1^∣*F* ^3^) + *L*(*F* ^2^∣*F* ^3^). This means that when the functionalities are integrated, both isolated functionalities are maintained, but there are also additional interactions (with an effect of strength *I*) in directions orthogonal to the summed isolated dynamics. For example, suppose that the functionalities *F* ^1^ and *F* ^2^ correspond to sets of reactions responsible for mediating the cellular responses to two different input signals. By setting the influence of all but the common output (measured) species to zero, an incompatibility *I*(*F* ^1^; *F* ^2^) close to zero corresponds to an additive interaction of the input signals. If *I*(*F* ^1^; *F* ^2^) is larger, it may correspond to either a synergetic (for *C*(*F* ^1^; *F* ^2^) = 1) or antagonistic (for *C*(*F* ^1^; *F* ^2^) = −1) interaction (see e.g. [28]). Note that for a scalar output, orthogonal dynamics are not possible.

For systems composed of many different functionalities, one might also take the time averages ⟨*I*⟩ and ⟨*C*⟩ of the incompatibility, respectively the cooperativity, over the simulation time Δ*T* to obtain single measures quantifying the interactions between layers:

$$\begin{array}{ccc} \langle I\rangle \;\left(F^{1};F^{2}|F^{3}\right) & = & \frac{1}{\Delta T}\int_{0}^{\Delta T}I\left(F^{1};F^{2}|F^{3}\right)dt\\ \langle C\rangle \;\left(F^{1};F^{2}|F^{3}\right) & = & \frac{1}{\Delta T}\int_{0}^{\Delta T}C\left(F^{1};F^{2}|F^{3}\right)dt \end{array}$$

Although they are useful for obtaining a first impression of how functionalities interact, time averages should be carefully applied. They can hide transient interactions between functionalities, including potential sign changes of the state-dependent cooperativities (see Example 1).

We have now identified how to measure the interdependence between two functionalities, which we have defined as the change in the dynamics of one functionality when the other is present. We have made explicit how this interdependence is itself dependent on the context of the rest of the network. In the remainder of this section we will describe how to minimise the computational burden incurred when calculating all possible interactions between functionalities.

### Reducing Computational Burden

The notation *L*(*F* ^1^) and *L*(*F* ^2^∣*F* ^1^) describing the map from a functionality (or set of functionalities) to the resulting trajectory simplifies the calculations we may wish to carry out to understand how the functionalities combine. Using the key definition of ‘conditional dynamics’ given by (5), we can prove a number of rules for combining layers which appear analogous to those known from Information Theory [39]. For example, for two random variables *X* and *Y*, the well-known quantities of joint entropy *H*(*X*, *Y*) = *H*(*X*) + *H*(*Y*∣*X*) and mutual information *I*(*X*; *Y*) = *H*(*X*) − *H*(*X*∣*Y*) are each definitions of the same form as those given above of conditional dynamics (5) and mutual dynamics (8) respectively. However, it is important to note that this similarity is only superficial, and any intuition gained by seeking analogies between our work and information theoretic concepts should be applied carefully. This caveat applies in particular to the two results below.

Two lemmas allowing the quick combination of layer dynamics can be easily proved directly from the definitions of *L*(*F* ^1^) and *L*(*F* ^2^∣*F* ^1^), their extensions to larger combinations of functionalities, and Eq (5). The first is an analogue of Bayes’ Rule, given by

$$\begin{array}{c} L\left(F^{1}|F^{2},F^{3}\right)=L\left(F^{2}|F^{1},F^{3}\right)+L\left(F^{1}|F^{3}\right)-L\left(F^{2}|F^{3}\right). \end{array}$$ (11)

We will demonstrate how this rule can be used for quickly deducing the incremental effects of layers when combined in a different order. This is fundamental, since a natural ordering of the layers is generally not given. A second rule, which is analogous to Bayes’ Factor, is given by

$$\begin{array}{c} \underset{\text{Posterior}\;\text{Dynamics}}{\underset{︸}{L\left(F^{3}|F^{1}\right)-L\left(F^{2}|F^{1}\right)}}=\underset{\text{Bayes}\;\text{Factor}\;B_{32|1}}{\underset{︸}{L\left(F^{1}|F^{3}\right)-L\left(F^{1}|F^{2}\right)}}+\underset{\text{Prior}\;\text{dynamics}}{\underset{︸}{L\left(F^{3}\right)-L\left(F^{2}\right)}}. \end{array}$$ (12)

This rule applies when we have a choice between integrating two functionalities to the *F* ^1^-only network. The difference in their effects is decomposed into the difference *L*(*F* ^3^) − *L*(*F* ^2^) between their isolated behaviours, summed with the difference *L*(*F* ^1^∣*F* ^3^) − *L*(*F* ^1^∣*F* ^2^) in the incremental effect of *F* ^1^ on each. We can use these rules to compute all possible functionality combinations with a minimal amount of simulation.

In order to answer particular biological questions, we may be interested in the incremental effect of a given functionality on a specific ‘base’ network, such as those described in Examples 1 and 2 in the *Results* section. In other cases we may be interested in all possible interactions between the functionalities, such as the situation in Example 3. In a biochemical network whose reactions are decomposed into *N*_*L*_ functionalities, the latter case suggests that we must simulate all *N*_*L*_ layers for each of the *N*_*L*_! different orderings of the functionalities, resulting in (*N*_*L*_ + 1)! − *N*_*L*_! layers to be numerically solved. This burden can be significantly reduced using the calculation rules deduced above.

**Fig 3:**
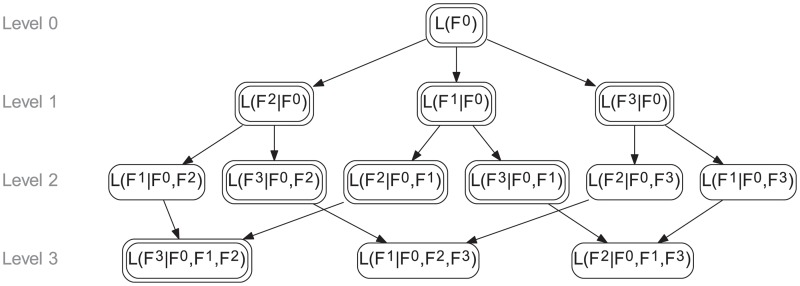
Layering graph with the (conditional) dynamics of *N*_*L*_ = 3 functionalities in all orders. The root of the graph at Level 0 represents the initial conditions of the network (*L*(*F* ^0^) = *x*_0_, with *F* ^0^ = {}). Each path from the root to a leaf represents a different ordering of the functionalities *F* ^1^, *F* ^2^, and *F* ^3^. The nodes correspond to the layers for a given ordering of functionalities. Due to symmetries, only seven of the twelve (conditional) layer dynamics have to be obtained by integration of the respective models of the layers to get to know the dynamics of all layers in all orderings. A possible choice for these layer dynamics is indicated by double boundaries around the respective nodes. The dynamics of the other layers can be obtained by applying Bayes’ rule. Graph drawn with Graphviz [57].

The cascaded layers representing all possible orderings of functionalities can be arranged in an acyclic directed graph, shown in Fig 3. Each node represents the trajectory arising from the incremental addition of a new functionality, given those already present. The graph is organised into levels, corresponding to the position of the new layer in the sequence. The root of the layering graph (referred to as Level 0) represents the given initial conditions *x*_0_. Each of the subsequent levels *l* = 1, …, *N*_*L*_ consists of $\left(N_{L}-l+1\right)\;\left(\begin{array}{l} N_{L}\\ l-1 \end{array}\right)$ nodes. Each node represents the dynamics of a functionality *F* ^*i*^ conditioned on a subset of size *l* − 1 of the remaining functionalities *F* ^*j*^, *j* ≠ *i*. A directed edge from a node in Level *l* to a node in Level *l* + 1 exists if the node in Level *l* + 1 is conditioned on all functionalities taking part in the node in Level *l*.

Each directed path from Level 0 to Level *N*_*L*_ (i.e. from the root to a leaf) represents one of the *N*_*L*_! possible orderings of functionalities. By adding up the layer dynamics corresponding to the nodes in each path, the trajectory of the complete network is obtained. Each node in Level 1 represents the dynamics of an isolated functionality. The dynamics represented by the nodes at the leaves of the layering graph are also of specific interest. Multiplying the layer dynamics in a given node in Level *N*_*L*_ (corresponding to a particular functionality *F* ^*i*^) by −1 gives the change in the dynamics that results from *removing* *F* ^*i*^ from the system while keeping all other functionalities intact (see Example 3).

To obtain the dynamics of all layers for all orderings of functionalities, we can use rules (11) and (12) to exploit certain symmetries in the layering graph and reduce the number of numerical integrations of layer ODE systems. If all of the dynamics in Level *l* − 1 are already known, it is only necessary to numerically solve $\left(\begin{array}{c} N_{L}\\ l \end{array}\right)$ layer dynamics in Level *l*. The rest of the level can then be deduced using Bayes’ rule (11). For example, suppose we have the trajectories of all nodes in Layer 1, and have simulated *L*(*F* ^1^∣*F* ^0^, *F* ^2^) in Level 2. Using (11) we can deduce *L*(*F* ^2^∣*F* ^0^, *F* ^1^) without having to simulate again. In fact, when considering a network with *N*_*L*_ functionalities, only

$$\begin{array}{c} \sum_{l=1}^{N_{L}}(\begin{array}{c} N_{L}\\ l \end{array})=2^{N_{L}}-1 \end{array}$$

layers have to be numerically integrated (see Example 1), compared to (*N*_*L*_ + 1)! − *N*_*L*_! integrations necessary when analyzing all orderings separately. Although the number of integrations still exponentially grows with *N*_*L*_, it nevertheless becomes possible to analyse systems with up to ten or more distinct functionalities in reasonable time. For *N*_*L*_ = 10, integrating every layer would require more than 3.5 ⋅ 10^4^ times as much computational time as is required by exploiting (11).

Furthermore, the symmetry of the layering graph allows a certain degree of freedom in choosing which layers to simulate, and which to deduce from (11). In particular, one should always choose to simulate the simplest layers, with respect to the number of states or reactions in the respective ODE system. Fig 3 shows the situation where *N*_*L*_ = 3: we need to simulate 2^3^ − 1 = 7 layers. We have decided to, wherever possible, simulate *F* ^3^, ahead of *F* ^2^, ahead of *F* ^1^. After simulating the isolated behaviour *L*(*F* ^1^∣*F* ^0^) of the functionality *F* ^1^, we can calculate its behaviour in any other context without having to simulate it again. For example, *L*(*F* ^1^∣*F* ^0^, *F* ^2^) can be calculated, using (11), as *L*(*F* ^2^∣*F* ^0^, *F* ^1^) + *L*(*F* ^1^∣*F* ^0^) − *L*(*F* ^2^∣*F* ^0^). Similarly, substituting the resulting trajectory into (11) once more means that we can calculate *L*(*F* ^1^∣*F* ^0^, *F* ^2^, *F* ^3^) as the linear combination *L*(*F* ^3^∣*F* ^0^, *F* ^1^, *F* ^2^) + *L*(*F* ^1^∣*F* ^0^, *F* ^2^) − *L*(*F* ^3^∣*F* ^0^, *F* ^2^). As can be seen by the indicated nodes in Fig 3, only one layer corresponding to *F* ^1^ had to be numerically simulated, whereas we simulated layers corresponding to *F* ^2^ twice, and layers corresponding to *F* ^3^ four times. Thus we can reduce computation time even further by avoiding the repeated simulation of high-dimensional layers.

## Results

We now apply our layered approach to three examples of multi-functional biomolecular systems. In each case, we can show how the effect of each functionality in a network is dependent on the others. We exploit the formulation of mutual dynamics to quantify the interdependence between different functionalities.

### Example 1: Crosstalk

The high osmolarity glycerol and the pheromone response mitogen-activated protein (MAP) kinase pathways in *S. cerevisiae* share the common species *Ste11* [40]. Such a common species can constitute a mutually excitatory crosstalk mechanism by cross-activating one pathway upon activation of the other. However, McClean *et al*. [8] observed that only one pathway responds, and deduced that a second, mutually inhibitory crosstalk mechanism exists. They constructed a model, available as Model 115 in the BioModels Database [41], that includes both crosstalk mechanisms.

**Fig 4:**
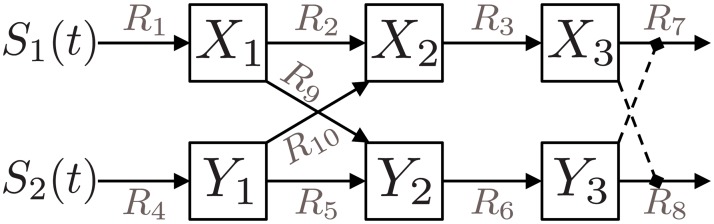
Signalling pathways with crosstalk. This figure shows two signalling pathways, with two crosstalk mechanisms. Functionality *F* ^1^ = {*R*_1_, *R*_2_, *R*_3_} is the *X* pathway; *F* ^2^ = {*R*_4_, *R*_5_, *R*_6_} is the *Y* pathway; the mutually inhibiting crosstalk *F* ^3^ = {*R*_7_, *R*_8_}; and the mutually excitatory crosstalk *F* ^4^ = {*R*_9_, *R*_10_}. Model adopted from [8].

As an initial example of our approach we implemented a layered decomposition of this model, shown in Fig 4. The mathematical description of the complete model and of all layers can be found in the Supporting Information. We applied our framework to systematically investigate the function of each of the crosstalk mechanisms. We first considered the effects of each crosstalk mechanism on the signalling pathways. This gives some indication of the function of each crosstalk mechanism, but our framework takes this analysis further. The effect of excitatory crosstalk on the network is altered by the presence of inhibitory crosstalk, and *vice versa*. The conditional mutual dynamics between the two crosstalk mechanisms, integrated with the crosstalk-free network, can be used to quantify their interdependence.

In the model, we set the strength of the mutually excitatory crosstalk to *k*_*a*_ = 0.1 and the strength of the mutually inhibitory crosstalk to *k*_*d*_ = 1, corresponding to a monostable network (see Fig. 1 in [8]). We assume zero initial conditions. The three reactions *R*_1_, *R*_2_, and *R*_3_ represent the first signal transduction pathway, which affects species concentrations *X*_1_–*X*_3_, so that we define the functionality *F* ^1^ = {*R*_1_, *R*_2_, *R*_3_}. Similarly, we choose *F* ^2^ = {*R*_4_, *R*_5_, *R*_6_} to represent the second signal transduction pathway, affecting species *Y*_1_–*Y*_3_. The mutually inhibiting crosstalk between the two pathways comprises *F* ^3^ = {*R*_7_, *R*_8_}. Finally, the mutually excitatory crosstalk is given by *F* ^4^ = {*R*_9_, *R*_10_}.

**Fig 5:**
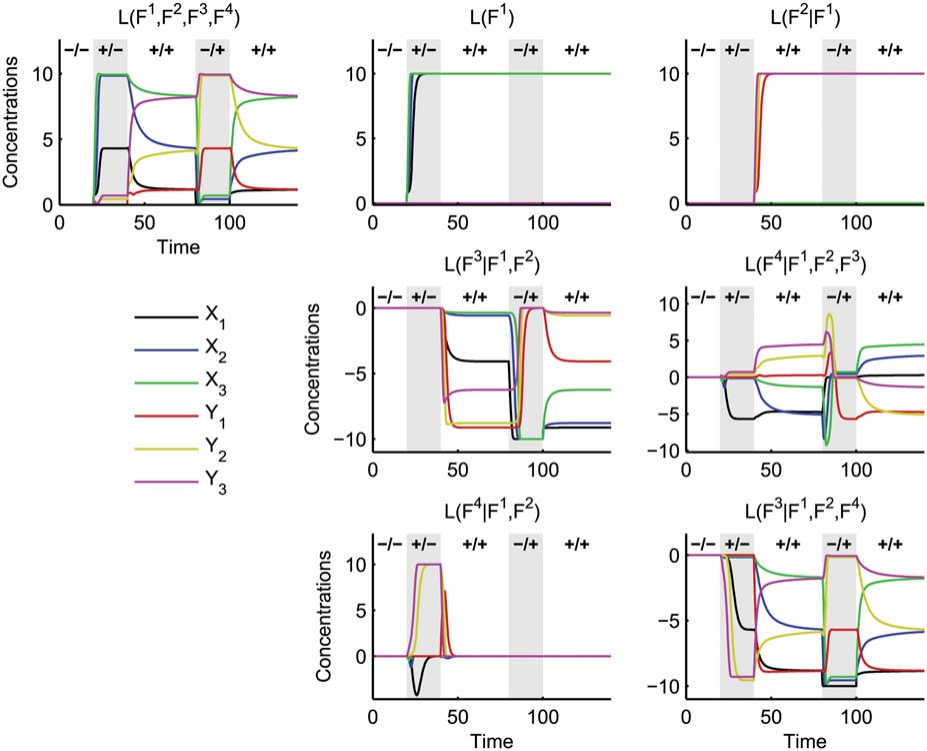
Layer dynamics of the crosstalk example. Dynamics of the layers for switching between different input combinations: (-/-) *S*_1_ = *S*_2_ = 0; (+/-) *S*_1_ = 5, *S*_2_ = 0; (+/+) *S*_1_ = *S*_2_ = 5; and (-/+) *S*_1_ = 0, *S*_2_ = 5. The top left figure corresponds to the dynamics of the complete network, the central and right top figure to the dynamics of the two isolated signalling pathways without any crosstalk. If the mutually inhibitory crosstalk *L*(*F* ^3^∣*F* ^1^, *F* ^2^) is added first, the network becomes bistable, then monostable again by then including the mutually excitatory crosstalk *L*(*F* ^4^∣*F* ^1^, *F* ^2^, *F* ^3^). Adding first the excitatory crosstalk *L*(*F* ^4^∣*F* ^1^, *F* ^2^) leads to strong cross-activation of the pathways, which is significantly weakened by the mutual inhibitory crosstalk *L*(*F* ^3^∣*F* ^1^, *F* ^2^, *F* ^4^).

We first calculated all of the possible incremental effects of each functionality *F* ^*i*^ integrated with each possible subset {*F* ^*j*^ ∣ *j* ≠ *i*} of the others. Since we have *N*_*L*_ = 4 functionalities we only need to simulate $2^{N_{L}}-1=15$ layers to be able to calculate all *N*_*L*_ ⋅ *N*_*L*_! = 96 layer dynamics for all orderings of the functionalities. Fig 5 shows the trajectories corresponding to several of these nodes. The plots were generated for time-varying inputs *S*_1_ and *S*_2_ given by

$$\begin{array}{c} \left[S_{1}\left(t\right),S_{2}\left(t\right)\right]=\{\begin{array}{cc} \left[0,0\right] & 0\leq t<20,\\ \left[5,0\right] & 20\leq t<40,\\ \left[5,5\right] & 40\leq t<80,\\ \left[0,5\right] & 80\leq t<100,\\ \left[5,5\right] & 100\leq t. \end{array} \end{array}$$

The switching times between the input combinations were chosen such that, at the end of each period, the species of the network have converged to their corresponding steady-state concentrations.

**Fig 6:**
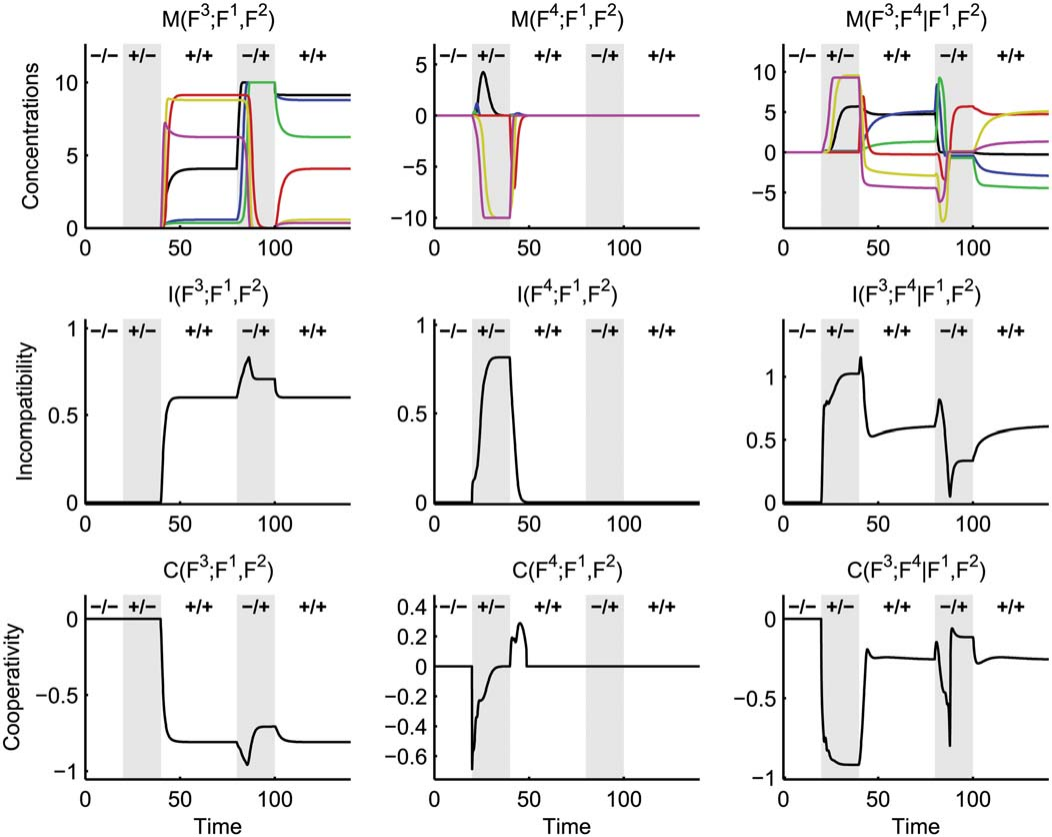
Mutual dynamics, incompatibilities and cooperativities between the layers of the crosstalk example. See the caption of Fig 5 for details. The first column considers the nonlinear interaction of mutual inhibition with the isolated pathways; the second column replaces mutual inhibition with mutual excitation; the third column captures the nonlinear effects of combining the inhibition and excitation crosstalk mechanisms.

The top-left plot in Fig 5 shows the response *L*(*F* ^1^, *F* ^2^, *F* ^3^, *F* ^4^) of the entire network to this input pattern. Each of the other plots depicts the incremental effects of integrating a certain functionality with a group of others. Fig 6 depicts the trajectories of the mutual dynamics between several pairs of functionalities, also calculated from the 15 simulations of layered ODE systems. Below these trajectories we plot their corresponding incompatibilities *I* and cooperativities *C*.

We consider the basic network comprised only of *F* ^1^ and *F* ^2^, where neither crosstalk mechanism is active. From *L*(*F* ^1^) and *L*(*F* ^2^) in Fig 5 we see that after *t* = 20 (respectively, *t* = 40) the concentrations of species *X*_1_–*X*_3_ (respectively, *Y*_1_–*Y*_3_) quickly saturate. It is easily shown that the mutual dynamics between the two isolated pathways *M*(*F* ^1^; *F* ^2^) = 0. This means that there is no interdependency between the pathways, and their integrated dynamics equal the sum of their isolated dynamics. Of course, this conclusion is intuitively clear because we are considering a network with neither crosstalk mechanism active. We are now in a position to investigate what each crosstalk mechanism does to this basic network, by identifying the effects of each of *F* ^3^ and *F* ^4^ in turn.

We first identify the effect of the mutual inhibitory crosstalk *F* ^3^ on the basic network. This is depicted in Fig 5 by *L*(*F* ^3^∣*F* ^1^, *F* ^2^). The input signals *S*_1_ = *S*_2_ = 5 are both ‘on’ during two time intervals *t* ∈ [40, 80] and *t* ≥ 100. In each of these time intervals, we can observe in *L*(*F* ^3^∣*F* ^1^, *F* ^2^) two different steady states of *X*_1_–*X*_3_ and *Y*_1_–*Y*_3_. Thus one effect of mutual inhibition is that the resulting network is bistable. We can also observe that the *X*_1_–*X*_3_ values of *L*(*F* ^3^∣*F* ^1^, *F* ^2^), which show the effect of *F* ^3^ on the *X*_*i*_ concentrations, become sufficiently negative during *t* ∈ [80, 100] that they cancel out the positive values of *X*_1_–*X*_3_ in *L*(*F* ^1^, *F* ^2^). Hence, we can conclude that, by integrating mutual inhibition with the basic network, the removal of *S* ^1^ at *t* = 80 now causes the corresponding pathway to deactivate. We can also analyse the interaction between mutual inhibition and the basic network in terms of the mutual dynamics *M*(*F* ^3^; *F* ^1^, *F* ^2^), as depicted in Fig 6. For *t* ≥ 40, the cooperativity *C*(*F* ^3^; *F* ^1^, *F* ^2^) is always negative, and the incompatibility *I*(*F* ^3^; *F* ^1^, *F* ^2^) is high. Thus, the effect of integrating *F* ^3^ with (*F* ^1^, *F* ^2^) is to strongly attenuate the levels of all species from their saturated state.

We next consider the effect on the basic network of mutually excitatory crosstalk *F* ^4^, depicted by *L*(*F* ^4^∣*F* ^1^, *F* ^2^) in Fig 5. We will focus in particular on the time interval *t* ∈ [20, 40], where *S*_1_ is first activated. For this time interval, the concentrations of *Y*_2_ and *Y*_3_ increase, while the concentrations of *X*_1_–*X*_3_ are transiently reduced. Thus the effect of including mutual excitation is that a non-zero input *S*_1_ is sufficient to activate the second pathway, as well as the first. As in the previous case, we can also analyse the interaction between mutual excitation and the basic network in terms of the mutual dynamics *M*(*F* ^4^; *F* ^1^, *F* ^2^), as depicted in Fig 6. In this time interval, the cooperativity *C*(*F* ^4^; *F* ^1^, *F* ^2^) is briefly negative before returning to zero, while the incompatibility *I*(*F* ^4^; *F* ^1^, *F* ^2^) increases monotonically and converges to around 0.81. Negative cooperativity implies that mutual excitation attenuates the isolated dynamics, although only marginally since *I* is small. This attenuation is also only transient; *C* approaches zero and *I* approaches approximately 0.81 as *t*→40. Thus, by the end of this time interval, integrating mutual excitation creates a significant additional effect in a direction orthogonal to the isolated dynamics. This is intuitively clear, since the incremental effect of mutual excitation is the additive excitation of *Y*_2_ and *Y*_3_, orthogonal to the saturation of *X*_1_–*X*_3_ during *t* ∈ [20, 40].

We have identified the effect of each crosstalk mechanism on the basic network. However, we can see from the plots of *L*(*F* ^3^∣*F* ^1^, *F* ^2^, *F* ^4^) and *L*(*F* ^4^∣*F* ^1^, *F* ^2^, *F* ^3^) that the function of each crosstalk mechanism (when defined as its incremental effect on a network) is very different when the other crosstalk mechanism is present. We can see from the first of these trajectories that one effect of integrating inhibition into the cross-activated network is to effectively insulate the *Y*_1_–*Y*_3_ pathway, since the excitation shown by *L*(*F* ^4^∣*F* ^1^, *F* ^2^) during *t* ∈ [20, 40] is cancelled out by the values of *L*(*F* ^3^∣*F* ^1^, *F* ^2^, *F* ^4^). Furthermore, on comparing *L*(*F* ^3^∣*F* ^1^, *F* ^2^, *F* ^4^) with *L*(*F* ^3^∣*F* ^1^, *F* ^2^) we can see that mutual inhibition has remarkably different incremental effects depending on whether or not mutual excitation is present. This can be quantified by observing the trajectory of

$$\begin{array}{c} M\left(F^{3};F^{4}|F^{1},F^{2}\right)=L\left(F^{3}|F^{1},F^{2}\right)-L\left(F^{3}|F^{1},F^{2},F^{4}\right) \end{array}$$

and the associated *C* and *I* values in the right-hand plots of Fig 6. For *t* ∈ [20, 40], the value of *I*(*F* ^3^; *F* ^4^∣*F* ^1^, *F* ^2^) is large, and *C*(*F* ^3^; *F* ^4^∣*F* ^1^, *F* ^2^) is close to −1. This confirms the assertion above that, in this time interval, the interdependence between crosstalk mechanisms causes them to approximately cancel each other out, so that the behaviour of the entire system is much like that of the isolated pathways. We can observe that, in this time interval, the effect of mutual inhibition on the basic network *L*(*F* ^3^∣*F* ^1^, *F* ^2^) is zero. However, non-zero mutual dynamics *M*(*F* ^3^; *F* ^4^∣*F* ^1^, *F* ^2^) in this time interval means that the presence of mutual excitation causes the inhibition crosstalk to have a non-zero function.

To summarise, we have demonstrated how to use the concepts of conditional dynamics *L*(*F* ^*i*^∣*F* ^1^, *F* ^2^) and mutual dynamics *M*(*F* ^*i*^; *F* ^1^, *F* ^2^) to analyse the effect of each crosstalk mechanism on a pair of isolated pathways. We extended this analysis by using *M*(*F* ^3^; *F* ^4^∣*F* ^1^, *F* ^2^) to quantify how, by integrating the crosstalk mechanisms together, they influence one another’s isolated functions.

### Example 2: Controller

**Fig 7:**
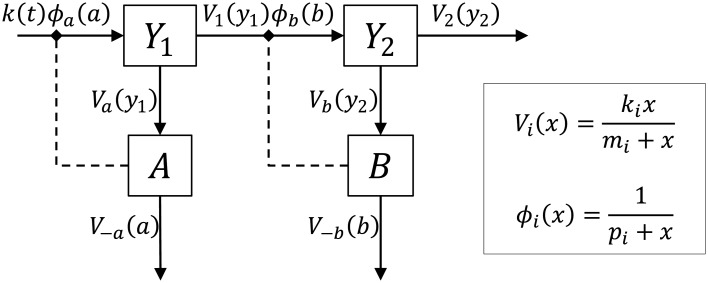
Interacting feedback control for perfect adaptation. A single pathway representing the concentration of two intermediates *Y*_1_ and *Y*_2_, the level of each of which needs to be kept at equilibrium. This system applies two integral feedback controllers, assuming parameters *m*_−*a*_ and *m*_−*b*_ are small relative to concentrations *a* and *b*.

This example illustrates the application of our layered framework to a toy network, comprised of a biomolecular cascade containing two integral feedback motifs (Fig 7). This toy network was adapted from a motif identified in [42] as sufficient to exhibit perfect adaptation to changing external input concentrations, and underlying the ability of the chemotaxis pathway in *E. coli* to show near-perfect adaptation to extracellular chemoattractant concentrations [43–45].

The network consists of two intermediates, *Y*_1_ and *Y*_2_. The species *Y*_1_ is produced with a time-varying rate *k*(*t*) depending on the concentrations of one or more external species. Intermediate *Y*_1_ is irreversibly converted to *Y*_2_ at rate *V*_1_(*y*_1_) and *Y*_2_ is consumed with rate *V*_2_(*y*_2_), each following Michaelis-Menten kinetics (see Fig 7). For certain production rates of *Y*_1_, *V*_1_ is close to saturation so that the concentration of *Y*_1_ eventually becomes unstable for high rates *k*(*t*).

To stabilise the network and achieve perfect adaptation of the concentrations of *Y*_1_ and *Y*_2_ to slowly changing production rates *k*(*t*), the network needs to be controlled. Such controllers can be implemented through additional Michaelis-Menten reactions *V*_*a*_(*y*_1_) and *V*_*b*_(*y*_2_) forming species *A* and *B* from *Y*_1_ and *Y*_2_, respectively. *A* and *B* are consumed with Michaelis-Menten rates *V*_−*a*_(*a*) and *V*_−*b*_(*b*) respectively, both of which are assumed to be close to saturation. The two control loops are closed by non-competitive inhibition of the production of *Y*_1_ and of the conversion of *Y*_1_ to *Y*_2_ modelled by adapting the production rates with the factors *ϕ*_*a*_(*a*) and *ϕ*_*b*_(*b*) respectively, as shown in Fig 7.

The dynamics of the system can be modelled by the following ODEs:

$$\begin{array}{c} \underset{=:\dot{x}}{\underset{︸}{\left[\begin{array}{c} {\dot{y}}_{1}\\ {\dot{y}}_{2}\\ \dot{a}\\ \dot{b} \end{array}\right]}}=\underset{=:S}{\underset{︸}{\left[\begin{array}{ccccccccc} 1 & -1 & 0 & -1 & 0 & 0 & 0 & 0 & 0\\ 0 & 1 & -1 & 0 & 0 & 0 & -1 & 0 & 0\\ 0 & 0 & 0 & 0 & 1 & -1 & 0 & 0 & 0\\ 0 & 0 & 0 & 0 & 0 & 0 & 0 & 1 & -1 \end{array}\right]}}\underset{v(x)}{\underset{︸}{\left[\begin{array}{c} k\left(t\right)\phi_{a}\left(a\right)\\ V_{1}\left(y_{1}\right)\phi_{b}\left(b\right)\\ V_{2}\left(y_{2}\right)\\ V_{a}\left(y_{1}\right)\\ V_{a}\left(y_{1}\right)\\ V_{-a}\left(a\right)\\ V_{b}\left(y_{2}\right)\\ V_{b}\left(y_{2}\right)\\ V_{-b}\left(b\right) \end{array}\right]}}, \end{array}$$

with

$$\begin{array}{c} V_{i}\left(x\right)=\frac{k_{i}x}{m_{i}+x},\;\;\phi_{i}\left(x\right)=\frac{1}{p_{i}+x}. \end{array}$$

Reactions *V*_*a*_ and *V*_*b*_ have two distinct effects on the pathway: first, they decrease the concentration of *Y*_*i*_ independently of any interaction of the controller; and second, they increase the concentration of *A* and *B* and are thus the means by which the controller observes the state of the network. To distinguish these two effects mathematically, in the dynamics above we have distributed each of *V*_*a*_ and *V*_*b*_ into two reactions with the same rates, one only affecting the controller, and the other only the pathway.

**Fig 8:**
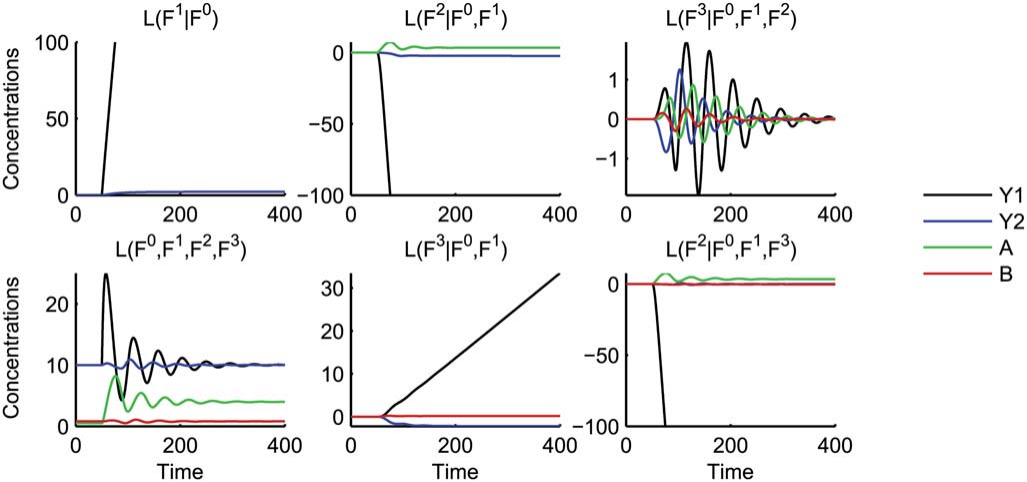
Dynamics of the layers of the perfect adaptation network. The network is equilibrated for a *Y*_1_ production level of *k* = 3. At *t* = 50, the input is changed to *k* = 10. The plots show the dynamics of the layers for two different orderings of the functionalities: *L*(*F* ^1^∣*F* ^0^) are the dynamics of the uncontrolled pathway; *L*(*F* ^2^∣*F* ^0^, *F* ^1^) and *L*(*F* ^3^∣*F* ^0^, *F* ^1^) are the effects of each controller on the unstable pathway; and *L*(*F* ^2^∣*F* ^0^, *F* ^1^, *F* ^3^) and *L*(*F* ^3^∣*F* ^0^, *F* ^1^, *F* ^2^) are the incremental effects of including the other controller given the first. Finally, *L*(*F* ^0^, *F* ^1^, *F* ^2^, *F* ^3^) represents the dynamics of the complete network.

To analyse the system, we define three functionalities, corresponding to the uncontrolled pathway *F* ^1^ (columns 1–4 and 7 in *S*) and the two controllers *F* ^2^ (columns 5 and 6) and *F* ^3^ (columns 8 and 9). The initial condition *x*_0_ was set to represent the steady state concentrations of the species for *k* = 3. Note that, in order to make explicit the dependency of the layered dynamics on this initial condition, we created a ‘zero layer’ *F* ^0^ with constant trajectory *L*(*F* ^0^) = *x*_0_ as described in the Methods section. The ODEs describing the layers’ dynamics for each ordering of the functionalities, the parameter values, as well as the precise initial conditions for this example can be found in the Supporting Information. We then determined the dynamics of the layers when applying a step change of the *Y*_1_ production rate from *k* = 3 to *k* = 10 at *t* = 50. Fig 8 displays the dynamics of the pathway, the conditional dynamics of the controllers given the pathway for both orderings of the controllers, and the complete dynamics of the system.

Similarly to the previous example, we will consider a basic network consisting of only the uncontrolled pathway, *F* ^1^. We are then able to identify the effect of each controller as the conditional dynamics of *F* ^2^ and *F* ^3^ given the basic network. However, the effect on the basic network of both controllers will be different to the sum of each controller’s individual effect. We will thus compute the mutual dynamics between the two controllers in order to analyse how the controllers interact with one another when both are integrated in the context of the basic network.

We first consider the isolated dynamics *L*(*F* ^1^∣*F* ^0^) of the uncontrolled pathway which makes up the basic network, depicted in Fig 8. After the step increase at *t* = 50, the two reactions decreasing *Y*_1_ quickly saturate. The isolated system is therefore unstable, and the *Y*_1_ component of *L*(*F* ^1^∣*F* ^0^) increases to infinity at a positive constant slope. We will now use this basic network to define the function of each of the controllers.

**Fig 9:**
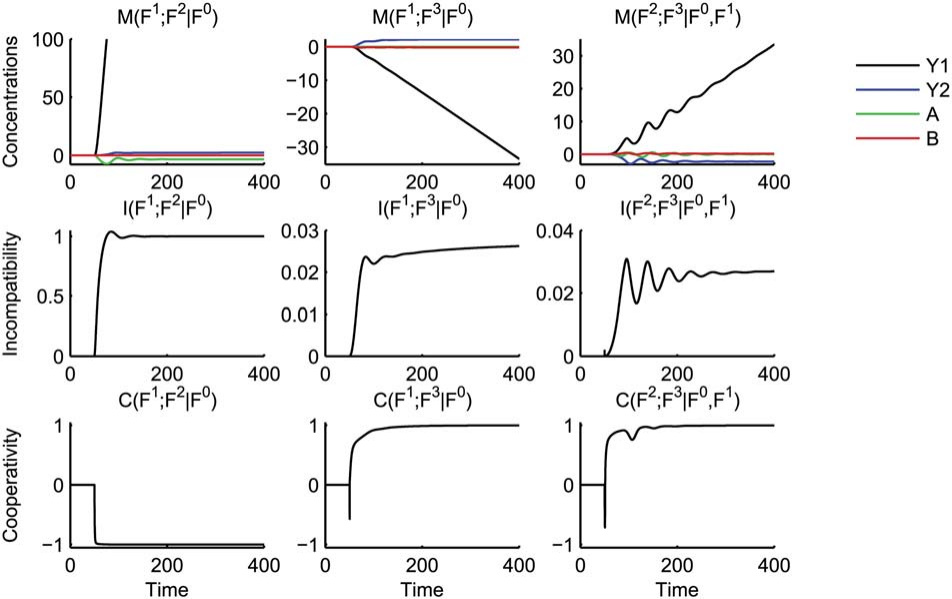
Mutual dynamics, incompatibilities and cooperativities between the functionalities of the perfect adaptation network. The first column shows the interaction of the pathway (*F* ^1^) with the first controller (*F* ^2^), the second column the interaction of the pathway with the second controller (*F* ^3^), and the third column the interaction of the two controllers given the pathway.

Integrating the controller *F* ^2^ with the pathway stabilises the network. This is indicated by the trajectory of *L*(*F* ^2^∣*F* ^0^, *F* ^1^) in Fig 8. In particular, the *Y*_1_ component of this trajectory decreases to minus infinity with approximately −1 times the rate of increase of *Y*_1_ in the isolated pathway. Another way to observe this stabilising effect is to consider the mutual dynamics *M*(*F* ^1^; *F* ^2^) between the pathway and the controller, shown in Fig 9. The inconsistency *I*(*F* ^1^; *F* ^2^∣*F* ^0^) between pathway and controller approaches one, while the cooperativity *C*(*F* ^1^; *F* ^2^∣*F* ^0^) minus one. We interpret these values as the two functionalities cancelling one another out; in other words, *F* ^2^ stabilising *F* ^1^.

We next consider the effect of the second controller *F* ^3^, depicted in Fig 8 by *L*(*F* ^3^∣*F* ^0^, *F* ^1^). The *Y*_1_ component of *L*(*F* ^3^∣*F* ^0^, *F* ^1^) also diverges to positive infinity, albeit at a much slower rate than that of the basic network. Intuitively, the second controller cannot stabilise the basic network, since it has no means to observe the unstable *Y*_1_ component. Instead, its interventions further destabise the pathway. We can quantify this through the mutual dynamics between the controller and pathway. Indeed, since *C*(*F* ^1^; *F* ^3^∣*F* ^0^) ≈ 1, the second controller amplifies the unstable dynamics. However, since *I*(*F* ^1^; *F* ^3^∣*F* ^0^) ≪ 1, this effect is only minimal.

We now consider the interaction between the two controllers when combined with the uncontrolled pathway. Interestingly, the effect of the second controller when combined with the first is only transient, as shown by *L*(*F* ^3^∣*F* ^0^, *F* ^1^, *F* ^2^) in Fig 8. However, the mutual dynamics *M*(*F* ^2^; *F* ^3^∣*F* ^0^, *F* ^1^) between the two controllers have a *Y*_1_ component increasing slowly to infinity, and the cooperativity between the controllers *C*(*F* ^2^; *F* ^3^∣*F* ^0^, *F* ^1^) approaches 1. This positive cooperativity indicates that the first controller increases its interventions to stabilise the network when the second controller is present.

That the two controllers given the pathway act cooperatively might initially appear surprising, since the first controller stabilises the network while the second destabilises the network. This indicates the utility of our notation in allowing a rigorous quantification of how functionalities interact, given their environment. Consider the following three cases for how the two controllers *F* ^2^ and *F* ^3^ interact. First consider the interaction *M*(*F* ^2^; *F* ^3^∣*F* ^0^, *F* ^1^) between the two controllers when integrated with the pathway. Since the presence of *F* ^3^ increases the control action by *F* ^2^ necessary to stabilise the pathway, the cooperativity given the pathway *C*(*F* ^2^; *F* ^3^∣*F* ^0^, *F* ^1^) approaches 1, as described above. Next, consider the interaction *M*(*F* ^2^; *F* ^3^∣*F* ^0^) between the two controllers *without* the pathway. They cannot influence each other since the respective subnetworks are not connected. Consequently they do not interact and hence *M*(*F* ^2^; *F* ^3^∣*F* ^0^) = *C*(*F* ^2^; *F* ^3^∣*F* ^0^) = *I*(*F* ^2^; *F* ^3^∣*F* ^0^) = 0. Finally, consider the interaction *M*(*F* ^2^; (*F* ^1^, *F* ^3^)∣*F* ^0^) between the first controller *F* ^2^ and the unstable network comprised of the pathway *F* ^1^ extended with *F* ^3^. The first controller stabilises the extended pathway, which is represented by the cooperativity *C*(*F* ^2^; (*F* ^1^, *F* ^3^)∣*F* ^0^) approaching minus one, as *F* ^2^ acts to attenuate the instability of *F* ^1^, *F* ^3^. Thus, we have demonstrated that the rigorous definition of interdependence using our cascaded layering framework allows us to identify which components of a network amplify or attenuate each other, by defining the components which interact and the context in which they do so.

### Example 3: Metabolic Fluxes in Glycolysis

Glycolysis is a central ten-step process in most organisms responsible for the production of energy in form of ATP and NADH by catabolism of glucose and other sugars (see [46], p. 88 ff.). Besides being one of the best studied metabolic pathways, it is also an important starting point for biotechnological processes [47]. For non-growing *S. cerevisiae* cells, a kinetic model of the glycolytic pathway was created by Hynne *et al*. [48], including fermentation, glycerol production, lactonitrile and glycogen formation, and cellular import and export processes for glucose and other metabolites. We will use this model (available at the BioModels Database [41], model 61) to exemplify how to apply our layering approach in the context of metabolic engineering.

In this section we use Elementary Flux Modes (EFMs) [13], minimal functional pathways which can carry non-zero fluxes at steady state and which fulfil positivity constraints for irreversible reactions. From the unique set of EFMs of a network, all steady-state flux distributions can be obtained by non-negative linear combinations of EFMs. Due to their simplicity, EFMs can often be associated with certain elementary ‘tasks’ of a network, like the production of one or more final products from various available extracellular substrates. Thus EFMs are natural candidates to represent functionalities.

The model in [48] includes the dynamics of the cofactors *NAD* ^+^, *NADH*, *AMP*, *ADP*, and *ATP*, with the consumption of *ATP* by the rest of the cell modelled by first order mass action kinetics. The cofactor concentrations can be interpreted as control inputs to the junctions of the glycolytic pathway dynamically channelling the metabolite flux into the different branches depending on cellular requirements, e.g. during hyperosmotic conditions [49]. The feedback loop is closed by an integral controller ‘observing’ the consumption and production of the cofactors by the glycolytic pathway and other cellular processes.

**Fig 10:**
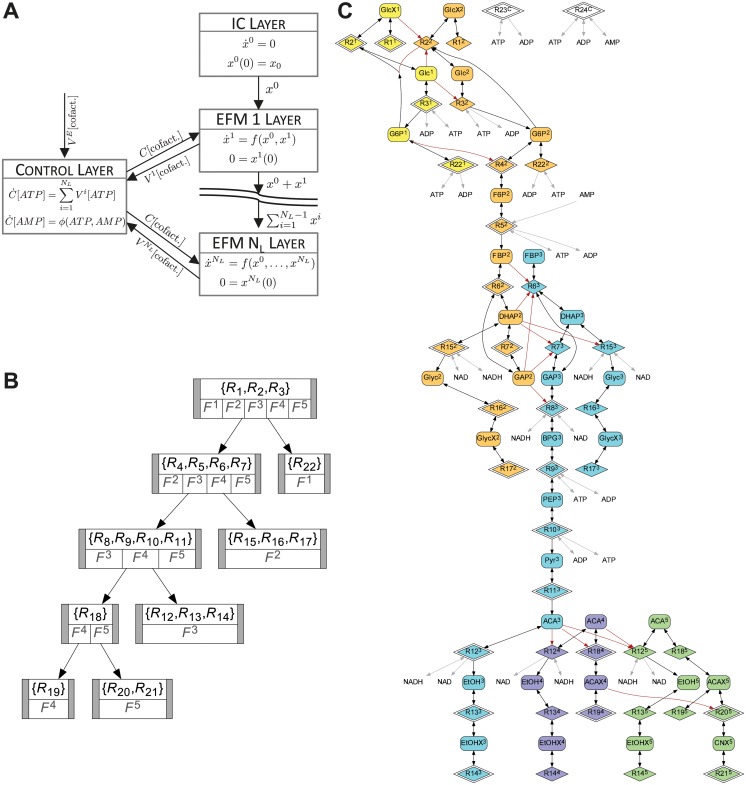
Layering of the glycolytic pathway. A) Mixed layer structure (simplified). Each EFM corresponds to a functionality in the cascaded layer structure. The control layer communicates with all other layers by sending to each the current cofactor concentrations *C*[cofact.], and by receiving the cofactor production/consumption rate *V* ^*i*^[cofact.], *i* ∈ 1, …, *N* ^*L*^, of each EFM, but also of other cellular processes (*V* ^*E*^[cofact.]). B) Graph representing the common reactions of the EFMs. Each note represents a set of reactions shared by the indicated EFMs. The binary tree structure of this graph is a special property of the glycolytic model [48]. C) Species-reaction graph (compare [18]) representing the models of the cascaded layers as well as the inter-layer information transfer. Rounded rectangles represent metabolite concentrations, diamonds reactions. An arrow is drawn from a metabolite to a reaction if the reaction rate depends on the metabolite, and an arrow from a reaction to a metabolite if firing of the reaction changes the metabolite’s concentration. Colours and super-scripts indicate the layer to which species and reactions belong. Reactions with only one border correspond to altered reactions. Black arrows are dependencies inside the model of the respective layer, red arrows indicate information transfer between layers. Grey arrows represent links to cofactors in the control layer; reactions *R*23 and *R*24 are part of the control layer. Graphs (B) and (C) drawn with Graphviz [57].

We established a mixed layer structure for the glycolysis model (Fig 10). This mixed layer structure consists of the EFMs (not taking into account mass balance of the cofactors) as functionalities in a cascaded layer structure. The dynamics of cofactors, together with ATP consumption (*ATP*→*ADP*, Reaction 23 in [48]) and the AK reaction (*ATP* + *AMP* ↔ 2*ADP*, Reaction 24) form a control layer that communicates with all layers in the cascade without being part of the cascade.

Using the software efmtool [50], we identified eight EFMs for the modified network where the cofactor dynamics were removed by setting the appropriate rows in the stoichiometric matrix to zero. Three of the identified EFMs were non-negative linear combinations of other EFMs, a consequence of treating each reversible reaction as two separate irreversible reactions. Based on the concept of simplicity (condition *C3* in [13]), we removed the linearly-dependent EFMs with the lowest number of zero entries. The remaining five EFMs can be interpreted as representing elementary ‘tasks’ of the network:

1. Glycogen buildup: production and storage of glucose-6-phospate *G*6*P*
2. Production and excretion of glycerol *Glyc*
3. Fermentation: production and excretion of ethanol *EtOH*
4. Production and excretion of acetaldehyde *ACA*
5. Lactonitrile *lacto* formation.

Since the cofactors participate in many of the reactions, our approach to not take them into account when calculating the EFMs is conceptually similar to classifying metabolites taking part in more than a threshold number of reactions as ‘external’, as proposed in [51]. Both approaches result in the same significantly simpler and biologically interpretable set of EFMs. However, unlike the subnetworks identified in [48], the EFMs are not necessarily redox neutral. We will briefly discuss the consequences of this at the end of this section.

In Fig 10 we represent the common reactions of the five EFMs by a graph. Surprisingly, the graph is a binary tree, indicating that, while there are junctions in the reaction pathway of the metabolites, there are no joins. Furthermore, there exists one main branch, consisting of Reactions 1 − 11 and Reaction 18, and each EFM can be represented by a unique junction from this main branch: EFM *F* ^1^ is the junction after Reaction 3, *F* ^2^ after Reactions 6 and 7, *F* ^3^ after Reaction 11, and *F* ^4^ and *F* ^5^ after Reaction 18. This property provides a natural order for the EFMs, with *F* ^1^ at the top of the cascade and *F* ^5^ at the bottom. The ODEs describing the layers’ dynamics for this ordering of the functionalities, the dynamics of the control layer, the parameter values, and the initial conditions can be found in the Supporting Information.

The layers and their interconnection can be represented as a species-reaction graph (SR graph, see [18]) depicting the reactions, the non-zero altered reactions, the species with non-zero dynamics in each layer, and the inter-layer communication (Fig 10). Recall from (7b) that the vector of altered reactions for layer *i* is defined by

$$\begin{array}{c} v_{alt}\left(x^{1}+\cdots +x^{i-1},x^{i}\right)≔v\left(x^{1}+\cdots +x^{i}\right)-v\left(x^{1}+\cdots +x^{i-1}\right). \end{array}$$

In this example we identified reactions *j* where *v*_*j*_(*x* ^1^ + … + *x* ^*i*^) = *v*_*j*_(*x* ^1^ + … + *x* ^*i*−1^), meaning the rate of the reaction is not altered by the interconnection of functionality *F* ^*i*^. A sufficient condition [18] to conclude that altered reaction *j* does not change the dynamics of Layer *i* and thus can be omitted is if the *j*th element of

$$\begin{array}{c} I(\frac{\delta }{\delta x}v)\exp(I(\sum_{k=1}^{i-1}S^{k})I(\frac{\delta }{\delta x}v))\cdot I\left(S^{i}\right)\cdot 1 \end{array}$$ (13)

is zero. Here, exp(.) represents the matrix exponent, *S* ^*k*^ the stoichiometric matrix of layer *k*, *I* the element-wise indicator function being one if the respective element is non-zero and zero otherwise, and **1** the *N*_*R*_ × 1 vector with all elements being one. Similarly, the integration of a species can be omitted in a layer if it is not affected by any of that layer’s reactions.

Fig 10 shows that each layer is comparatively small and includes few altered reactions. Furthermore, since reactions *R*_13_ and *R*_14_ are linear (in the metabolites), information about the metabolite concentrations involved in these reactions is not required to be transmitted between the layers (for example, *EtOH* ^3^ and *EtOHX* ^3^ are not transmitted to Layer 4 and 5). This demonstrates that, for many biological networks, layers and the interfaces between them are comparatively simple.

We equilibrated the model for a mixed flow glucose concentration of 10 mM and initialised the dynamics of the initial condition layer *L*(*F* ^0^) = *x*_0_ to these steady-state values. This corresponds to low glucose conditions in the range of the *K*_*m*_ values of high-affinity glucose transporters [52], and slightly below the mixed flow glucose concentration for which the parameters of the model were identified (18.5 mM) to prevent glycogenic oscillations (see [48]). All other parameters and medium conditions were kept unchanged as compared to the version of the glycolysis model [48] available at the BioModels Database. We then calculated the layer dynamics for each ordering of the EFMs into all possible cascade structures by simulating the layering graph for 1000 min as described in Methods. This requires the simulation of 2^5^ − 1 = 31 layers to populate the 5 ⋅ 5! = 600 nodes of the graph.

**Fig 11:**
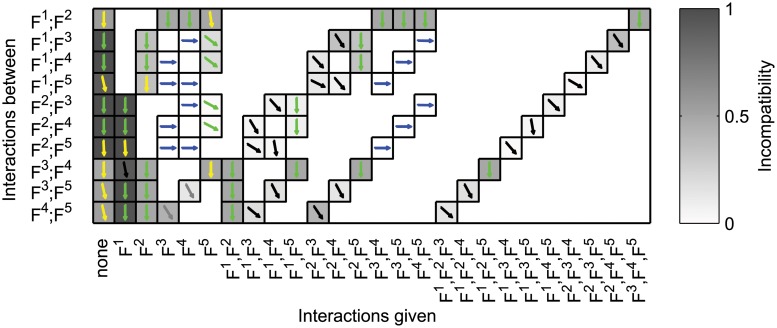
Compatibility of EFMs in the glycolytic pathway. The background shade of the matrix elements depict the steady-state incompatibility between the respective functionalities on the y-axis, given the functionalities on the x-axis, at a mixed flow glucose concentration of 10 mM (for notational convenience, dependencies on *F* ^0^ were assumed). For example, the shade of the element on the second row and twelfth column depicts *I*(*F* ^1^; *F* ^3^∣*F* ^2^, *F* ^4^). The direction of the arrows depict the steady-state cooperativity between the respective elements, with straight upwards corresponding to cooperativity equal one, straight downwards equal minus one, and horizontal equal zero. Finally, the colour of the arrows provides further information about the interaction: (black) the sum of both conditioned functionalities alone as well as the mutual dynamics are stable, and the overall network is stable; (grey) same as black, only the overall network is unstable. Thus, the instability is in a layer the two functionalities are conditioned on; (green) the sum and the mutual dynamics are unstable and the overall network stable. The interaction stabilises the network; (yellow) same as green, but the overall network is unstable. Typical when the interaction only stabilises some of the species, or if the speed of divergence is smaller; (blue) the sum is unstable and the mutual dynamics and the overall network are stable. Typical if the instability was in a layer the functionalities are conditioned on, and one of the functionalities stabilises the network.

Based on the layering graph we then analysed all pairwise interactions between *F* ^*i*^ and *F* ^*j*^, given all possible combinations of other functionalities. In Fig 11, we display the steady-state incompatibilities and cooperativities between the EFMs after convergence at the end of the simulation. Note that although the incompatibilities and cooperativities converge, this is not necessarily true for the corresponding layer and mutual dynamics, which diverge for some EFMs (compare Fig 9). Several pairs have an incompatibility close to one and a cooperativity close to minus one. This combination is typical if one of the functionalities (given its environment) is unstable but is stabilised by the other. For example, in the isolated layer *L*(*F* ^3^∣*F* ^0^), pyruvate increases to infinity since the PDC reaction (Reaction 11 in [48]) is saturated and becomes rate limiting. Integrating either *F* ^1^ or *F* ^2^ with *F* ^3^ reduces the production rate of pyruvate and thus stabilises the network, since both *L*(*F* ^1^, *F* ^3^∣*F* ^0^) and *L*(*F* ^2^, *F* ^3^∣*F* ^0^) are stable. On the other hand, in the isolated layer *L*(*F* ^5^∣*F* ^0^) both pyruvate and acetaldehyde (*ACA*) concentrations are unstable, the latter due to limited cyanide (*CNX*) availability. Integrating *F* ^1^ or *F* ^2^ with *F* ^5^ only stabilises the pyruvate concentration, but not acetaldehyde. The prediction of infinite growth of species concentrations is unlikely to be observed experimentally. Instead, the instability might indicate that the conversion of pyruvate to acetaldehyde catalysed by the pyruvate decarboxylase (pdc) becomes a rate limiting step when synthetically increasing the flow through the respective EFM. Indeed, it was experimentally validated [53] that under certain conditions over-expression of pdc after reducing glycerol synthesis can lead to increased growth rates and ethanol yield.

**Fig 12:**
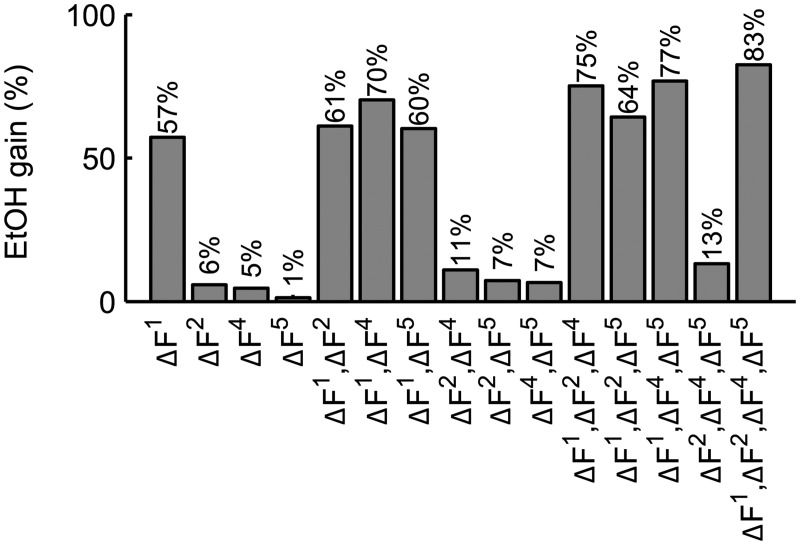
Potential ethanol production gains by knock-outs of functionalities. The bars represent the potential gain in ethanol production for a mixed flow glucose concentration of 10 mM if one or more of the EFMs are knocked out, e.g. by gene deletions of corresponding enzymes. These values can be directly obtained from the respective steady-state extracellular ethanol concentrations in the layers in the layering graph. For example, the expected gain for knocking out functionalities *F* ^1^ and *F* ^4^ corresponds to −*L*_*EtOHX*_(*F* ^1^, *F* ^4^∣*F* ^0^, *F* ^2^, *F* ^3^, *F* ^5^)/*L*_*EtOHX*_(*F* ^0^) as *t* → ∞. Note that for some knock-outs the resulting networks become unstable (see main text), although not in the concentration of ethanol.

The incompatibilities between two EFMs, given the three other EFMs, are of specific interest (rightmost ten interactions in Fig 11). They indicate that *F* ^1^ and *F* ^3^ are significantly more incompatible than *F* ^2^ and *F* ^3^. Recall that *F* ^3^ corresponds to the fermentation capability of the network. To increase biofuel production one would intuitively expect that the highest yields could be obtained by knocking out or down the EFMs which are most incompatible to *F* ^3^, thereby removing EFMs which act to attenuate its function. Recall that the effect of knocking-out a certain functionality *F* ^*i*^ on the overall dynamics of the network is equal to −*L*(*F* ^*i*^∣*F* ^*j*^, *j* ≠ *i*). Thus, the effect of knocking out *GPD* (Reaction 15) and, thus, glycerol production *F* ^2^ is −*L*(*F* ^2^∣*F* ^0^, *F* ^1^, *F* ^3^, *F* ^4^, *F* ^5^). The effect of a double knock-out of glycerol production together with glycogen build-up *F* ^1^ corresponds to −*L*(*F* ^1^, *F* ^2^∣*F* ^0^, *F* ^3^, *F* ^4^, *F* ^5^). The theoretical effect on fermentation efficiency of all possible combinations of knock-outs (Fig 12) can thus be directly derived, given the layering graph. Indeed, this analysis confirms that fermentation *F* ^3^ is significantly more incompatible with glycogen build-up *F* ^1^ than with glycerol production *F* ^2^ at the given experimental setup. The effect of knocking out more than one functionality is non-linear, and a double knock-out of the two functionalities yielding the highest gains when knocked out separately is not necessarily optimal, as shown in Fig 12. Thus, the layering graph provides an effective tool to analyse such potential interactions.

It is out of the scope of this article to take side effects of the proposed genetic modifications into account, such as possible viability issues when knocking out some of the pathways. Our intention was to provide a proof of principle of how to apply our layering framework in the context of metabolic engineering. Notably, our analysis is based on a model of a non-industrial strain of *S. cerevisia* cells grown under glucose starvation, and which was created mainly to explain glycogenic oscillations at low glucose conditions [48] rather than being optimised for metabolic engineering purposes. Furthermore, we essentially avoided the challenge of balancing the redox state (*NAD* ^+^/*NADH* ratio) by our mixed layering approach (Fig 10) in which the control layer was always receiving the cofactor consumption and production rates of all cascaded layers while simulating the layering graph. Nevertheless, our analysis indicates that ethanol yield can be increased by approximately 6% by knocking out glycerol formation (*F* ^2^). This prediction is in good agreement with a reported [54] experimental increase of about 7% after inhibition of glycerol formation from dihydroxyacetone phosphate (*DHAP*) by knock-down of glycerol-3-phosphate dehydrogenase (*GPD*). In [54] the redox state was balanced by synthetically engineering an alternative route in the endogeneous Embden–Meyerhof–Parnas pathway based on *NADP* ^+^ and *NADPH* instead of *NAD* ^+^ and *NADH*.

## Discussion

We have described a cascaded layering approach which can be used to systematically identify the interactions of functionalities (i.e. functional subsystems) in a decomposed network. Functionalities are defined as sets of reactions which together accomplish a given purpose. We have identified the dynamics of an isolated functionality as the dynamics of the subnetwork made up of only those reactions. However, the dynamics of a functionality are different when it is in the context of others. We have formulated the conditional dynamics of a functionality as the incremental change to the dynamics caused by integrating it with its context. We have also demonstrated that the computational burden associated with this layered framework can be minimised by exploiting symmetries within the definitions of interdependence.

We have used the conditional dynamics to define the mutual dynamics, which describe the context-dependent interaction between any pair of functionalities. We can thus identify if the interaction between them is strong or weak (*incompatibility*), and to what extent they amplify or attenuate each other (*cooperativity*). This interdependence is also context-dependent, so that two functionalities may interact in vastly different ways depending on the wider system in which they are integrated. Our framework allows the unambiguous quantification of nonlinear interactions between functionalities.

Finally, we illustrated our layering framework with three examples. The first considered signalling pathways interconnected by two distinct crosstalk mechanisms, a mutually inhibiting and a mutually excitatory crosstalk. We not only quantified how each mechanism separately influences the dynamics, but also how the crosstalk mechanisms interact with each other. Our second example concerned a pathway stabilised by two integral feedback mechanisms. For certain input signals, the pathway alone becomes unstable. It gets stabilised by the first integral feedback, but the second feedback further destabilises the system, as displayed by the negative and positive cooperativity respectively. Thus each feedback has an opposite effect on the pathway to the other when each is considered in isolation. However, when they are integrated together they interact cooperatively, due to the first controller increasing its strength to stabilise the network when the second controller is present. Third, we showed how to apply our layering framework in the context of metabolic engineering to analyse the dynamic interactions between elementary flux modes in glycolysis. Interestingly, the effect of abolishing an EFM by knocking out its enzymes can be easily described as the negative of the conditional dynamics of that EFM, given the rest of the network. The incompatibility between two EFMs, on the other hand, gives information about the expected increase in the flux through one EFM if the other one is knocked out. These interpretations allowed us to efficiently calculate the expected increase of ethanol yield in biofuel production when knocking out arbitrary combinations of EFMs. Interestingly, this yield is not additive, and a double knock-out of the two EFMs giving the highest increases in ethanol yield alone is not necessarily optimal. Instead, our method to calculate the integrated effect of all combinations of knock-outs allowed us to assess the best metabolic engineering strategy with a minimum amount of simulations required.

As discussed in the Introduction, the layering framework [16, 17] differs from modularization approaches [18–26]. Each of these frameworks allows different network architectures to be considered, reflecting a distinction between vertical and horizontal network decomposition [55]. Layered networks represent the overlay of multiple (possibly competing) functional subnetworks, while the modular framework interprets biological networks in terms of engineered interconnections of input–output systems. Although it is a modular phenomenon, previous work on quantifying retroactivity [4, 5] can be related to our layered formulation. A goal of both techniques is to measure the difference in a given subsystem’s isolated and integrated behaviours. In particular, an upper bound on the Euclidean norm of the difference in a module’s isolated and integrated trajectories has been derived in terms of the system parameters in [5]. In the Supporting Information to this paper, we show in detail how this norm relates to our formulation of the incremental dynamics *L*(*F* ^2^∣*F* ^1^), and the mutual dynamics *M*(*F* ^1^, *F* ^2^). Although our layering framework and the concept of retroactivity were developed to quantify the interactions between different kinds of subsystems, this comparison suggests that our additional measures of incompatibility and cooperativity as defining an interaction *direction* may also be applicable to further understand retroactivity in multi-dimensional modular systems.

The computational method employed to quantify the interaction strength between a given functionality pair is clearly dependent on the kinetic constants and other parameter values in the model, including the initial conditions. A repetition of this computation across a range of such values may give a more informative picture of how uncertain biological functionalities interact, but will be difficult to visualise and expensive to evaluate. Possible directions for future work may include the derivation of analytical estimates in terms of the system’s parameter values for the mutual dynamics, incompatibility, or cooperativity, of the type produced for layered steady state perturbations in [16] or retroactive perturbations in [5]. It would also be interesting to consider if any estimates of our measures can be computationally derived to hold for all parameters, similarly to the structural results on the direction of steady state responses to parameter perturbations given in [34]. Alternatively, semi-definite programming may be used to calculate worst-case estimates of the difference between the trajectories *L*(*F* ^1^) and *L*(*F* ^1^∣*F* ^2^) without resorting to simulation, similarly to previous work on model reduction error estimation [56].

An important assumption made in this paper was that the network functionalities *F* ^*i*^ are given. The examples in this paper provide two different strategies for how the functionalities necessary for our layering framework can be defined. In the first two examples this was done by intuition and prior knowledge, while in the third example we applied an already available method to group functionally related reactions in metabolic pathways, namely EFMs. Clearly, this is not a comprehensive list of how functionalities can be defined, and (depending on the precise application) other strategies might be more promising. One such example may arise in the context of Synthetic Biology, from the specification of synthetic biomolecular devices such as toggle switches, oscillators, and so on, the dynamics of which are significantly affected when combined into large-scale systems [2]. Such an application of our framework to Synthetic Biology would require these biomolecular devices–typically designed and modelled as modules–to be mathematically expressed as layers, respectively functionalities. If this is possible without requiring modifications in the biomolecular implementation remains a question for future research.

Our layering approach provides a general and concise framework for the quantification of the nonlinear interactions between functionalities of signalling and metabolic pathways. It offers a great flexibility since it only requires that each reaction must be part of a functionality, but allows reactions to be part of more or even all functionalities. Besides the mathematical definition of the model, the only input data needed is the problem-specific definition of the sets of reactions which make up a functionality. For many signalling pathways these definitions are typically given by biological insight, and for metabolic networks implementations of efficient algorithms to determine the sets of EFMs are available (e.g. [50]). To allow a quick assessment of our approach, we have implemented a MATLAB (The MathWorks, Natick, MA) toolbox, available under the GNU General Public License from sysos.eng.ox.ac.uk/control/sysos/index.php/User:Prescott/Code. This toolbox provides algorithms to automatically construct the models of the layers from SBML files, to derive the layering graph with minimal numerical simulation, and to easily assess the mutual dynamics, the incompatibility and the cooperativity between functionalities. We hope that our implementation can serve as a starting point to integrate our layering framework into standard software solutions for the analysis of biomolecular networks.

## Supporting Information

S1 Text Mathematical details for the example networks, and relationship to retroactivity. (PDF)
