# Supplementary material for: Quantification of Interactions between Dynamic Cellular Network Functionalities by Cascaded Layering
Source: PLoS Comput Biol. 2015 May 1;11(5):e1004235. doi: 10.1371/journal.pcbi.1004235 (PMC4416712; doi:10.1371/journal.pcbi.1004235)
Supplement: S1 Text — (PDF) [file pcbi.1004235.s001.pdf]

Quantification of Interactions Between Dynamic Cellular Network Functionalities by Cascaded Layering

Thomas Prescott[1,2†], Moritz Lang[3,†], Antonis Papachristodoulou[1,*]
1 Department of Engineering Science, University of Oxford, Parks Road, Oxford, OX1 3QU, United Kingdom
2 Life Sciences Interface Doctoral Training Centre, University of Oxford, Parks Road, Oxford, OX1 3PJ, United Kingdom
3 Department of Biosystems Science and Engineering, ETH Zürich, and Swiss Institute of Bioinformatics, Basel, Switzerland
† Contributed equally
∗ E-mail: antonis@eng.ox.ac.uk

Layering and Modularity: Relationship to Retroactivity

In this section we compare the layered and modular decompositions of a biomolecular network in more mathematical detail. This is with particular reference to the relationship between the concept of retroactivity and the interaction framework introduced in the paper.

As noted in the main text, the intention of the layering framework is to describe and analyse 'vertical' network decompositions, whereas most modularization methods aim to describe 'horizontal' decompositions [1]. This distinction is reflected by our definition of functionalities as possibly overlapping sets of reactions, where the dynamics of all species may be contributed to by all layers (for more details, see [2,3]). In contrast, in most modularization approaches, each species and each reaction of the network belongs to exactly one module (with the exception of [4]). To further illustrate these distinctions, we can consider to what extent we can express modular phenomena in the layered terms introduced in this paper. In this SI, the particular phenomenon we focus on is *retroactivity.*

Following the definition of modular decomposition in e.g. [5,6], let a module M^i be defined by the pair $M^i = (X^i, F^i)$ of subsets of species X^i, and reactions F^i, for $i = 1, \dots, N_M$ for a given number of modules N_M. It is assumed that $\{X^i\}$ partitions the species set, and similarly that $\{F^i\}$ partitions the reaction set, so that $X^i \cap X^j = \emptyset = F^i \cap F^j$ for $i \neq j$. Furthermore, let the map P^i be defined such that it selects only the module i species (i.e. those belonging to X^i), to define the module's concentration vector $x^i = P^i x$ from the network concentration vector x. In the terminology of this paper, the trajectory of module M^i in the system where modules $M^1, \dots, M^{N_M}$ are all integrated together is equal to $P^i L(F^1, \dots, F^{N_M})$.

Suppose first that $N_M = 2$. A simple type of retroactivity that can be observed between a network of two modules M^1 and M^2 is where M^2 is 'downstream' of M^1, in the sense that species in X^1 affect the dynamics of M^2 either through information flow (i.e. affecting the rates of reactions in F^2 without being produced or consumed) or mass flow (i.e. actively taking part in reactions in F^2). Here, mass flows may cause retroactive effects [7]. It can be useful [8] to quantify the change $(\Delta x^1)_{M^2}$ of the dynamics of M^1 caused by loading it with M^2, which can be written as

$$(\Delta x^1)_{M^2} = P^1[L(F^1, F^2) - L(F^1)] = P^1 L(F^2|F^1),$$

where subscript M^2 is intended to clarify that M^1 is loaded with M^2. This accords with our intuition, since we interpret $L(F^2|F^1)$ as the incremental effect (on the entire state space) of integrating the F^2 reactions with those in F^1. Pre-multiplying by P^1 simply projects the trajectory onto the states in X^1 of module M^1. Note that, in the layered formulation, the reactions in F^1 do not need to be simulated again; the retroactive effect on X^1 is instead simulated by the downstream layer $L(F^2|F^1)$. As well as giving the retroactivity, this trajectory can also be pre-multiplied by P^2 to give the downstream dynamics $P^2 L(F^2|F^1)$ of module M^2 driven by M^1.

In [8] an estimate of the magnitude $\|(\Delta x^1)_{M^2}\| = \|P^1 L(F^2|F^1)\|$ is derived. We can directly compare this measure to a version of incompatibility $\hat{I}(F^1; F^2) = \|M(F^1; F^2)\|_{P^1} = \|L(F^2|F^1) - L(F^2)\|_{P^1}$. We

have adapted that measure such that (a) this magnitude is not normalised, as in the definition of I, and (b) the norm is weighted such that $\|X\|_{P^1} = \|P^1 X\|_2$, in order to consider only the subset X^1 of species corresponding to M^1. Clearly, the main difference between these quantities is the term $P^1 L(F^2)$, that represents the dynamics of the species in X^1 caused by the reactions in M^2 when considered in isolation. The effect of subtracting these dynamics in our measure is that the resulting symmetric definition of the mutual dynamics M represents the behaviour of the combined layers (or, in this case, modules) that cannot be explained by the linear combination of the dynamics of each of the two modules in isolation. Thus, rather than measuring the load put on an upstream module, we measure the nonlinearity arising from combining the two layers.

We can further illustrate how mutual dynamics gives additional insight into retroactive networks by observing how retroactive effects combine. For example, suppose an additional module M^3 is also 'downstream' of M^1. Its effect $(\Delta x^1)_{M^3}$ on the isolated module M^1 is equal to $P^1 L(F^3|F^1)$ by exactly the same reasoning as above. However, the combined effect of the two loads on the isolated layer is equal to

$$\begin{aligned}(\Delta x^1)_{M^2,M^3} &= P^1[L(F^1,F^2,F^3) - L(F^1)] = P^1[L(F^2|F^1) + L(F^3|F^1) - M(F^2;F^3|F^1)]\\ &= (\Delta x^1)_{M^2} + (\Delta x^1)_{M^3} - P^1 M(F^2;F^3|F^1).\end{aligned}$$

Thus the two retroactive effects of each of M^2 and M^3 on M^1 are not necessarily additive; the projected mutual dynamics $P^1 M(F^2;F^3|F^1)$ represent the nonlinear retroactive effects on M^1 of having *both* downstream modules present. Furthermore, by pre-multiplying the square-bracketed terms by other maps P^2 and P^3 we can also reason about the effect of these nonlinear interactions on the indirect influences between modules M^2 and M^3. Of course, when layering a network the pre-multiplication is not necessary; the quantity $M(F^2;F^3|F^1)$ encodes all of the information of how the two layers combine to change their dynamics from their linear combination $L(F^2|F^1) + L(F^3|F^1)$. The difference between the retroactivity measure in [8], and the mutual dynamics M, incompatibility I, and cooperativity C reflect the different interpretations of subsystem interactions in modular and layered decompositions.

This short comparison was intended to use the well-known concept of retroactivity to illustrate certain similarities and differences between the layering and modular frameworks. Of course, while some effects associated with retroactivity can be expressed using our layering framework, [8] is part of an extensive body of work concerned with modular interactions that we have not compared in as much detail. Nevertheless, the modular approach assumes an *a priori* direction to a signal between two modules, the back-acting retroactivity of which can be quantified. However, our layered framework rests on the observation that, if reactions with a common species are assigned to multiple modules, it may not be intuitively obvious which of those modules the common species should be assigned to, or indeed which module is upstream of the other. Our solution to this problem was the layered framework in [2,3] that defined subsystems only by their reaction sets, and which was developed further here to also allow reactions to take part in multiple layers. This means that, rather than the modular interpretation of 'signal propagation with retroactivity', the layered approach instead considers the nonlinearities that are incurred by the overlaid contributions of each layer.

Models for Example 1

General Setup

Parameters:

The strength of the mutually excitatory crosstalk was set to $k_a = 0.1$, and the strength of the mutually inhibitory crosstalk to $k_d = 1$. All other parameters were kept unmodified as compared to crosstalk model [9] available at the BioModels Database [10], Model 116. We refer to [9] for more information

about the interpretation of the parameters.

$$
\begin{aligned}
X_{1T} &= 10 & X_{2T} &= 10 & X_{3T} &= 10\\
Y_{1T} &= 10 & Y_{2T} &= 10 & Y_{3T} &= 10\\
k_x &= 1 & K_{mx} &= 1 & k_{12x} &= 1\\
k_{23x} &= 1 & k_d &= 1 & k_a &= 0.1\\
K_{myx} &= 1 & k_y &= 1 & K_{my} &= 1\\
k_{12y} &= 1 & k_{23y} &= 1 & K_{mxy} &= 1\\
C &= 1
\end{aligned}
$$

Initial Conditions/ "Zero Layer Dynamics" $L(F^0)$:

All states of the model were initialized to zero, corresponding to a not excited network ($S_1 = S_0 = 0$).

$$
L(F^0) = \begin{bmatrix} X_1(t=0)\\ X_2(t=0)\\ X_3(t=0)\\ Y_1(t=0)\\ Y_2(t=0)\\ Y_3(t=0) \end{bmatrix} = \begin{bmatrix} 0\\0\\0\\0\\0\\0 \end{bmatrix}
$$

Inputs:

The inputs $S_1(t)$ and $S_2(t)$ were set to piecewise-constant trajectories, corresponding to different "on/off" combinations. The switching times between the input combinations were chosen such that all species could reach their corresponding steady-state concentrations in each interval.

$$
[S_1(t), S_2(t)] = \begin{cases} [0,0] & 0 \le t < 20,\\ [5,0] & 20 \le t < 40,\\ [5,5] & 40 \le t < 80,\\ [0,5] & 80 \le t < 100,\\ [5,5] & 100 \le t. \end{cases}
$$

Complete Model

Ordinary differential equations:

$$
\frac{d}{dt}\begin{bmatrix} X_1\\ X_2\\ X_3\\ Y_1\\ Y_2\\ Y_3 \end{bmatrix} = \begin{bmatrix} -1 & 1 & 0 & 0 & 0 & 0 & -1 & 0 & 0 & 0\\ 1 & 0 & -1 & 0 & 0 & 0 & 0 & 1 & 0 & 0\\ 0 & 0 & 1 & 0 & 0 & 0 & 0 & 0 & -1 & 0\\ 0 & 0 & 0 & 1 & -1 & 0 & 0 & -1 & 0 & 0\\ 0 & 0 & 0 & 0 & 1 & -1 & 1 & 0 & 0 & 0\\ 0 & 0 & 0 & 0 & 0 & 1 & 0 & 0 & 0 & -1 \end{bmatrix} \begin{bmatrix} v_1\\ v_2\\ v_3\\ v_4\\ v_5\\ v_6\\ v_7\\ v_8\\ v_9\\ v_{10} \end{bmatrix},
$$

with

$$
v_1 = -\,\mathrm{k_{12x}}\,\mathrm{C}\,\mathrm{X_1}\,(\mathrm{X_2} - \mathrm{X_{2T}})
$$

$$
\begin{aligned}
v_2 &= -\frac{\mathrm{C\,k_x\,S_1(t)\,K_{mx}\,(X_1 - X_{1T})}}{\mathrm{S_1(t) + K_{mx}}} \\
v_3 &= -\,\mathrm{k_{23x}\,C\,X_2\,(X_3 - X_{3T})} \\
v_4 &= -\frac{\mathrm{S_2(t)\,k_y\,K_{my}\,C\,(Y_1 - Y_{1T})}}{\mathrm{S_2(t) + K_{my}}} \\
v_5 &= -\,\mathrm{k_{12y}\,C\,Y_1\,(Y_2 - Y_{2T})} \\
v_6 &= -\,\mathrm{k_{23y}\,C\,Y_2\,(Y_3 - Y_{3T})} \\
v_7 &= -\,\mathrm{k_a\,C\,X_1\,(Y_2 - Y_{2T})} \\
v_8 &= -\,\mathrm{k_a\,C\,Y_1\,(X_2 - X_{2T})} \\
v_9 &= \frac{\mathrm{k_d\,K_{myx}\,C\,X_3\,Y_3}}{\mathrm{K_{myx} + X_3}} \\
v_{10} &= \frac{\mathrm{k_d\,K_{mxy}\,C\,X_3\,Y_3}}{\mathrm{K_{mxy} + Y_3}}
\end{aligned}
$$

Choosing the Layers to Simulate

As noted in the main text, not all layers (see below) have to be simulated in all cases. If only the layer dynamics for a given order are of interest, it is sufficient to simulate the respective $N_L = 4$ conditional dynamics. For example, for the order $F^1 \to F^2 \to F^3 \to F^4$, it is sufficient to simulate the layer dynamics $L(F^1|F^0)$, $L(F^2|F^0, F^1)$, $L(F^3|F^0, F^1, F^2)$, and $L(F^4|F^0, F^1, F^2, F^3)$. Similarly, for the order $F^3 \to F^2 \to F^4 \to F^1$ it is sufficient to simulate the layer dynamics $L(F^3|F^0)$, $L(F^2|F^0, F^3)$, $L(F^4|F^0, F^2, F^3)$, and $L(F^1|F^0, F^2, F^3, F^4)$. The complete model dynamics can then be calculated by

$$
\begin{aligned}
L(F^0, F^1, F^2, F^3, F^4) &= L(F^0) + L(F^1|F^0) + L(F^2|F^0, F^1) + L(F^3|F^0, F^1, F^2) + L(F^4|F^0, F^1, F^2, F^3) \\
&= L(F^0) + L(F^3|F^0) + L(F^2|F^0, F^3) + L(F^4|F^0, F^2, F^3) + L(F^1|F^0, F^2, F^3, F^4).
\end{aligned}
$$

If the layer dynamics for all orderings of the functionalities are of interest, the lemma analogous to Bayes' rule derived in the main text allows to significantly reduce the number of necessary numerical integrations. It is for example sufficient to simulate the $2^{N_L} - 1 = 15$ layer dynamics

$$
\begin{array}{lllll}
L(F^1|F^0), & L(F^2|F^0), & L(F^3|F^0), & L(F^4|F^0), & L(F^2|F^0, F^1), \\
L(F^3|F^0, F^1), & L(F^4|F^0, F^1), & L(F^3|F^0, F^2), & L(F^4|F^0, F^2), & L(F^4|F^0, F^3), \\
L(F^3|F^0, F^1, F^2), & L(F^4|F^0, F^1, F^2), & L(F^4|F^0, F^1, F^3), & L(F^4|F^0, F^2, F^3), & L(F^4|F^0, F^1, F^2, F^3).
\end{array}
$$

Then, by using Bayes' rule, the layer dynamics $L(F^1|F^0, F^2)$ can be calculated by

$$
L(F^1|F^0, F^2) = L(F^2|F^0, F^1) + L(F^1|F^0) - L(F^2|F^0).
$$

As a general rule, for each non-empty subset of the functionalities $\mathbb{F} \subseteq \{F^1, \ldots, F^{N_L}\}$, $\mathbb{F} \neq \{\}$, one layer has to be simulated. The decision on which functionality $F^i \in \mathbb{F}$ the layer $L(F^i|F^0, \mathbb{F} \setminus F^i)$ represents, conditioned on the rest $\mathbb{F} \setminus F^i$ of the functionalities and the initial condition represented by $L(F^0)$, is thereby arbitrary. For example, for the functionalities $\{F^1, F^2, F^4\}$ we can choose to simulate one of $L(F^1|F^0, F^2, F^4)$, $L(F^2|F^0, F^1, F^4)$, or $L(F^4|F^0, F^1, F^2)$. The other layers' dynamics can then be derived by applying Bayes' rule.

For Example 1 in the main text, it is possible to further reduce the number of necessary simulations. It is easy to validate that the incompatibility between the two isolated pathways (i.e. without any crosstalk mechanism) is zero: $I(F^1, F^2|F^0) = 0$. Thus, the dynamics of $L(F^2|F^0, F^1)$ equal the dynamics of

$L(F^2|F^0)$, reducing the number of layers necessary to simulate. Similarly, $L(F^4|F^0, F^3) = L(F^4|F^0)$. Finally, both crosstalk mechanisms in isolation cannot sense a change in the input signals S_1 and S_2. Given the initial conditions $L(F^0)$, this implies that $L(F^3|F^0) = L(F^4|F^0) = 0$. In summary, only 11 layers have to be simulated for the first model.

Layer $L(F^1|F^0)$

Required input trajectories:

$$\begin{bmatrix} X_1^0 \\ X_2^0 \\ X_3^0 \end{bmatrix} = \begin{bmatrix} 1 & 0 & 0 & 0 & 0 & 0 \\ 0 & 1 & 0 & 0 & 0 & 0 \\ 0 & 0 & 1 & 0 & 0 & 0 \end{bmatrix} L(F^0).$$

Ordinary differential equations:

$$\frac{d}{dt} \begin{bmatrix} X_1^1 \\ X_2^1 \\ X_3^1 \end{bmatrix} = \begin{bmatrix} -1 & 1 & 0 \\ 1 & 0 & -1 \\ 0 & 0 & 1 \end{bmatrix} \begin{bmatrix} v_1 \\ v_2 \\ v_3 \end{bmatrix},$$

with

$$\begin{aligned} v_1 &= -\,\mathrm{k_{12x}}\,\mathrm{C}\,\left(\mathrm{X_1^0} + \mathrm{X_1^1}\right)\left(\mathrm{X_2^0} - \mathrm{X_{2T}} + \mathrm{X_2^1}\right) \\ v_2 &= -\,\frac{\mathrm{C}\,\mathrm{k_x}\,\mathrm{S_1(t)}\,\mathrm{K_{mx}}\,\left(\mathrm{X_1^0} - \mathrm{X_{1T}} + \mathrm{X_1^1}\right)}{\mathrm{S_1(t)} + \mathrm{K_{mx}}} \\ v_3 &= -\,\mathrm{k_{23x}}\,\mathrm{C}\,\left(\mathrm{X_2^0} + \mathrm{X_2^1}\right)\left(\mathrm{X_3^0} - \mathrm{X_{3T}} + \mathrm{X_3^1}\right) \end{aligned}$$

Layer Dynamics:

$$L(F^1|F^0) = \begin{bmatrix} X_1^1 & X_2^1 & X_3^1 & 0 & 0 & 0 \end{bmatrix}^T.$$

Layer $L(F^1|F^0, F^2)$

Required input trajectories:

$$\begin{bmatrix} X_1^{0,2} \\ X_2^{0,2} \\ X_3^{0,2} \end{bmatrix} = \begin{bmatrix} 1 & 0 & 0 & 0 & 0 & 0 \\ 0 & 1 & 0 & 0 & 0 & 0 \\ 0 & 0 & 1 & 0 & 0 & 0 \end{bmatrix} L(F^0, F^2).$$

Ordinary differential equations:

$$\frac{d}{dt} \begin{bmatrix} X_1^1 \\ X_2^1 \\ X_3^1 \end{bmatrix} = \begin{bmatrix} -1 & 1 & 0 \\ 1 & 0 & -1 \\ 0 & 0 & 1 \end{bmatrix} \begin{bmatrix} v_1 \\ v_2 \\ v_3 \end{bmatrix},$$

with

$$\begin{aligned} v_1 &= -\,\mathrm{k_{12x}}\,\mathrm{C}\,\left(\mathrm{X_1^{0,2}} + \mathrm{X_1^1}\right)\left(\mathrm{X_2^{0,2}} - \mathrm{X_{2T}} + \mathrm{X_2^1}\right) \\ v_2 &= -\,\frac{\mathrm{C}\,\mathrm{k_x}\,\mathrm{S_1(t)}\,\mathrm{K_{mx}}\,\left(\mathrm{X_1^{0,2}} - \mathrm{X_{1T}} + \mathrm{X_1^1}\right)}{\mathrm{S_1(t)} + \mathrm{K_{mx}}} \\ v_3 &= -\,\mathrm{k_{23x}}\,\mathrm{C}\,\left(\mathrm{X_2^{0,2}} + \mathrm{X_2^1}\right)\left(\mathrm{X_3^{0,2}} - \mathrm{X_{3T}} + \mathrm{X_3^1}\right) \end{aligned}$$

Layer Dynamics:

$$L(F^1|F^0,F^2) = \begin{bmatrix} X_1^1 & X_2^1 & X_3^1 & 0 & 0 & 0 \end{bmatrix}^T.$$

Layer $L(F^1|F^0,F^3)$

Required input trajectories:

$$\begin{bmatrix} X_1^{0,3} \\ X_2^{0,3} \\ X_3^{0,3} \\ Y_3^{0,3} \end{bmatrix} = \begin{bmatrix} 1 & 0 & 0 & 0 & 0 & 0 \\ 0 & 1 & 0 & 0 & 0 & 0 \\ 0 & 0 & 1 & 0 & 0 & 0 \\ 0 & 0 & 0 & 0 & 0 & 1 \end{bmatrix} L(F^0,F^3).$$

Ordinary differential equations:

$$\frac{d}{dt}\begin{bmatrix} X_1^1 \\ X_2^1 \\ X_3^1 \\ Y_3^1 \end{bmatrix} = \begin{bmatrix} -1 & 1 & 0 \\ 1 & 0 & -1 \\ 0 & 0 & 1 \\ 0 & 0 & 0 \end{bmatrix} \begin{bmatrix} v_1 \\ v_2 \\ v_3 \end{bmatrix} + \begin{bmatrix} 0 & 0 \\ 0 & 0 \\ -1 & 0 \\ 0 & -1 \end{bmatrix} \begin{bmatrix} v_{alt,9} \\ v_{alt,10} \end{bmatrix},$$

with

$$\begin{aligned}
v_1 =& -\mathrm{k_{12x}}\,\mathrm{C}\left(\mathrm{X_1^{0,3}} + \mathrm{X_1^1}\right)\left(\mathrm{X_2^{0,3}} - \mathrm{X_{2T}} + \mathrm{X_2^1}\right) \\
v_2 =& -\frac{\mathrm{C}\,\mathrm{k_x}\,\mathrm{S_1(t)}\,\mathrm{K_{mx}}\left(\mathrm{X_1^{0,3}} - \mathrm{X_{1T}} + \mathrm{X_1^1}\right)}{\mathrm{S_1(t)} + \mathrm{K_{mx}}} \\
v_3 =& -\mathrm{k_{23x}}\,\mathrm{C}\left(\mathrm{X_2^{0,3}} + \mathrm{X_2^1}\right)\left(\mathrm{X_3^{0,3}} - \mathrm{X_{3T}} + \mathrm{X_3^1}\right) \\
v_{alt,9} =& \frac{\mathrm{k_d}\,\mathrm{C}\left(\mathrm{X_3^{0,3}} + \mathrm{X_3^1}\right)\left(\mathrm{Y_3^{0,3}} + \mathrm{Y_3^1}\right)}{\frac{\mathrm{X_3^{0,3}}+\mathrm{X_3^1}}{\mathrm{K_{myx}}} + 1} - \frac{\mathrm{k_d}\,\mathrm{C}\,\mathrm{X_3^{0,3}}\,\mathrm{Y_3^{0,3}}}{\frac{\mathrm{X_3^{0,3}}}{\mathrm{K_{myx}}} + 1} \\
v_{alt,10} =& \frac{\mathrm{k_d}\,\mathrm{C}\left(\mathrm{X_3^{0,3}} + \mathrm{X_3^1}\right)\left(\mathrm{Y_3^{0,3}} + \mathrm{Y_3^1}\right)}{\frac{\mathrm{Y_3^{0,3}}+\mathrm{Y_3^1}}{\mathrm{K_{mxy}}} + 1} - \frac{\mathrm{k_d}\,\mathrm{C}\,\mathrm{X_3^{0,3}}\,\mathrm{Y_3^{0,3}}}{\frac{\mathrm{Y_3^{0,3}}}{\mathrm{K_{mxy}}} + 1}
\end{aligned}$$

Layer Dynamics:

$$L(F^1|F^0,F^3) = \begin{bmatrix} X_1^1 & X_2^1 & X_3^1 & 0 & 0 & Y_3^1 \end{bmatrix}^T.$$

Layer $L(F^1|F^0,F^4)$

Required input trajectories:

$$\begin{bmatrix} X_1^{0,4} \\ X_2^{0,4} \\ X_3^{0,4} \\ Y_1^{0,4} \\ Y_2^{0,4} \end{bmatrix} = \begin{bmatrix} 1 & 0 & 0 & 0 & 0 & 0 \\ 0 & 1 & 0 & 0 & 0 & 0 \\ 0 & 0 & 1 & 0 & 0 & 0 \\ 0 & 0 & 0 & 1 & 0 & 0 \\ 0 & 0 & 0 & 0 & 1 & 0 \end{bmatrix} L(F^0,F^4).$$

Ordinary differential equations:

$$\frac{d}{dt}\begin{bmatrix}X_1^1\\X_2^1\\X_3^1\\Y_1^1\\Y_2^1\end{bmatrix}=\begin{bmatrix}-1&1&0\\1&0&-1\\0&0&1\\0&0&0\\0&0&0\end{bmatrix}\begin{bmatrix}v_1\\v_2\\v_3\end{bmatrix}+\begin{bmatrix}-1&0\\0&1\\0&0\\0&-1\\1&0\end{bmatrix}\begin{bmatrix}v_{alt,7}\\v_{alt,8}\end{bmatrix},$$

with

$$\begin{aligned}
v_1 &= -\,\mathrm{k_{12x}}\,\mathrm{C}\left(\mathrm{X}_1^{0,4}+\mathrm{X}_1^1\right)\left(\mathrm{X}_2^{0,4}-\mathrm{X_{2T}}+\mathrm{X}_2^1\right)\\
v_2 &= -\,\frac{\mathrm{C}\,\mathrm{k_x}\,\mathrm{S_1(t)}\,\mathrm{K_{mx}}\left(\mathrm{X}_1^{0,4}-\mathrm{X_{1T}}+\mathrm{X}_1^1\right)}{\mathrm{S_1(t)}+\mathrm{K_{mx}}}\\
v_3 &= -\,\mathrm{k_{23x}}\,\mathrm{C}\left(\mathrm{X}_2^{0,4}+\mathrm{X}_2^1\right)\left(\mathrm{X}_3^{0,4}-\mathrm{X_{3T}}+\mathrm{X}_3^1\right)\\
v_{alt,7} &= -\,\mathrm{k_a}\,\mathrm{C}\,\mathrm{X}_1^{0,4}\,\mathrm{Y}_2^1-\mathrm{k_a}\,\mathrm{C}\,\mathrm{X}_1^1\left(\mathrm{Y}_2^{0,4}-\mathrm{Y_{2T}}+\mathrm{Y}_2^1\right)\\
v_{alt,8} &= -\,\mathrm{k_a}\,\mathrm{C}\,\mathrm{X}_2^1\,\mathrm{Y}_1^{0,4}-\mathrm{k_a}\,\mathrm{C}\,\mathrm{Y}_1^1\left(\mathrm{X}_2^{0,4}-\mathrm{X_{2T}}+\mathrm{X}_2^1\right)
\end{aligned}$$

Layer Dynamics:

$$L(F^1|F^0,F^4)=\begin{bmatrix}X_1^1 & X_2^1 & X_3^1 & Y_1^1 & Y_2^1 & 0\end{bmatrix}^T.$$

Layer $L(F^1|F^0,F^2,F^3)$

Required input trajectories:

$$\begin{bmatrix}X_1^{0,2,3}\\X_2^{0,2,3}\\X_3^{0,2,3}\\Y_1^{0,2,3}\\Y_2^{0,2,3}\\Y_3^{0,2,3}\end{bmatrix}=\begin{bmatrix}1&0&0&0&0&0\\0&1&0&0&0&0\\0&0&1&0&0&0\\0&0&0&1&0&0\\0&0&0&0&1&0\\0&0&0&0&0&1\end{bmatrix}L(F^0,F^2,F^3).$$

Ordinary differential equations:

$$\frac{d}{dt}\begin{bmatrix}X_1^1\\X_2^1\\X_3^1\\Y_1^1\\Y_2^1\\Y_3^1\end{bmatrix}=\begin{bmatrix}-1&1&0\\1&0&-1\\0&0&1\\0&0&0\\0&0&0\\0&0&0\end{bmatrix}\begin{bmatrix}v_1\\v_2\\v_3\end{bmatrix}+\begin{bmatrix}0&0&0&0&0\\0&0&0&0&0\\0&0&0&-1&0\\1&-1&0&0&0\\0&1&-1&0&0\\0&0&1&0&-1\end{bmatrix}\begin{bmatrix}v_{alt,4}\\v_{alt,5}\\v_{alt,6}\\v_{alt,9}\\v_{alt,10}\end{bmatrix},$$

with

$$\begin{aligned}
v_1 &= -\,\mathrm{k_{12x}}\,\mathrm{C}\left(\mathrm{X}_1^{0,2,3}+\mathrm{X}_1^1\right)\left(\mathrm{X}_2^{0,2,3}-\mathrm{X_{2T}}+\mathrm{X}_2^1\right)\\
v_2 &= -\,\frac{\mathrm{C}\,\mathrm{k_x}\,\mathrm{S_1(t)}\,\mathrm{K_{mx}}\left(\mathrm{X}_1^{0,2,3}-\mathrm{X_{1T}}+\mathrm{X}_1^1\right)}{\mathrm{S_1(t)}+\mathrm{K_{mx}}}
\end{aligned}$$

$$
\begin{aligned}
v_3 =& - \mathrm{k_{23x}}\,\mathrm{C}\,\left(\mathrm{X_2^{0,2,3}} + \mathrm{X_2^1}\right)\,\left(\mathrm{X_3^{0,2,3}} - \mathrm{X_{3T}} + \mathrm{X_3^1}\right)\\
v_{alt,4} =& - \frac{\mathrm{S_2(t)\,k_y\,K_{my}\,C\,Y_1^1}}{\mathrm{S_2(t) + K_{my}}}\\
v_{alt,5} =& - \mathrm{k_{12y}\,C\,Y_1^{0,2,3}\,Y_2^1} - \mathrm{k_{12y}\,C\,Y_1^1}\,\left(\mathrm{Y_2^{0,2,3}} - \mathrm{Y_{2T}} + \mathrm{Y_2^1}\right)\\
v_{alt,6} =& - \mathrm{k_{23y}\,C\,Y_2^{0,2,3}\,Y_3^1} - \mathrm{k_{23y}\,C\,Y_2^1}\,\left(\mathrm{Y_3^{0,2,3}} - \mathrm{Y_{3T}} + \mathrm{Y_3^1}\right)\\
v_{alt,9} =& \frac{\mathrm{k_d\,C}\,\left(\mathrm{X_3^{0,2,3}} + \mathrm{X_3^1}\right)\,\left(\mathrm{Y_3^{0,2,3}} + \mathrm{Y_3^1}\right)}{\frac{\mathrm{X_3^{0,2,3}+X_3^1}}{\mathrm{K_{myx}}} + 1} - \frac{\mathrm{k_d\,C\,X_3^{0,2,3}\,Y_3^{0,2,3}}}{\frac{\mathrm{X_3^{0,2,3}}}{\mathrm{K_{myx}}} + 1}\\
v_{alt,10} =& \frac{\mathrm{k_d\,C}\,\left(\mathrm{X_3^{0,2,3}} + \mathrm{X_3^1}\right)\,\left(\mathrm{Y_3^{0,2,3}} + \mathrm{Y_3^1}\right)}{\frac{\mathrm{Y_3^{0,2,3}+Y_3^1}}{\mathrm{K_{mxy}}} + 1} - \frac{\mathrm{k_d\,C\,X_3^{0,2,3}\,Y_3^{0,2,3}}}{\frac{\mathrm{Y_3^{0,2,3}}}{\mathrm{K_{mxy}}} + 1}
\end{aligned}
$$

Layer Dynamics:

$$
L(F^1|F^0, F^2, F^3) = \begin{bmatrix} X_1^1 & X_2^1 & X_3^1 & Y_1^1 & Y_2^1 & Y_3^1 \end{bmatrix}^T.
$$

Layer $L(F^1|F^0, F^2, F^4)$

Required input trajectories:

$$
\begin{bmatrix} X_1^{0,2,4} \\ X_2^{0,2,4} \\ X_3^{0,2,4} \\ Y_1^{0,2,4} \\ Y_2^{0,2,4} \\ Y_3^{0,2,4} \end{bmatrix} = \begin{bmatrix} 1 & 0 & 0 & 0 & 0 & 0 \\ 0 & 1 & 0 & 0 & 0 & 0 \\ 0 & 0 & 1 & 0 & 0 & 0 \\ 0 & 0 & 0 & 1 & 0 & 0 \\ 0 & 0 & 0 & 0 & 1 & 0 \\ 0 & 0 & 0 & 0 & 0 & 1 \end{bmatrix} L(F^0, F^2, F^4).
$$

Ordinary differential equations:

$$
\frac{d}{dt}\begin{bmatrix} X_1^1 \\ X_2^1 \\ X_3^1 \\ Y_1^1 \\ Y_2^1 \\ Y_3^1 \end{bmatrix} = \begin{bmatrix} -1 & 1 & 0 \\ 1 & 0 & -1 \\ 0 & 0 & 1 \\ 0 & 0 & 0 \\ 0 & 0 & 0 \\ 0 & 0 & 0 \end{bmatrix} \begin{bmatrix} v_1 \\ v_2 \\ v_3 \end{bmatrix} + \begin{bmatrix} 0 & 0 & 0 & -1 & 0 \\ 0 & 0 & 0 & 0 & 1 \\ 0 & 0 & 0 & 0 & 0 \\ 1 & -1 & 0 & 0 & -1 \\ 0 & 1 & -1 & 1 & 0 \\ 0 & 0 & 1 & 0 & 0 \end{bmatrix} \begin{bmatrix} v_{alt,4} \\ v_{alt,5} \\ v_{alt,6} \\ v_{alt,7} \\ v_{alt,8} \end{bmatrix},
$$

with

$$
\begin{aligned}
v_1 =& - \mathrm{k_{12x}}\,\mathrm{C}\,\left(\mathrm{X_1^{0,2,4}} + \mathrm{X_1^1}\right)\,\left(\mathrm{X_2^{0,2,4}} - \mathrm{X_{2T}} + \mathrm{X_2^1}\right)\\
v_2 =& - \frac{\mathrm{C\,k_x\,S_1(t)\,K_{mx}}\,\left(\mathrm{X_1^{0,2,4}} - \mathrm{X_{1T}} + \mathrm{X_1^1}\right)}{\mathrm{S_1(t) + K_{mx}}}\\
v_3 =& - \mathrm{k_{23x}}\,\mathrm{C}\,\left(\mathrm{X_2^{0,2,4}} + \mathrm{X_2^1}\right)\,\left(\mathrm{X_3^{0,2,4}} - \mathrm{X_{3T}} + \mathrm{X_3^1}\right)\\
v_{alt,4} =& - \frac{\mathrm{S_2(t)\,k_y\,K_{my}\,C\,Y_1^1}}{\mathrm{S_2(t) + K_{my}}}
\end{aligned}
$$

$$
\begin{aligned}
v_{alt,5} =& - \mathrm{k_{12y}}\,\mathrm{C}\,\mathrm{Y_1^{0,2,4}}\,\mathrm{Y_2^1} - \mathrm{k_{12y}}\,\mathrm{C}\,\mathrm{Y_1^1}\,\left(\mathrm{Y_2^{0,2,4}} - \mathrm{Y_{2T}} + \mathrm{Y_2^1}\right)\\
v_{alt,6} =& - \mathrm{k_{23y}}\,\mathrm{C}\,\mathrm{Y_2^{0,2,4}}\,\mathrm{Y_3^1} - \mathrm{k_{23y}}\,\mathrm{C}\,\mathrm{Y_2^1}\,\left(\mathrm{Y_3^{0,2,4}} - \mathrm{Y_{3T}} + \mathrm{Y_3^1}\right)\\
v_{alt,7} =& - \mathrm{k_a}\,\mathrm{C}\,\mathrm{X_1^{0,2,4}}\,\mathrm{Y_2^1} - \mathrm{k_a}\,\mathrm{C}\,\mathrm{X_1^1}\,\left(\mathrm{Y_2^{0,2,4}} - \mathrm{Y_{2T}} + \mathrm{Y_2^1}\right)\\
v_{alt,8} =& - \mathrm{k_a}\,\mathrm{C}\,\mathrm{X_2^1}\,\mathrm{Y_1^{0,2,4}} - \mathrm{k_a}\,\mathrm{C}\,\mathrm{Y_1^1}\,\left(\mathrm{X_2^{0,2,4}} - \mathrm{X_{2T}} + \mathrm{X_2^1}\right)
\end{aligned}
$$

Layer Dynamics:

$$L(F^1|F^0,F^2,F^4) = \begin{bmatrix} X_1^1 & X_2^1 & X_3^1 & Y_1^1 & Y_2^1 & Y_3^1 \end{bmatrix}^T.$$

Layer $L(F^1|F^0,F^3,F^4)$

Required input trajectories:

$$\begin{bmatrix} X_1^{0,3,4} \\ X_2^{0,3,4} \\ X_3^{0,3,4} \\ Y_1^{0,3,4} \\ Y_2^{0,3,4} \\ Y_3^{0,3,4} \end{bmatrix} = \begin{bmatrix} 1 & 0 & 0 & 0 & 0 & 0 \\ 0 & 1 & 0 & 0 & 0 & 0 \\ 0 & 0 & 1 & 0 & 0 & 0 \\ 0 & 0 & 0 & 1 & 0 & 0 \\ 0 & 0 & 0 & 0 & 1 & 0 \\ 0 & 0 & 0 & 0 & 0 & 1 \end{bmatrix} L(F^0,F^3,F^4).$$

Ordinary differential equations:

$$\frac{d}{dt}\begin{bmatrix} X_1^1 \\ X_2^1 \\ X_3^1 \\ Y_1^1 \\ Y_2^1 \\ Y_3^1 \end{bmatrix} = \begin{bmatrix} -1 & 1 & 0 \\ 1 & 0 & -1 \\ 0 & 0 & 1 \\ 0 & 0 & 0 \\ 0 & 0 & 0 \\ 0 & 0 & 0 \end{bmatrix} \begin{bmatrix} v_1 \\ v_2 \\ v_3 \end{bmatrix} + \begin{bmatrix} 0 & 0 & -1 & 0 \\ 0 & 0 & 0 & 1 \\ -1 & 0 & 0 & 0 \\ 0 & 0 & 0 & -1 \\ 0 & 0 & 1 & 0 \\ 0 & -1 & 0 & 0 \end{bmatrix} \begin{bmatrix} v_{alt,9} \\ v_{alt,10} \\ v_{alt,7} \\ v_{alt,8} \end{bmatrix},$$

with

$$
\begin{aligned}
v_1 =& - \mathrm{k_{12x}}\,\mathrm{C}\,\left(\mathrm{X_1^{0,3,4}} + \mathrm{X_1^1}\right)\,\left(\mathrm{X_2^{0,3,4}} - \mathrm{X_{2T}} + \mathrm{X_2^1}\right)\\
v_2 =& - \frac{\mathrm{C}\,\mathrm{k_x}\,\mathrm{S_1(t)}\,\mathrm{K_{mx}}\,\left(\mathrm{X_1^{0,3,4}} - \mathrm{X_{1T}} + \mathrm{X_1^1}\right)}{\mathrm{S_1(t)} + \mathrm{K_{mx}}}\\
v_3 =& - \mathrm{k_{23x}}\,\mathrm{C}\,\left(\mathrm{X_2^{0,3,4}} + \mathrm{X_2^1}\right)\,\left(\mathrm{X_3^{0,3,4}} - \mathrm{X_{3T}} + \mathrm{X_3^1}\right)\\
v_{alt,9} =& \frac{\mathrm{k_d}\,\mathrm{C}\,\left(\mathrm{X_3^{0,3,4}} + \mathrm{X_3^1}\right)\,\left(\mathrm{Y_3^{0,3,4}} + \mathrm{Y_3^1}\right)}{\frac{\mathrm{X_3^{0,3,4}} + \mathrm{X_3^1}}{\mathrm{K_{myx}}} + 1} - \frac{\mathrm{k_d}\,\mathrm{C}\,\mathrm{X_3^{0,3,4}}\,\mathrm{Y_3^{0,3,4}}}{\frac{\mathrm{X_3^{0,3,4}}}{\mathrm{K_{myx}}} + 1}\\
v_{alt,10} =& \frac{\mathrm{k_d}\,\mathrm{C}\,\left(\mathrm{X_3^{0,3,4}} + \mathrm{X_3^1}\right)\,\left(\mathrm{Y_3^{0,3,4}} + \mathrm{Y_3^1}\right)}{\frac{\mathrm{Y_3^{0,3,4}} + \mathrm{Y_3^1}}{\mathrm{K_{mxy}}} + 1} - \frac{\mathrm{k_d}\,\mathrm{C}\,\mathrm{X_3^{0,3,4}}\,\mathrm{Y_3^{0,3,4}}}{\frac{\mathrm{Y_3^{0,3,4}}}{\mathrm{K_{mxy}}} + 1}\\
v_{alt,7} =& - \mathrm{k_a}\,\mathrm{C}\,\mathrm{X_1^{0,3,4}}\,\mathrm{Y_2^1} - \mathrm{k_a}\,\mathrm{C}\,\mathrm{X_1^1}\,\left(\mathrm{Y_2^{0,3,4}} - \mathrm{Y_{2T}} + \mathrm{Y_2^1}\right)\\
v_{alt,8} =& - \mathrm{k_a}\,\mathrm{C}\,\mathrm{X_2^1}\,\mathrm{Y_1^{0,3,4}} - \mathrm{k_a}\,\mathrm{C}\,\mathrm{Y_1^1}\,\left(\mathrm{X_2^{0,3,4}} - \mathrm{X_{2T}} + \mathrm{X_2^1}\right)
\end{aligned}
$$

Layer Dynamics:

$$L(F^1|F^0,F^3,F^4) = \begin{bmatrix} X_1^1 & X_2^1 & X_3^1 & Y_1^1 & Y_2^1 & Y_3^1 \end{bmatrix}^T.$$

Layer $L(F^1|F^0,F^2,F^3,F^4)$

Required input trajectories:

$$\begin{bmatrix} X_1^{0,2,3,4} \\ X_2^{0,2,3,4} \\ X_3^{0,2,3,4} \\ Y_1^{0,2,3,4} \\ Y_2^{0,2,3,4} \\ Y_3^{0,2,3,4} \end{bmatrix} = \begin{bmatrix} 1 & 0 & 0 & 0 & 0 & 0 \\ 0 & 1 & 0 & 0 & 0 & 0 \\ 0 & 0 & 1 & 0 & 0 & 0 \\ 0 & 0 & 0 & 1 & 0 & 0 \\ 0 & 0 & 0 & 0 & 1 & 0 \\ 0 & 0 & 0 & 0 & 0 & 1 \end{bmatrix} L(F^0,F^2,F^3,F^4).$$

Ordinary differential equations:

$$\frac{d}{dt}\begin{bmatrix} X_1^1 \\ X_2^1 \\ X_3^1 \\ Y_1^1 \\ Y_2^1 \\ Y_3^1 \end{bmatrix} = \begin{bmatrix} -1 & 1 & 0 \\ 1 & 0 & -1 \\ 0 & 0 & 1 \\ 0 & 0 & 0 \\ 0 & 0 & 0 \\ 0 & 0 & 0 \end{bmatrix} \begin{bmatrix} v_1 \\ v_2 \\ v_3 \end{bmatrix} + \begin{bmatrix} 0 & 0 & 0 & -1 & 0 & 0 & 0 \\ 0 & 0 & 0 & 0 & 1 & 0 & 0 \\ 0 & 0 & 0 & 0 & 0 & -1 & 0 \\ 1 & -1 & 0 & 0 & -1 & 0 & 0 \\ 0 & 1 & -1 & 1 & 0 & 0 & 0 \\ 0 & 0 & 1 & 0 & 0 & 0 & -1 \end{bmatrix} \begin{bmatrix} v_{alt,4} \\ v_{alt,5} \\ v_{alt,6} \\ v_{alt,7} \\ v_{alt,8} \\ v_{alt,9} \\ v_{alt,10} \end{bmatrix},$$

with

$$\begin{aligned}
v_1 &= -\,\mathrm{k_{12x}}\,\mathrm{C}\left(\mathrm{X}_1^{0,2,3,4} + \mathrm{X}_1^1\right)\left(\mathrm{X}_2^{0,2,3,4} - \mathrm{X_{2T}} + \mathrm{X}_2^1\right) \\
v_2 &= -\,\frac{\mathrm{C}\,\mathrm{k_x}\,\mathrm{S_1(t)}\,\mathrm{K_{mx}}\left(\mathrm{X}_1^{0,2,3,4} - \mathrm{X_{1T}} + \mathrm{X}_1^1\right)}{\mathrm{S_1(t)} + \mathrm{K_{mx}}} \\
v_3 &= -\,\mathrm{k_{23x}}\,\mathrm{C}\left(\mathrm{X}_2^{0,2,3,4} + \mathrm{X}_2^1\right)\left(\mathrm{X}_3^{0,2,3,4} - \mathrm{X_{3T}} + \mathrm{X}_3^1\right) \\
v_{alt,4} &= -\,\frac{\mathrm{S_2(t)}\,\mathrm{k_y}\,\mathrm{K_{my}}\,\mathrm{C}\,\mathrm{Y}_1^1}{\mathrm{S_2(t)} + \mathrm{K_{my}}} \\
v_{alt,5} &= -\,\mathrm{k_{12y}}\,\mathrm{C}\,\mathrm{Y}_1^{0,2,3,4}\,\mathrm{Y}_2^1 - \mathrm{k_{12y}}\,\mathrm{C}\,\mathrm{Y}_1^1\left(\mathrm{Y}_2^{0,2,3,4} - \mathrm{Y_{2T}} + \mathrm{Y}_2^1\right) \\
v_{alt,6} &= -\,\mathrm{k_{23y}}\,\mathrm{C}\,\mathrm{Y}_2^{0,2,3,4}\,\mathrm{Y}_3^1 - \mathrm{k_{23y}}\,\mathrm{C}\,\mathrm{Y}_2^1\left(\mathrm{Y}_3^{0,2,3,4} - \mathrm{Y_{3T}} + \mathrm{Y}_3^1\right) \\
v_{alt,7} &= -\,\mathrm{k_a}\,\mathrm{C}\,\mathrm{X}_1^{0,2,3,4}\,\mathrm{Y}_2^1 - \mathrm{k_a}\,\mathrm{C}\,\mathrm{X}_1^1\left(\mathrm{Y}_2^{0,2,3,4} - \mathrm{Y_{2T}} + \mathrm{Y}_2^1\right) \\
v_{alt,8} &= -\,\mathrm{k_a}\,\mathrm{C}\,\mathrm{X}_2^1\,\mathrm{Y}_1^{0,2,3,4} - \mathrm{k_a}\,\mathrm{C}\,\mathrm{Y}_1^1\left(\mathrm{X}_2^{0,2,3,4} - \mathrm{X_{2T}} + \mathrm{X}_2^1\right) \\
v_{alt,9} &= \frac{\mathrm{k_d}\,\mathrm{C}\left(\mathrm{X}_3^{0,2,3,4} + \mathrm{X}_3^1\right)\left(\mathrm{Y}_3^{0,2,3,4} + \mathrm{Y}_3^1\right)}{\frac{\mathrm{X}_3^{0,2,3,4} + \mathrm{X}_3^1}{\mathrm{K_{myx}}} + 1} - \frac{\mathrm{k_d}\,\mathrm{C}\,\mathrm{X}_3^{0,2,3,4}\,\mathrm{Y}_3^{0,2,3,4}}{\frac{\mathrm{X}_3^{0,2,3,4}}{\mathrm{K_{myx}}} + 1} \\
v_{alt,10} &= \frac{\mathrm{k_d}\,\mathrm{C}\left(\mathrm{X}_3^{0,2,3,4} + \mathrm{X}_3^1\right)\left(\mathrm{Y}_3^{0,2,3,4} + \mathrm{Y}_3^1\right)}{\frac{\mathrm{Y}_3^{0,2,3,4} + \mathrm{Y}_3^1}{\mathrm{K_{mxy}}} + 1} - \frac{\mathrm{k_d}\,\mathrm{C}\,\mathrm{X}_3^{0,2,3,4}\,\mathrm{Y}_3^{0,2,3,4}}{\frac{\mathrm{Y}_3^{0,2,3,4}}{\mathrm{K_{mxy}}} + 1}
\end{aligned}$$

Layer Dynamics:

$$L(F^1|F^0,F^2,F^3,F^4) = \begin{bmatrix} X_1^1 & X_2^1 & X_3^1 & Y_1^1 & Y_2^1 & Y_3^1 \end{bmatrix}^T.$$

Layer $L(F^2|F^0)$

Required input trajectories:

$$\begin{bmatrix} Y_1^0 \\ Y_2^0 \\ Y_3^0 \end{bmatrix} = \begin{bmatrix} 0 & 0 & 0 & 1 & 0 & 0 \\ 0 & 0 & 0 & 0 & 1 & 0 \\ 0 & 0 & 0 & 0 & 0 & 1 \end{bmatrix} L(F^0).$$

Ordinary differential equations:

$$\frac{d}{dt}\begin{bmatrix} \mathrm{Y}_1^2 \\ \mathrm{Y}_2^2 \\ \mathrm{Y}_3^2 \end{bmatrix} = \begin{bmatrix} 1 & -1 & 0 \\ 0 & 1 & -1 \\ 0 & 0 & 1 \end{bmatrix} \begin{bmatrix} v_4 \\ v_5 \\ v_6 \end{bmatrix},$$

with

$$\begin{aligned} v_4 &= -\frac{\mathrm{S_2(t)\,k_y\,K_{my}\,C}\left(\mathrm{Y}_1^0 - \mathrm{Y_{1T}} + \mathrm{Y}_1^2\right)}{\mathrm{S_2(t)} + \mathrm{K_{my}}} \\ v_5 &= -\,\mathrm{k_{12y}\,C}\left(\mathrm{Y}_1^0 + \mathrm{Y}_1^2\right)\left(\mathrm{Y}_2^0 - \mathrm{Y_{2T}} + \mathrm{Y}_2^2\right) \\ v_6 &= -\,\mathrm{k_{23y}\,C}\left(\mathrm{Y}_2^0 + \mathrm{Y}_2^2\right)\left(\mathrm{Y}_3^0 - \mathrm{Y_{3T}} + \mathrm{Y}_3^2\right) \end{aligned}$$

Layer Dynamics:

$$L(F^2|F^0) = \begin{bmatrix} 0 & 0 & 0 & Y_1^2 & Y_2^2 & Y_3^2 \end{bmatrix}^T.$$

Layer $L(F^2|F^0,F^1)$

Required input trajectories:

$$\begin{bmatrix} Y_1^{0,1} \\ Y_2^{0,1} \\ Y_3^{0,1} \end{bmatrix} = \begin{bmatrix} 0 & 0 & 0 & 1 & 0 & 0 \\ 0 & 0 & 0 & 0 & 1 & 0 \\ 0 & 0 & 0 & 0 & 0 & 1 \end{bmatrix} L(F^0,F^1).$$

Ordinary differential equations:

$$\frac{d}{dt}\begin{bmatrix} \mathrm{Y}_1^2 \\ \mathrm{Y}_2^2 \\ \mathrm{Y}_3^2 \end{bmatrix} = \begin{bmatrix} 1 & -1 & 0 \\ 0 & 1 & -1 \\ 0 & 0 & 1 \end{bmatrix} \begin{bmatrix} v_4 \\ v_5 \\ v_6 \end{bmatrix},$$

with

$$\begin{aligned} v_4 &= -\frac{\mathrm{S_2(t)\,k_y\,K_{my}\,C}\left(\mathrm{Y}_1^{0,1} - \mathrm{Y_{1T}} + \mathrm{Y}_1^2\right)}{\mathrm{S_2(t)} + \mathrm{K_{my}}} \\ v_5 &= -\,\mathrm{k_{12y}\,C}\left(\mathrm{Y}_1^{0,1} + \mathrm{Y}_1^2\right)\left(\mathrm{Y}_2^{0,1} - \mathrm{Y_{2T}} + \mathrm{Y}_2^2\right) \end{aligned}$$

$$v_6 = -\,\mathrm{k_{23y}}\,\mathrm{C}\,\left(\mathrm{Y_2^{0,1}} + \mathrm{Y_2^2}\right)\,\left(\mathrm{Y_3^{0,1}} - \mathrm{Y_{3T}} + \mathrm{Y_3^2}\right)$$

Layer Dynamics:

$$L(F^2|F^0,F^1) = \begin{bmatrix}0 & 0 & 0 & Y_1^2 & Y_2^2 & Y_3^2\end{bmatrix}^T.$$

Layer $L(F^2|F^0,F^3)$

Required input trajectories:

$$\begin{bmatrix}X_3^{0,3}\\ Y_1^{0,3}\\ Y_2^{0,3}\\ Y_3^{0,3}\end{bmatrix} = \begin{bmatrix}0&0&1&0&0&0\\0&0&0&1&0&0\\0&0&0&0&1&0\\0&0&0&0&0&1\end{bmatrix} L(F^0,F^3).$$

Ordinary differential equations:

$$\frac{d}{dt}\begin{bmatrix}X_3^2\\ Y_1^2\\ Y_2^2\\ Y_3^2\end{bmatrix} = \begin{bmatrix}0&0&0\\1&-1&0\\0&1&-1\\0&0&1\end{bmatrix}\begin{bmatrix}v_4\\ v_5\\ v_6\end{bmatrix} + \begin{bmatrix}-1&0\\0&0\\0&0\\0&-1\end{bmatrix}\begin{bmatrix}v_{alt,9}\\ v_{alt,10}\end{bmatrix},$$

with

$$\begin{aligned}
v_4 &= -\,\frac{\mathrm{S_2(t)}\,\mathrm{k_y}\,\mathrm{K_{my}}\,\mathrm{C}\,\left(\mathrm{Y_1^{0,3}} - \mathrm{Y_{1T}} + \mathrm{Y_1^2}\right)}{\mathrm{S_2(t)} + \mathrm{K_{my}}}\\
v_5 &= -\,\mathrm{k_{12y}}\,\mathrm{C}\,\left(\mathrm{Y_1^{0,3}} + \mathrm{Y_1^2}\right)\,\left(\mathrm{Y_2^{0,3}} - \mathrm{Y_{2T}} + \mathrm{Y_2^2}\right)\\
v_6 &= -\,\mathrm{k_{23y}}\,\mathrm{C}\,\left(\mathrm{Y_2^{0,3}} + \mathrm{Y_2^2}\right)\,\left(\mathrm{Y_3^{0,3}} - \mathrm{Y_{3T}} + \mathrm{Y_3^2}\right)\\
v_{alt,9} &= \frac{\mathrm{k_d}\,\mathrm{C}\,\left(\mathrm{X_3^{0,3}} + \mathrm{X_3^2}\right)\,\left(\mathrm{Y_3^{0,3}} + \mathrm{Y_3^2}\right)}{\frac{\mathrm{X_3^{0,3}}+\mathrm{X_3^2}}{\mathrm{K_{myx}}} + 1} - \frac{\mathrm{k_d}\,\mathrm{C}\,\mathrm{X_3^{0,3}}\,\mathrm{Y_3^{0,3}}}{\frac{\mathrm{X_3^{0,3}}}{\mathrm{K_{myx}}} + 1}\\
v_{alt,10} &= \frac{\mathrm{k_d}\,\mathrm{C}\,\left(\mathrm{X_3^{0,3}} + \mathrm{X_3^2}\right)\,\left(\mathrm{Y_3^{0,3}} + \mathrm{Y_3^2}\right)}{\frac{\mathrm{Y_3^{0,3}}+\mathrm{Y_3^2}}{\mathrm{K_{mxy}}} + 1} - \frac{\mathrm{k_d}\,\mathrm{C}\,\mathrm{X_3^{0,3}}\,\mathrm{Y_3^{0,3}}}{\frac{\mathrm{Y_3^{0,3}}}{\mathrm{K_{mxy}}} + 1}
\end{aligned}$$

Layer Dynamics:

$$L(F^2|F^0,F^3) = \begin{bmatrix}0 & 0 & X_3^2 & Y_1^2 & Y_2^2 & Y_3^2\end{bmatrix}^T.$$

Layer $L(F^2|F^0,F^4)$

Required input trajectories:

$$\begin{bmatrix}X_1^{0,4}\\ X_2^{0,4}\\ Y_1^{0,4}\\ Y_2^{0,4}\\ Y_3^{0,4}\end{bmatrix} = \begin{bmatrix}1&0&0&0&0&0\\0&1&0&0&0&0\\0&0&0&1&0&0\\0&0&0&0&1&0\\0&0&0&0&0&1\end{bmatrix} L(F^0,F^4).$$

Ordinary differential equations:

$$\frac{d}{dt}\begin{bmatrix}X_1^2\\X_2^2\\Y_1^2\\Y_2^2\\Y_3^2\end{bmatrix}=\begin{bmatrix}0&0&0\\0&0&0\\1&-1&0\\0&1&-1\\0&0&1\end{bmatrix}\begin{bmatrix}v_4\\v_5\\v_6\end{bmatrix}+\begin{bmatrix}-1&0\\0&1\\0&-1\\1&0\\0&0\end{bmatrix}\begin{bmatrix}v_{alt,7}\\v_{alt,8}\end{bmatrix},$$

with

$$\begin{aligned}
v_4 &= -\frac{\mathrm{S_2(t)\,k_y\,K_{my}\,C}\left(\mathrm{Y_1^{0,4}-Y_{1T}+Y_1^2}\right)}{\mathrm{S_2(t)+K_{my}}}\\
v_5 &= -\mathrm{k_{12y}\,C}\left(\mathrm{Y_1^{0,4}+Y_1^2}\right)\left(\mathrm{Y_2^{0,4}-Y_{2T}+Y_2^2}\right)\\
v_6 &= -\mathrm{k_{23y}\,C}\left(\mathrm{Y_2^{0,4}+Y_2^2}\right)\left(\mathrm{Y_3^{0,4}-Y_{3T}+Y_3^2}\right)\\
v_{alt,7} &= -\mathrm{k_a\,C\,X_1^{0,4}\,Y_2^2-k_a\,C\,X_1^2}\left(\mathrm{Y_2^{0,4}-Y_{2T}+Y_2^2}\right)\\
v_{alt,8} &= -\mathrm{k_a\,C\,X_2^2\,Y_1^{0,4}-k_a\,C\,Y_1^2}\left(\mathrm{X_2^{0,4}-X_{2T}+X_2^2}\right)
\end{aligned}$$

Layer Dynamics:

$$L(F^2|F^0,F^4)=\begin{bmatrix}X_1^2 & X_2^2 & 0 & Y_1^2 & Y_2^2 & Y_3^2\end{bmatrix}^T.$$

Layer $L(F^2|F^0,F^1,F^3)$

Required input trajectories:

$$\begin{bmatrix}X_1^{0,1,3}\\X_2^{0,1,3}\\X_3^{0,1,3}\\Y_1^{0,1,3}\\Y_2^{0,1,3}\\Y_3^{0,1,3}\end{bmatrix}=\begin{bmatrix}1&0&0&0&0&0\\0&1&0&0&0&0\\0&0&1&0&0&0\\0&0&0&1&0&0\\0&0&0&0&1&0\\0&0&0&0&0&1\end{bmatrix}L(F^0,F^1,F^3).$$

Ordinary differential equations:

$$\frac{d}{dt}\begin{bmatrix}X_1^2\\X_2^2\\X_3^2\\Y_1^2\\Y_2^2\\Y_3^2\end{bmatrix}=\begin{bmatrix}0&0&0\\0&0&0\\0&0&0\\1&-1&0\\0&1&-1\\0&0&1\end{bmatrix}\begin{bmatrix}v_4\\v_5\\v_6\end{bmatrix}+\begin{bmatrix}-1&1&0&0&0\\1&0&-1&0&0\\0&0&1&-1&0\\0&0&0&0&0\\0&0&0&0&0\\0&0&0&0&-1\end{bmatrix}\begin{bmatrix}v_{alt,1}\\v_{alt,2}\\v_{alt,3}\\v_{alt,9}\\v_{alt,10}\end{bmatrix},$$

with

$$\begin{aligned}
v_4 &= -\frac{\mathrm{S_2(t)\,k_y\,K_{my}\,C}\left(\mathrm{Y_1^{0,1,3}-Y_{1T}+Y_1^2}\right)}{\mathrm{S_2(t)+K_{my}}}\\
v_5 &= -\mathrm{k_{12y}\,C}\left(\mathrm{Y_1^{0,1,3}+Y_1^2}\right)\left(\mathrm{Y_2^{0,1,3}-Y_{2T}+Y_2^2}\right)
\end{aligned}$$

$$
\begin{aligned}
v_6 =& -\mathrm{k_{23y}}\,\mathrm{C}\,\left(\mathrm{Y_2^{0,1,3}}+\mathrm{Y_2^2}\right)\left(\mathrm{Y_3^{0,1,3}}-\mathrm{Y_{3T}}+\mathrm{Y_3^2}\right)\\
v_{alt,1} =& -\mathrm{k_{12x}}\,\mathrm{C}\,\mathrm{X_1^{0,1,3}}\,\mathrm{X_2^2}-\mathrm{k_{12x}}\,\mathrm{C}\,\mathrm{X_1^2}\left(\mathrm{X_2^{0,1,3}}-\mathrm{X_{2T}}+\mathrm{X_2^2}\right)\\
v_{alt,2} =& -\frac{\mathrm{C}\,\mathrm{k_x}\,\mathrm{S_1(t)}\,\mathrm{K_{mx}}\,\mathrm{X_1^2}}{\mathrm{S_1(t)}+\mathrm{K_{mx}}}\\
v_{alt,3} =& -\mathrm{k_{23x}}\,\mathrm{C}\,\mathrm{X_2^{0,1,3}}\,\mathrm{X_3^2}-\mathrm{k_{23x}}\,\mathrm{C}\,\mathrm{X_2^2}\left(\mathrm{X_3^{0,1,3}}-\mathrm{X_{3T}}+\mathrm{X_3^2}\right)\\
v_{alt,9} =& \frac{\mathrm{k_d}\,\mathrm{C}\left(\mathrm{X_3^{0,1,3}}+\mathrm{X_3^2}\right)\left(\mathrm{Y_3^{0,1,3}}+\mathrm{Y_3^2}\right)}{\frac{\mathrm{X_3^{0,1,3}}+\mathrm{X_3^2}}{\mathrm{K_{myx}}}+1}-\frac{\mathrm{k_d}\,\mathrm{C}\,\mathrm{X_3^{0,1,3}}\,\mathrm{Y_3^{0,1,3}}}{\frac{\mathrm{X_3^{0,1,3}}}{\mathrm{K_{myx}}}+1}\\
v_{alt,10} =& \frac{\mathrm{k_d}\,\mathrm{C}\left(\mathrm{X_3^{0,1,3}}+\mathrm{X_3^2}\right)\left(\mathrm{Y_3^{0,1,3}}+\mathrm{Y_3^2}\right)}{\frac{\mathrm{Y_3^{0,1,3}}+\mathrm{Y_3^2}}{\mathrm{K_{mxy}}}+1}-\frac{\mathrm{k_d}\,\mathrm{C}\,\mathrm{X_3^{0,1,3}}\,\mathrm{Y_3^{0,1,3}}}{\frac{\mathrm{Y_3^{0,1,3}}}{\mathrm{K_{mxy}}}+1}
\end{aligned}
$$

Layer Dynamics:

$$L(F^2|F^0,F^1,F^3)=\begin{bmatrix}X_1^2 & X_2^2 & X_3^2 & Y_1^2 & Y_2^2 & Y_3^2\end{bmatrix}^T.$$

Layer $L(F^2|F^0,F^1,F^4)$

Required input trajectories:

$$\begin{bmatrix}X_1^{0,1,4}\\X_2^{0,1,4}\\X_3^{0,1,4}\\Y_1^{0,1,4}\\Y_2^{0,1,4}\\Y_3^{0,1,4}\end{bmatrix}=\begin{bmatrix}1&0&0&0&0&0\\0&1&0&0&0&0\\0&0&1&0&0&0\\0&0&0&1&0&0\\0&0&0&0&1&0\\0&0&0&0&0&1\end{bmatrix}L(F^0,F^1,F^4).$$

Ordinary differential equations:

$$\frac{d}{dt}\begin{bmatrix}X_1^2\\X_2^2\\X_3^2\\Y_1^2\\Y_2^2\\Y_3^2\end{bmatrix}=\begin{bmatrix}0&0&0\\0&0&0\\0&0&0\\1&-1&0\\0&1&-1\\0&0&1\end{bmatrix}\begin{bmatrix}v_4\\v_5\\v_6\end{bmatrix}+\begin{bmatrix}-1&1&0&-1&0\\1&0&-1&0&1\\0&0&1&0&0\\0&0&0&0&-1\\0&0&0&1&0\\0&0&0&0&0\end{bmatrix}\begin{bmatrix}v_{alt,1}\\v_{alt,2}\\v_{alt,3}\\v_{alt,7}\\v_{alt,8}\end{bmatrix},$$

with

$$
\begin{aligned}
v_4 =& -\frac{\mathrm{S_2(t)}\,\mathrm{k_y}\,\mathrm{K_{my}}\,\mathrm{C}\left(\mathrm{Y_1^{0,1,4}}-\mathrm{Y_{1T}}+\mathrm{Y_1^2}\right)}{\mathrm{S_2(t)}+\mathrm{K_{my}}}\\
v_5 =& -\mathrm{k_{12y}}\,\mathrm{C}\left(\mathrm{Y_1^{0,1,4}}+\mathrm{Y_1^2}\right)\left(\mathrm{Y_2^{0,1,4}}-\mathrm{Y_{2T}}+\mathrm{Y_2^2}\right)\\
v_6 =& -\mathrm{k_{23y}}\,\mathrm{C}\left(\mathrm{Y_2^{0,1,4}}+\mathrm{Y_2^2}\right)\left(\mathrm{Y_3^{0,1,4}}-\mathrm{Y_{3T}}+\mathrm{Y_3^2}\right)\\
v_{alt,1} =& -\mathrm{k_{12x}}\,\mathrm{C}\,\mathrm{X_1^{0,1,4}}\,\mathrm{X_2^2}-\mathrm{k_{12x}}\,\mathrm{C}\,\mathrm{X_1^2}\left(\mathrm{X_2^{0,1,4}}-\mathrm{X_{2T}}+\mathrm{X_2^2}\right)
\end{aligned}
$$

$$
\begin{aligned}
v_{alt,2} &= -\frac{\mathrm{C}\,\mathrm{k_x}\,\mathrm{S_1(t)}\,\mathrm{K_{mx}}\,\mathrm{X_1^2}}{\mathrm{S_1(t)}+\mathrm{K_{mx}}}\\
v_{alt,3} &= -\mathrm{k_{23x}}\,\mathrm{C}\,\mathrm{X_2^{0,1,4}}\,\mathrm{X_3^2} - \mathrm{k_{23x}}\,\mathrm{C}\,\mathrm{X_2^2}\left(\mathrm{X_3^{0,1,4}} - \mathrm{X_{3T}} + \mathrm{X_3^2}\right)\\
v_{alt,7} &= -\mathrm{k_a}\,\mathrm{C}\,\mathrm{X_1^{0,1,4}}\,\mathrm{Y_2^2} - \mathrm{k_a}\,\mathrm{C}\,\mathrm{X_1^2}\left(\mathrm{Y_2^{0,1,4}} - \mathrm{Y_{2T}} + \mathrm{Y_2^2}\right)\\
v_{alt,8} &= -\mathrm{k_a}\,\mathrm{C}\,\mathrm{X_2^2}\,\mathrm{Y_1^{0,1,4}} - \mathrm{k_a}\,\mathrm{C}\,\mathrm{Y_1^2}\left(\mathrm{X_2^{0,1,4}} - \mathrm{X_{2T}} + \mathrm{X_2^2}\right)
\end{aligned}
$$

Layer Dynamics:

$$
L(F^2|F^0,F^1,F^4) = \begin{bmatrix} X_1^2 & X_2^2 & X_3^2 & Y_1^2 & Y_2^2 & Y_3^2 \end{bmatrix}^T .
$$

Layer $L(F^2|F^0,F^3,F^4)$

Required input trajectories:

$$
\begin{bmatrix} X_1^{0,3,4}\\ X_2^{0,3,4}\\ X_3^{0,3,4}\\ Y_1^{0,3,4}\\ Y_2^{0,3,4}\\ Y_3^{0,3,4} \end{bmatrix} = \begin{bmatrix} 1&0&0&0&0&0\\ 0&1&0&0&0&0\\ 0&0&1&0&0&0\\ 0&0&0&1&0&0\\ 0&0&0&0&1&0\\ 0&0&0&0&0&1 \end{bmatrix} L(F^0,F^3,F^4).
$$

Ordinary differential equations:

$$
\frac{d}{dt}\begin{bmatrix} X_1^2\\ X_2^2\\ X_3^2\\ Y_1^2\\ Y_2^2\\ Y_3^2 \end{bmatrix} = \begin{bmatrix} 0&0&0\\ 0&0&0\\ 0&0&0\\ 1&-1&0\\ 0&1&-1\\ 0&0&1 \end{bmatrix}\begin{bmatrix} v_4\\ v_5\\ v_6 \end{bmatrix} + \begin{bmatrix} 0&0&-1&0\\ 0&0&0&1\\ -1&0&0&0\\ 0&0&0&-1\\ 0&0&1&0\\ 0&-1&0&0 \end{bmatrix}\begin{bmatrix} v_{alt,9}\\ v_{alt,10}\\ v_{alt,7}\\ v_{alt,8} \end{bmatrix},
$$

with

$$
\begin{aligned}
v_4 &= -\frac{\mathrm{S_2(t)}\,\mathrm{k_y}\,\mathrm{K_{my}}\,\mathrm{C}\left(\mathrm{Y_1^{0,3,4}} - \mathrm{Y_{1T}} + \mathrm{Y_1^2}\right)}{\mathrm{S_2(t)}+\mathrm{K_{my}}}\\
v_5 &= -\mathrm{k_{12y}}\,\mathrm{C}\left(\mathrm{Y_1^{0,3,4}} + \mathrm{Y_1^2}\right)\left(\mathrm{Y_2^{0,3,4}} - \mathrm{Y_{2T}} + \mathrm{Y_2^2}\right)\\
v_6 &= -\mathrm{k_{23y}}\,\mathrm{C}\left(\mathrm{Y_2^{0,3,4}} + \mathrm{Y_2^2}\right)\left(\mathrm{Y_3^{0,3,4}} - \mathrm{Y_{3T}} + \mathrm{Y_3^2}\right)\\
v_{alt,9} &= \frac{\mathrm{k_d}\,\mathrm{C}\left(\mathrm{X_3^{0,3,4}} + \mathrm{X_3^2}\right)\left(\mathrm{Y_3^{0,3,4}} + \mathrm{Y_3^2}\right)}{\frac{\mathrm{X_3^{0,3,4}}+\mathrm{X_3^2}}{\mathrm{K_{myx}}}+1} - \frac{\mathrm{k_d}\,\mathrm{C}\,\mathrm{X_3^{0,3,4}}\,\mathrm{Y_3^{0,3,4}}}{\frac{\mathrm{X_3^{0,3,4}}}{\mathrm{K_{myx}}}+1}\\
v_{alt,10} &= \frac{\mathrm{k_d}\,\mathrm{C}\left(\mathrm{X_3^{0,3,4}} + \mathrm{X_3^2}\right)\left(\mathrm{Y_3^{0,3,4}} + \mathrm{Y_3^2}\right)}{\frac{\mathrm{Y_3^{0,3,4}}+\mathrm{Y_3^2}}{\mathrm{K_{mxy}}}+1} - \frac{\mathrm{k_d}\,\mathrm{C}\,\mathrm{X_3^{0,3,4}}\,\mathrm{Y_3^{0,3,4}}}{\frac{\mathrm{Y_3^{0,3,4}}}{\mathrm{K_{mxy}}}+1}\\
v_{alt,7} &= -\mathrm{k_a}\,\mathrm{C}\,\mathrm{X_1^{0,3,4}}\,\mathrm{Y_2^2} - \mathrm{k_a}\,\mathrm{C}\,\mathrm{X_1^2}\left(\mathrm{Y_2^{0,3,4}} - \mathrm{Y_{2T}} + \mathrm{Y_2^2}\right)
\end{aligned}
$$

$$v_{alt,8} = -\,\mathrm{k_a}\,\mathrm{C}\,\mathrm{X}_2^2\,\mathrm{Y}_1^{0,3,4} - \mathrm{k_a}\,\mathrm{C}\,\mathrm{Y}_1^2\,\left(\mathrm{X}_2^{0,3,4} - \mathrm{X_{2T}} + \mathrm{X}_2^2\right)$$

Layer Dynamics:

$$L(F^2|F^0, F^3, F^4) = \begin{bmatrix} X_1^2 & X_2^2 & X_3^2 & Y_1^2 & Y_2^2 & Y_3^2 \end{bmatrix}^T.$$

Layer $L(F^2|F^0, F^1, F^3, F^4)$

Required input trajectories:

$$\begin{bmatrix} X_1^{0,1,3,4} \\ X_2^{0,1,3,4} \\ X_3^{0,1,3,4} \\ Y_1^{0,1,3,4} \\ Y_2^{0,1,3,4} \\ Y_3^{0,1,3,4} \end{bmatrix} = \begin{bmatrix} 1 & 0 & 0 & 0 & 0 & 0 \\ 0 & 1 & 0 & 0 & 0 & 0 \\ 0 & 0 & 1 & 0 & 0 & 0 \\ 0 & 0 & 0 & 1 & 0 & 0 \\ 0 & 0 & 0 & 0 & 1 & 0 \\ 0 & 0 & 0 & 0 & 0 & 1 \end{bmatrix} L(F^0, F^1, F^3, F^4).$$

Ordinary differential equations:

$$\frac{d}{dt}\begin{bmatrix} X_1^2 \\ X_2^2 \\ X_3^2 \\ Y_1^2 \\ Y_2^2 \\ Y_3^2 \end{bmatrix} = \begin{bmatrix} 0 & 0 & 0 \\ 0 & 0 & 0 \\ 0 & 0 & 0 \\ 1 & -1 & 0 \\ 0 & 1 & -1 \\ 0 & 0 & 1 \end{bmatrix} \begin{bmatrix} v_4 \\ v_5 \\ v_6 \end{bmatrix} + \begin{bmatrix} -1 & 1 & 0 & -1 & 0 & 0 & 0 \\ 1 & 0 & -1 & 0 & 1 & 0 & 0 \\ 0 & 0 & 1 & 0 & 0 & -1 & 0 \\ 0 & 0 & 0 & 0 & -1 & 0 & 0 \\ 0 & 0 & 0 & 1 & 0 & 0 & 0 \\ 0 & 0 & 0 & 0 & 0 & 0 & -1 \end{bmatrix} \begin{bmatrix} v_{alt,1} \\ v_{alt,2} \\ v_{alt,3} \\ v_{alt,7} \\ v_{alt,8} \\ v_{alt,9} \\ v_{alt,10} \end{bmatrix},$$

with

$$\begin{aligned}
v_4 &= -\,\frac{\mathrm{S_2(t)}\,\mathrm{k_y}\,\mathrm{K_{my}}\,\mathrm{C}\,\left(\mathrm{Y}_1^{0,1,3,4} - \mathrm{Y_{1T}} + \mathrm{Y}_1^2\right)}{\mathrm{S_2(t)} + \mathrm{K_{my}}} \\
v_5 &= -\,\mathrm{k_{12y}}\,\mathrm{C}\,\left(\mathrm{Y}_1^{0,1,3,4} + \mathrm{Y}_1^2\right)\left(\mathrm{Y}_2^{0,1,3,4} - \mathrm{Y_{2T}} + \mathrm{Y}_2^2\right) \\
v_6 &= -\,\mathrm{k_{23y}}\,\mathrm{C}\,\left(\mathrm{Y}_2^{0,1,3,4} + \mathrm{Y}_2^2\right)\left(\mathrm{Y}_3^{0,1,3,4} - \mathrm{Y_{3T}} + \mathrm{Y}_3^2\right) \\
v_{alt,1} &= -\,\mathrm{k_{12x}}\,\mathrm{C}\,\mathrm{X}_1^{0,1,3,4}\,\mathrm{X}_2^2 - \mathrm{k_{12x}}\,\mathrm{C}\,\mathrm{X}_1^2\,\left(\mathrm{X}_2^{0,1,3,4} - \mathrm{X_{2T}} + \mathrm{X}_2^2\right) \\
v_{alt,2} &= -\,\frac{\mathrm{C}\,\mathrm{k_x}\,\mathrm{S_1(t)}\,\mathrm{K_{mx}}\,\mathrm{X}_1^2}{\mathrm{S_1(t)} + \mathrm{K_{mx}}} \\
v_{alt,3} &= -\,\mathrm{k_{23x}}\,\mathrm{C}\,\mathrm{X}_2^{0,1,3,4}\,\mathrm{X}_3^2 - \mathrm{k_{23x}}\,\mathrm{C}\,\mathrm{X}_2^2\,\left(\mathrm{X}_3^{0,1,3,4} - \mathrm{X_{3T}} + \mathrm{X}_3^2\right) \\
v_{alt,7} &= -\,\mathrm{k_a}\,\mathrm{C}\,\mathrm{X}_1^{0,1,3,4}\,\mathrm{Y}_2^2 - \mathrm{k_a}\,\mathrm{C}\,\mathrm{X}_1^2\,\left(\mathrm{Y}_2^{0,1,3,4} - \mathrm{Y_{2T}} + \mathrm{Y}_2^2\right) \\
v_{alt,8} &= -\,\mathrm{k_a}\,\mathrm{C}\,\mathrm{X}_2^2\,\mathrm{Y}_1^{0,1,3,4} - \mathrm{k_a}\,\mathrm{C}\,\mathrm{Y}_1^2\,\left(\mathrm{X}_2^{0,1,3,4} - \mathrm{X_{2T}} + \mathrm{X}_2^2\right) \\
v_{alt,9} &= \frac{\mathrm{k_d}\,\mathrm{C}\,\left(\mathrm{X}_3^{0,1,3,4} + \mathrm{X}_3^2\right)\left(\mathrm{Y}_3^{0,1,3,4} + \mathrm{Y}_3^2\right)}{\frac{\mathrm{X}_3^{0,1,3,4} + \mathrm{X}_3^2}{\mathrm{K_{myx}}} + 1} - \frac{\mathrm{k_d}\,\mathrm{C}\,\mathrm{X}_3^{0,1,3,4}\,\mathrm{Y}_3^{0,1,3,4}}{\frac{\mathrm{X}_3^{0,1,3,4}}{\mathrm{K_{myx}}} + 1}
\end{aligned}$$

$$v_{alt,10} = \frac{\mathrm{k_d\, C}\left(\mathrm{X}_3^{0,1,3,4} + \mathrm{X}_3^2\right)\left(\mathrm{Y}_3^{0,1,3,4} + \mathrm{Y}_3^2\right)}{\frac{\mathrm{Y}_3^{0,1,3,4} + \mathrm{Y}_3^2}{\mathrm{K_{mxy}}} + 1} - \frac{\mathrm{k_d\, C\, X}_3^{0,1,3,4}\,\mathrm{Y}_3^{0,1,3,4}}{\frac{\mathrm{Y}_3^{0,1,3,4}}{\mathrm{K_{mxy}}} + 1}$$

Layer Dynamics:

$$L(F^2|F^0, F^1, F^3, F^4) = \begin{bmatrix} X_1^2 & X_2^2 & X_3^2 & Y_1^2 & Y_2^2 & Y_3^2 \end{bmatrix}^T.$$

Layer $L(F^3|F^0)$

Required input trajectories:

$$\begin{bmatrix} X_3^0 \\ Y_3^0 \end{bmatrix} = \begin{bmatrix} 0 & 0 & 1 & 0 & 0 & 0 \\ 0 & 0 & 0 & 0 & 0 & 1 \end{bmatrix} L(F^0).$$

Ordinary differential equations:

$$\frac{d}{dt}\begin{bmatrix} X_3^3 \\ Y_3^3 \end{bmatrix} = \begin{bmatrix} -1 & 0 \\ 0 & -1 \end{bmatrix}\begin{bmatrix} v_9 \\ v_{10} \end{bmatrix},$$

with

$$v_9 = \frac{\mathrm{k_d\, K_{myx}\, C}\left(\mathrm{X}_3^0 + \mathrm{X}_3^3\right)\left(\mathrm{Y}_3^0 + \mathrm{Y}_3^3\right)}{\mathrm{K_{myx}} + \mathrm{X}_3^0 + \mathrm{X}_3^3}$$

$$v_{10} = \frac{\mathrm{k_d\, K_{mxy}\, C}\left(\mathrm{X}_3^0 + \mathrm{X}_3^3\right)\left(\mathrm{Y}_3^0 + \mathrm{Y}_3^3\right)}{\mathrm{K_{mxy}} + \mathrm{Y}_3^0 + \mathrm{Y}_3^3}$$

Layer Dynamics:

$$L(F^3|F^0) = \begin{bmatrix} 0 & 0 & X_3^3 & 0 & 0 & Y_3^3 \end{bmatrix}^T.$$

Layer $L(F^3|F^0, F^1)$

Required input trajectories:

$$\begin{bmatrix} X_1^{0,1} \\ X_2^{0,1} \\ X_3^{0,1} \\ Y_3^{0,1} \end{bmatrix} = \begin{bmatrix} 1 & 0 & 0 & 0 & 0 & 0 \\ 0 & 1 & 0 & 0 & 0 & 0 \\ 0 & 0 & 1 & 0 & 0 & 0 \\ 0 & 0 & 0 & 0 & 0 & 1 \end{bmatrix} L(F^0, F^1).$$

Ordinary differential equations:

$$\frac{d}{dt}\begin{bmatrix} X_1^3 \\ X_2^3 \\ X_3^3 \\ Y_3^3 \end{bmatrix} = \begin{bmatrix} 0 & 0 \\ 0 & 0 \\ -1 & 0 \\ 0 & -1 \end{bmatrix}\begin{bmatrix} v_9 \\ v_{10} \end{bmatrix} + \begin{bmatrix} -1 & 1 & 0 \\ 1 & 0 & -1 \\ 0 & 0 & 1 \\ 0 & 0 & 0 \end{bmatrix}\begin{bmatrix} v_{alt,1} \\ v_{alt,2} \\ v_{alt,3} \end{bmatrix},$$

with

$$v_9 = \frac{\mathrm{k_d\, K_{myx}\, C}\left(\mathrm{X}_3^{0,1} + \mathrm{X}_3^3\right)\left(\mathrm{Y}_3^{0,1} + \mathrm{Y}_3^3\right)}{\mathrm{K_{myx}} + \mathrm{X}_3^{0,1} + \mathrm{X}_3^3}$$

$$
\begin{aligned}
v_{10} =& \frac{\mathrm{k_d\,K_{mxy}\,C}\,\left(\mathrm{X_3^{0,1}} + \mathrm{X_3^3}\right)\,\left(\mathrm{Y_3^{0,1}} + \mathrm{Y_3^3}\right)}{\mathrm{K_{mxy}} + \mathrm{Y_3^{0,1}} + \mathrm{Y_3^3}} \\
v_{alt,1} =& -\mathrm{k_{12x}\,C\,X_1^{0,1}\,X_2^3} - \mathrm{k_{12x}\,C\,X_1^3}\,\left(\mathrm{X_2^{0,1}} - \mathrm{X_{2T}} + \mathrm{X_2^3}\right) \\
v_{alt,2} =& -\frac{\mathrm{C\,k_x\,S_1(t)\,K_{mx}\,X_1^3}}{\mathrm{S_1(t)} + \mathrm{K_{mx}}} \\
v_{alt,3} =& -\mathrm{k_{23x}\,C\,X_2^{0,1}\,X_3^3} - \mathrm{k_{23x}\,C\,X_2^3}\,\left(\mathrm{X_3^{0,1}} - \mathrm{X_{3T}} + \mathrm{X_3^3}\right)
\end{aligned}
$$

Layer Dynamics:

$$
L(F^3|F^0,F^1) = \begin{bmatrix} X_1^3 & X_2^3 & X_3^3 & 0 & 0 & Y_3^3 \end{bmatrix}^T.
$$

Layer $L(F^3|F^0,F^2)$

Required input trajectories:

$$
\begin{bmatrix} X_3^{0,2} \\ Y_1^{0,2} \\ Y_2^{0,2} \\ Y_3^{0,2} \end{bmatrix} = \begin{bmatrix} 0 & 0 & 1 & 0 & 0 & 0 \\ 0 & 0 & 0 & 1 & 0 & 0 \\ 0 & 0 & 0 & 0 & 1 & 0 \\ 0 & 0 & 0 & 0 & 0 & 1 \end{bmatrix} L(F^0,F^2).
$$

Ordinary differential equations:

$$
\frac{d}{dt}\begin{bmatrix} X_3^3 \\ Y_1^3 \\ Y_2^3 \\ Y_3^3 \end{bmatrix} = \begin{bmatrix} -1 & 0 \\ 0 & 0 \\ 0 & 0 \\ 0 & -1 \end{bmatrix} \begin{bmatrix} v_9 \\ v_{10} \end{bmatrix} + \begin{bmatrix} 0 & 0 & 0 \\ 1 & -1 & 0 \\ 0 & 1 & -1 \\ 0 & 0 & 1 \end{bmatrix} \begin{bmatrix} v_{alt,4} \\ v_{alt,5} \\ v_{alt,6} \end{bmatrix},
$$

with

$$
\begin{aligned}
v_9 =& \frac{\mathrm{k_d\,K_{myx}\,C}\,\left(\mathrm{X_3^{0,2}} + \mathrm{X_3^3}\right)\,\left(\mathrm{Y_3^{0,2}} + \mathrm{Y_3^3}\right)}{\mathrm{K_{myx}} + \mathrm{X_3^{0,2}} + \mathrm{X_3^3}} \\
v_{10} =& \frac{\mathrm{k_d\,K_{mxy}\,C}\,\left(\mathrm{X_3^{0,2}} + \mathrm{X_3^3}\right)\,\left(\mathrm{Y_3^{0,2}} + \mathrm{Y_3^3}\right)}{\mathrm{K_{mxy}} + \mathrm{Y_3^{0,2}} + \mathrm{Y_3^3}} \\
v_{alt,4} =& -\frac{\mathrm{S_2(t)\,k_y\,K_{my}\,C\,Y_1^3}}{\mathrm{S_2(t)} + \mathrm{K_{my}}} \\
v_{alt,5} =& -\mathrm{k_{12y}\,C\,Y_1^{0,2}\,Y_2^3} - \mathrm{k_{12y}\,C\,Y_1^3}\,\left(\mathrm{Y_2^{0,2}} - \mathrm{Y_{2T}} + \mathrm{Y_2^3}\right) \\
v_{alt,6} =& -\mathrm{k_{23y}\,C\,Y_2^{0,2}\,Y_3^3} - \mathrm{k_{23y}\,C\,Y_2^3}\,\left(\mathrm{Y_3^{0,2}} - \mathrm{Y_{3T}} + \mathrm{Y_3^3}\right)
\end{aligned}
$$

Layer Dynamics:

$$
L(F^3|F^0,F^2) = \begin{bmatrix} 0 & 0 & X_3^3 & Y_1^3 & Y_2^3 & Y_3^3 \end{bmatrix}^T.
$$

Layer $L(F^3|F^0,F^4)$

Required input trajectories:

$$\begin{bmatrix} X_3^{0,4} \\ Y_3^{0,4} \end{bmatrix} = \begin{bmatrix} 0 & 0 & 1 & 0 & 0 & 0 \\ 0 & 0 & 0 & 0 & 0 & 1 \end{bmatrix} L(F^0,F^4).$$

Ordinary differential equations:

$$\frac{d}{dt}\begin{bmatrix} X_3^3 \\ Y_3^3 \end{bmatrix} = \begin{bmatrix} -1 & 0 \\ 0 & -1 \end{bmatrix} \begin{bmatrix} v_9 \\ v_{10} \end{bmatrix},$$

with

$$\begin{aligned} v_9 &= \frac{\mathrm{k_d\,K_{myx}\,C}\left(\mathrm{X}_3^{0,4}+\mathrm{X}_3^3\right)\left(\mathrm{Y}_3^{0,4}+\mathrm{Y}_3^3\right)}{\mathrm{K_{myx}}+\mathrm{X}_3^{0,4}+\mathrm{X}_3^3} \\ v_{10} &= \frac{\mathrm{k_d\,K_{mxy}\,C}\left(\mathrm{X}_3^{0,4}+\mathrm{X}_3^3\right)\left(\mathrm{Y}_3^{0,4}+\mathrm{Y}_3^3\right)}{\mathrm{K_{mxy}}+\mathrm{Y}_3^{0,4}+\mathrm{Y}_3^3} \end{aligned}$$

Layer Dynamics:

$$L(F^3|F^0,F^4) = \begin{bmatrix} 0 & 0 & X_3^3 & 0 & 0 & Y_3^3 \end{bmatrix}^T.$$

Layer $L(F^3|F^0,F^1,F^2)$

Required input trajectories:

$$\begin{bmatrix} X_1^{0,1,2} \\ X_2^{0,1,2} \\ X_3^{0,1,2} \\ Y_1^{0,1,2} \\ Y_2^{0,1,2} \\ Y_3^{0,1,2} \end{bmatrix} = \begin{bmatrix} 1 & 0 & 0 & 0 & 0 & 0 \\ 0 & 1 & 0 & 0 & 0 & 0 \\ 0 & 0 & 1 & 0 & 0 & 0 \\ 0 & 0 & 0 & 1 & 0 & 0 \\ 0 & 0 & 0 & 0 & 1 & 0 \\ 0 & 0 & 0 & 0 & 0 & 1 \end{bmatrix} L(F^0,F^1,F^2).$$

Ordinary differential equations:

$$\frac{d}{dt}\begin{bmatrix} X_1^3 \\ X_2^3 \\ X_3^3 \\ Y_1^3 \\ Y_2^3 \\ Y_3^3 \end{bmatrix} = \begin{bmatrix} 0 & 0 \\ 0 & 0 \\ -1 & 0 \\ 0 & 0 \\ 0 & 0 \\ 0 & -1 \end{bmatrix} \begin{bmatrix} v_9 \\ v_{10} \end{bmatrix} + \begin{bmatrix} -1 & 1 & 0 & 0 & 0 & 0 \\ 1 & 0 & -1 & 0 & 0 & 0 \\ 0 & 0 & 1 & 0 & 0 & 0 \\ 0 & 0 & 0 & 1 & -1 & 0 \\ 0 & 0 & 0 & 0 & 1 & -1 \\ 0 & 0 & 0 & 0 & 0 & 1 \end{bmatrix} \begin{bmatrix} v_{alt,1} \\ v_{alt,2} \\ v_{alt,3} \\ v_{alt,4} \\ v_{alt,5} \\ v_{alt,6} \end{bmatrix},$$

with

$$\begin{aligned} v_9 &= \frac{\mathrm{k_d\,K_{myx}\,C}\left(\mathrm{X}_3^{0,1,2}+\mathrm{X}_3^3\right)\left(\mathrm{Y}_3^{0,1,2}+\mathrm{Y}_3^3\right)}{\mathrm{K_{myx}}+\mathrm{X}_3^{0,1,2}+\mathrm{X}_3^3} \\ v_{10} &= \frac{\mathrm{k_d\,K_{mxy}\,C}\left(\mathrm{X}_3^{0,1,2}+\mathrm{X}_3^3\right)\left(\mathrm{Y}_3^{0,1,2}+\mathrm{Y}_3^3\right)}{\mathrm{K_{mxy}}+\mathrm{Y}_3^{0,1,2}+\mathrm{Y}_3^3} \end{aligned}$$

$$\begin{aligned}
v_{alt,1} &= -\,\mathrm{k_{12x}}\,\mathrm{C}\,\mathrm{X}_1^{0,1,2}\,\mathrm{X}_2^3 - \mathrm{k_{12x}}\,\mathrm{C}\,\mathrm{X}_1^3\,\left(\mathrm{X}_2^{0,1,2} - \mathrm{X_{2T}} + \mathrm{X}_2^3\right)\\
v_{alt,2} &= -\,\frac{\mathrm{C}\,\mathrm{k_x}\,\mathrm{S_1(t)}\,\mathrm{K_{mx}}\,\mathrm{X}_1^3}{\mathrm{S_1(t)} + \mathrm{K_{mx}}}\\
v_{alt,3} &= -\,\mathrm{k_{23x}}\,\mathrm{C}\,\mathrm{X}_2^{0,1,2}\,\mathrm{X}_3^3 - \mathrm{k_{23x}}\,\mathrm{C}\,\mathrm{X}_2^3\,\left(\mathrm{X}_3^{0,1,2} - \mathrm{X_{3T}} + \mathrm{X}_3^3\right)\\
v_{alt,4} &= -\,\frac{\mathrm{S_2(t)}\,\mathrm{k_y}\,\mathrm{K_{my}}\,\mathrm{C}\,\mathrm{Y}_1^3}{\mathrm{S_2(t)} + \mathrm{K_{my}}}\\
v_{alt,5} &= -\,\mathrm{k_{12y}}\,\mathrm{C}\,\mathrm{Y}_1^{0,1,2}\,\mathrm{Y}_2^3 - \mathrm{k_{12y}}\,\mathrm{C}\,\mathrm{Y}_1^3\,\left(\mathrm{Y}_2^{0,1,2} - \mathrm{Y_{2T}} + \mathrm{Y}_2^3\right)\\
v_{alt,6} &= -\,\mathrm{k_{23y}}\,\mathrm{C}\,\mathrm{Y}_2^{0,1,2}\,\mathrm{Y}_3^3 - \mathrm{k_{23y}}\,\mathrm{C}\,\mathrm{Y}_2^3\,\left(\mathrm{Y}_3^{0,1,2} - \mathrm{Y_{3T}} + \mathrm{Y}_3^3\right)
\end{aligned}$$

Layer Dynamics:

$$L(F^3|F^0, F^1, F^2) = \begin{bmatrix} X_1^3 & X_2^3 & X_3^3 & Y_1^3 & Y_2^3 & Y_3^3 \end{bmatrix}^T.$$

Layer $L(F^3|F^0, F^1, F^4)$

Required input trajectories:

$$\begin{bmatrix} X_1^{0,1,4} \\ X_2^{0,1,4} \\ X_3^{0,1,4} \\ Y_1^{0,1,4} \\ Y_2^{0,1,4} \\ Y_3^{0,1,4} \end{bmatrix} = \begin{bmatrix} 1 & 0 & 0 & 0 & 0 & 0 \\ 0 & 1 & 0 & 0 & 0 & 0 \\ 0 & 0 & 1 & 0 & 0 & 0 \\ 0 & 0 & 0 & 1 & 0 & 0 \\ 0 & 0 & 0 & 0 & 1 & 0 \\ 0 & 0 & 0 & 0 & 0 & 1 \end{bmatrix} L(F^0, F^1, F^4).$$

Ordinary differential equations:

$$\frac{d}{dt}\begin{bmatrix} X_1^3 \\ X_2^3 \\ X_3^3 \\ Y_1^3 \\ Y_2^3 \\ Y_3^3 \end{bmatrix} = \begin{bmatrix} 0 & 0 \\ 0 & 0 \\ -1 & 0 \\ 0 & 0 \\ 0 & 0 \\ 0 & -1 \end{bmatrix} \begin{bmatrix} v_9 \\ v_{10} \end{bmatrix} + \begin{bmatrix} -1 & 1 & 0 & -1 & 0 \\ 1 & 0 & -1 & 0 & 1 \\ 0 & 0 & 1 & 0 & 0 \\ 0 & 0 & 0 & 0 & -1 \\ 0 & 0 & 0 & 1 & 0 \\ 0 & 0 & 0 & 0 & 0 \end{bmatrix} \begin{bmatrix} v_{alt,1} \\ v_{alt,2} \\ v_{alt,3} \\ v_{alt,7} \\ v_{alt,8} \end{bmatrix},$$

with

$$\begin{aligned}
v_9 &= \frac{\mathrm{k_d}\,\mathrm{K_{myx}}\,\mathrm{C}\,\left(\mathrm{X}_3^{0,1,4} + \mathrm{X}_3^3\right)\,\left(\mathrm{Y}_3^{0,1,4} + \mathrm{Y}_3^3\right)}{\mathrm{K_{myx}} + \mathrm{X}_3^{0,1,4} + \mathrm{X}_3^3}\\
v_{10} &= \frac{\mathrm{k_d}\,\mathrm{K_{mxy}}\,\mathrm{C}\,\left(\mathrm{X}_3^{0,1,4} + \mathrm{X}_3^3\right)\,\left(\mathrm{Y}_3^{0,1,4} + \mathrm{Y}_3^3\right)}{\mathrm{K_{mxy}} + \mathrm{Y}_3^{0,1,4} + \mathrm{Y}_3^3}\\
v_{alt,1} &= -\,\mathrm{k_{12x}}\,\mathrm{C}\,\mathrm{X}_1^{0,1,4}\,\mathrm{X}_2^3 - \mathrm{k_{12x}}\,\mathrm{C}\,\mathrm{X}_1^3\,\left(\mathrm{X}_2^{0,1,4} - \mathrm{X_{2T}} + \mathrm{X}_2^3\right)\\
v_{alt,2} &= -\,\frac{\mathrm{C}\,\mathrm{k_x}\,\mathrm{S_1(t)}\,\mathrm{K_{mx}}\,\mathrm{X}_1^3}{\mathrm{S_1(t)} + \mathrm{K_{mx}}}\\
v_{alt,3} &= -\,\mathrm{k_{23x}}\,\mathrm{C}\,\mathrm{X}_2^{0,1,4}\,\mathrm{X}_3^3 - \mathrm{k_{23x}}\,\mathrm{C}\,\mathrm{X}_2^3\,\left(\mathrm{X}_3^{0,1,4} - \mathrm{X_{3T}} + \mathrm{X}_3^3\right)
\end{aligned}$$

$$v_{alt,7} = -\,\mathrm{k_a}\,\mathrm{C}\,\mathrm{X}_1^{0,1,4}\,\mathrm{Y}_2^3 - \mathrm{k_a}\,\mathrm{C}\,\mathrm{X}_1^3\,\left(\mathrm{Y}_2^{0,1,4} - \mathrm{Y_{2T}} + \mathrm{Y}_2^3\right)$$
$$v_{alt,8} = -\,\mathrm{k_a}\,\mathrm{C}\,\mathrm{X}_2^3\,\mathrm{Y}_1^{0,1,4} - \mathrm{k_a}\,\mathrm{C}\,\mathrm{Y}_1^3\,\left(\mathrm{X}_2^{0,1,4} - \mathrm{X_{2T}} + \mathrm{X}_2^3\right)$$

Layer Dynamics:

$$L(F^3|F^0, F^1, F^4) = \begin{bmatrix} X_1^3 & X_2^3 & X_3^3 & Y_1^3 & Y_2^3 & Y_3^3 \end{bmatrix}^T.$$

Layer $L(F^3|F^0, F^2, F^4)$

Required input trajectories:

$$\begin{bmatrix} X_1^{0,2,4} \\ X_2^{0,2,4} \\ X_3^{0,2,4} \\ Y_1^{0,2,4} \\ Y_2^{0,2,4} \\ Y_3^{0,2,4} \end{bmatrix} = \begin{bmatrix} 1 & 0 & 0 & 0 & 0 & 0 \\ 0 & 1 & 0 & 0 & 0 & 0 \\ 0 & 0 & 1 & 0 & 0 & 0 \\ 0 & 0 & 0 & 1 & 0 & 0 \\ 0 & 0 & 0 & 0 & 1 & 0 \\ 0 & 0 & 0 & 0 & 0 & 1 \end{bmatrix} L(F^0, F^2, F^4).$$

Ordinary differential equations:

$$\frac{d}{dt}\begin{bmatrix} X_1^3 \\ X_2^3 \\ X_3^3 \\ Y_1^3 \\ Y_2^3 \\ Y_3^3 \end{bmatrix} = \begin{bmatrix} 0 & 0 \\ 0 & 0 \\ -1 & 0 \\ 0 & 0 \\ 0 & 0 \\ 0 & -1 \end{bmatrix} \begin{bmatrix} v_9 \\ v_{10} \end{bmatrix} + \begin{bmatrix} 0 & 0 & 0 & -1 & 0 \\ 0 & 0 & 0 & 0 & 1 \\ 0 & 0 & 0 & 0 & 0 \\ 1 & -1 & 0 & 0 & -1 \\ 0 & 1 & -1 & 1 & 0 \\ 0 & 0 & 1 & 0 & 0 \end{bmatrix} \begin{bmatrix} v_{alt,4} \\ v_{alt,5} \\ v_{alt,6} \\ v_{alt,7} \\ v_{alt,8} \end{bmatrix},$$

with

$$v_9 = \frac{\mathrm{k_d}\,\mathrm{K_{myx}}\,\mathrm{C}\,\left(\mathrm{X}_3^{0,2,4} + \mathrm{X}_3^3\right)\,\left(\mathrm{Y}_3^{0,2,4} + \mathrm{Y}_3^3\right)}{\mathrm{K_{myx}} + \mathrm{X}_3^{0,2,4} + \mathrm{X}_3^3}$$
$$v_{10} = \frac{\mathrm{k_d}\,\mathrm{K_{mxy}}\,\mathrm{C}\,\left(\mathrm{X}_3^{0,2,4} + \mathrm{X}_3^3\right)\,\left(\mathrm{Y}_3^{0,2,4} + \mathrm{Y}_3^3\right)}{\mathrm{K_{mxy}} + \mathrm{Y}_3^{0,2,4} + \mathrm{Y}_3^3}$$
$$v_{alt,4} = -\,\frac{\mathrm{S_2(t)}\,\mathrm{k_y}\,\mathrm{K_{my}}\,\mathrm{C}\,\mathrm{Y}_1^3}{\mathrm{S_2(t)} + \mathrm{K_{my}}}$$
$$v_{alt,5} = -\,\mathrm{k_{12y}}\,\mathrm{C}\,\mathrm{Y}_1^{0,2,4}\,\mathrm{Y}_2^3 - \mathrm{k_{12y}}\,\mathrm{C}\,\mathrm{Y}_1^3\,\left(\mathrm{Y}_2^{0,2,4} - \mathrm{Y_{2T}} + \mathrm{Y}_2^3\right)$$
$$v_{alt,6} = -\,\mathrm{k_{23y}}\,\mathrm{C}\,\mathrm{Y}_2^{0,2,4}\,\mathrm{Y}_3^3 - \mathrm{k_{23y}}\,\mathrm{C}\,\mathrm{Y}_2^3\,\left(\mathrm{Y}_3^{0,2,4} - \mathrm{Y_{3T}} + \mathrm{Y}_3^3\right)$$
$$v_{alt,7} = -\,\mathrm{k_a}\,\mathrm{C}\,\mathrm{X}_1^{0,2,4}\,\mathrm{Y}_2^3 - \mathrm{k_a}\,\mathrm{C}\,\mathrm{X}_1^3\,\left(\mathrm{Y}_2^{0,2,4} - \mathrm{Y_{2T}} + \mathrm{Y}_2^3\right)$$
$$v_{alt,8} = -\,\mathrm{k_a}\,\mathrm{C}\,\mathrm{X}_2^3\,\mathrm{Y}_1^{0,2,4} - \mathrm{k_a}\,\mathrm{C}\,\mathrm{Y}_1^3\,\left(\mathrm{X}_2^{0,2,4} - \mathrm{X_{2T}} + \mathrm{X}_2^3\right)$$

Layer Dynamics:

$$L(F^3|F^0, F^2, F^4) = \begin{bmatrix} X_1^3 & X_2^3 & X_3^3 & Y_1^3 & Y_2^3 & Y_3^3 \end{bmatrix}^T.$$

Layer $L(F^3|F^0, F^1, F^2, F^4)$

Required input trajectories:

$$\begin{bmatrix} X_1^{0,1,2,4} \\ X_2^{0,1,2,4} \\ X_3^{0,1,2,4} \\ Y_1^{0,1,2,4} \\ Y_2^{0,1,2,4} \\ Y_3^{0,1,2,4} \end{bmatrix} = \begin{bmatrix} 1 & 0 & 0 & 0 & 0 & 0 \\ 0 & 1 & 0 & 0 & 0 & 0 \\ 0 & 0 & 1 & 0 & 0 & 0 \\ 0 & 0 & 0 & 1 & 0 & 0 \\ 0 & 0 & 0 & 0 & 1 & 0 \\ 0 & 0 & 0 & 0 & 0 & 1 \end{bmatrix} L(F^0, F^1, F^2, F^4).$$

Ordinary differential equations:

$$\frac{d}{dt} \begin{bmatrix} X_1^3 \\ X_2^3 \\ X_3^3 \\ Y_1^3 \\ Y_2^3 \\ Y_3^3 \end{bmatrix} = \begin{bmatrix} 0 & 0 \\ 0 & 0 \\ -1 & 0 \\ 0 & 0 \\ 0 & 0 \\ 0 & -1 \end{bmatrix} \begin{bmatrix} v_9 \\ v_{10} \end{bmatrix} + \begin{bmatrix} -1 & 1 & 0 & -1 & 0 & 0 & 0 & 0 \\ 1 & 0 & -1 & 0 & 1 & 0 & 0 & 0 \\ 0 & 0 & 1 & 0 & 0 & 0 & 0 & 0 \\ 0 & 0 & 0 & 0 & -1 & 1 & -1 & 0 \\ 0 & 0 & 0 & 1 & 0 & 0 & 1 & -1 \\ 0 & 0 & 0 & 0 & 0 & 0 & 0 & 1 \end{bmatrix} \begin{bmatrix} v_{alt,1} \\ v_{alt,2} \\ v_{alt,3} \\ v_{alt,7} \\ v_{alt,8} \\ v_{alt,4} \\ v_{alt,5} \\ v_{alt,6} \end{bmatrix},$$

with

$$\begin{aligned}
v_9 =& \frac{\mathrm{k_d\, K_{myx}\, C} \left(\mathrm{X_3^{0,1,2,4}} + \mathrm{X_3^3}\right) \left(\mathrm{Y_3^{0,1,2,4}} + \mathrm{Y_3^3}\right)}{\mathrm{K_{myx}} + \mathrm{X_3^{0,1,2,4}} + \mathrm{X_3^3}} \\
v_{10} =& \frac{\mathrm{k_d\, K_{mxy}\, C} \left(\mathrm{X_3^{0,1,2,4}} + \mathrm{X_3^3}\right) \left(\mathrm{Y_3^{0,1,2,4}} + \mathrm{Y_3^3}\right)}{\mathrm{K_{mxy}} + \mathrm{Y_3^{0,1,2,4}} + \mathrm{Y_3^3}} \\
v_{alt,1} =& -\mathrm{k_{12x}\, C\, X_1^{0,1,2,4}\, X_2^3} - \mathrm{k_{12x}\, C\, X_1^3} \left(\mathrm{X_2^{0,1,2,4}} - \mathrm{X_{2T}} + \mathrm{X_2^3}\right) \\
v_{alt,2} =& -\frac{\mathrm{C\, k_x\, S_1(t)\, K_{mx}\, X_1^3}}{\mathrm{S_1(t)} + \mathrm{K_{mx}}} \\
v_{alt,3} =& -\mathrm{k_{23x}\, C\, X_2^{0,1,2,4}\, X_3^3} - \mathrm{k_{23x}\, C\, X_2^3} \left(\mathrm{X_3^{0,1,2,4}} - \mathrm{X_{3T}} + \mathrm{X_3^3}\right) \\
v_{alt,7} =& -\mathrm{k_a\, C\, X_1^{0,1,2,4}\, Y_2^3} - \mathrm{k_a\, C\, X_1^3} \left(\mathrm{Y_2^{0,1,2,4}} - \mathrm{Y_{2T}} + \mathrm{Y_2^3}\right) \\
v_{alt,8} =& -\mathrm{k_a\, C\, X_2^3\, Y_1^{0,1,2,4}} - \mathrm{k_a\, C\, Y_1^3} \left(\mathrm{X_2^{0,1,2,4}} - \mathrm{X_{2T}} + \mathrm{X_2^3}\right) \\
v_{alt,4} =& -\frac{\mathrm{S_2(t)\, k_y\, K_{my}\, C\, Y_1^3}}{\mathrm{S_2(t)} + \mathrm{K_{my}}} \\
v_{alt,5} =& -\mathrm{k_{12y}\, C\, Y_1^{0,1,2,4}\, Y_2^3} - \mathrm{k_{12y}\, C\, Y_1^3} \left(\mathrm{Y_2^{0,1,2,4}} - \mathrm{Y_{2T}} + \mathrm{Y_2^3}\right) \\
v_{alt,6} =& -\mathrm{k_{23y}\, C\, Y_2^{0,1,2,4}\, Y_3^3} - \mathrm{k_{23y}\, C\, Y_2^3} \left(\mathrm{Y_3^{0,1,2,4}} - \mathrm{Y_{3T}} + \mathrm{Y_3^3}\right)
\end{aligned}$$

Layer Dynamics:

$$L(F^3|F^0, F^1, F^2, F^4) = \begin{bmatrix} X_1^3 & X_2^3 & X_3^3 & Y_1^3 & Y_2^3 & Y_3^3 \end{bmatrix}^T.$$

Layer $L(F^4|F^0)$

Required input trajectories:

$$\begin{bmatrix} X_1^0 \\ X_2^0 \\ Y_1^0 \\ Y_2^0 \end{bmatrix} = \begin{bmatrix} 1 & 0 & 0 & 0 & 0 & 0 \\ 0 & 1 & 0 & 0 & 0 & 0 \\ 0 & 0 & 0 & 1 & 0 & 0 \\ 0 & 0 & 0 & 0 & 1 & 0 \end{bmatrix} L(F^0).$$

Ordinary differential equations:

$$\frac{d}{dt}\begin{bmatrix} X_1^4 \\ X_2^4 \\ Y_1^4 \\ Y_2^4 \end{bmatrix} = \begin{bmatrix} -1 & 0 \\ 0 & 1 \\ 0 & -1 \\ 1 & 0 \end{bmatrix} \begin{bmatrix} v_7 \\ v_8 \end{bmatrix},$$

with

$$v_7 = -\,\mathrm{k_a}\,\mathrm{C}\,\left(\mathrm{X_1^0} + \mathrm{X_1^4}\right)\,\left(\mathrm{Y_2^0} - \mathrm{Y_{2T}} + \mathrm{Y_2^4}\right)$$
$$v_8 = -\,\mathrm{k_a}\,\mathrm{C}\,\left(\mathrm{Y_1^0} + \mathrm{Y_1^4}\right)\,\left(\mathrm{X_2^0} - \mathrm{X_{2T}} + \mathrm{X_2^4}\right)$$

Layer Dynamics:

$$L(F^4|F^0) = \begin{bmatrix} X_1^4 & X_2^4 & 0 & Y_1^4 & Y_2^4 & 0 \end{bmatrix}^T.$$

Layer $L(F^4|F^0, F^1)$

Required input trajectories:

$$\begin{bmatrix} X_1^{0,1} \\ X_2^{0,1} \\ X_3^{0,1} \\ Y_1^{0,1} \\ Y_2^{0,1} \end{bmatrix} = \begin{bmatrix} 1 & 0 & 0 & 0 & 0 & 0 \\ 0 & 1 & 0 & 0 & 0 & 0 \\ 0 & 0 & 1 & 0 & 0 & 0 \\ 0 & 0 & 0 & 1 & 0 & 0 \\ 0 & 0 & 0 & 0 & 1 & 0 \end{bmatrix} L(F^0, F^1).$$

Ordinary differential equations:

$$\frac{d}{dt}\begin{bmatrix} X_1^4 \\ X_2^4 \\ X_3^4 \\ Y_1^4 \\ Y_2^4 \end{bmatrix} = \begin{bmatrix} -1 & 0 \\ 0 & 1 \\ 0 & 0 \\ 0 & -1 \\ 1 & 0 \end{bmatrix} \begin{bmatrix} v_7 \\ v_8 \end{bmatrix} + \begin{bmatrix} -1 & 1 & 0 \\ 1 & 0 & -1 \\ 0 & 0 & 1 \\ 0 & 0 & 0 \\ 0 & 0 & 0 \end{bmatrix} \begin{bmatrix} v_{alt,1} \\ v_{alt,2} \\ v_{alt,3} \end{bmatrix},$$

with

$$v_7 = -\,\mathrm{k_a}\,\mathrm{C}\,\left(\mathrm{X_1^{0,1}} + \mathrm{X_1^4}\right)\,\left(\mathrm{Y_2^{0,1}} - \mathrm{Y_{2T}} + \mathrm{Y_2^4}\right)$$
$$v_8 = -\,\mathrm{k_a}\,\mathrm{C}\,\left(\mathrm{Y_1^{0,1}} + \mathrm{Y_1^4}\right)\,\left(\mathrm{X_2^{0,1}} - \mathrm{X_{2T}} + \mathrm{X_2^4}\right)$$
$$v_{alt,1} = -\,\mathrm{k_{12x}}\,\mathrm{C}\,\mathrm{X_1^{0,1}}\,\mathrm{X_2^4} - \mathrm{k_{12x}}\,\mathrm{C}\,\mathrm{X_1^4}\,\left(\mathrm{X_2^{0,1}} - \mathrm{X_{2T}} + \mathrm{X_2^4}\right)$$
$$v_{alt,2} = -\,\frac{\mathrm{C}\,\mathrm{k_x}\,\mathrm{S_1(t)}\,\mathrm{K_{mx}}\,\mathrm{X_1^4}}{\mathrm{S_1(t)} + \mathrm{K_{mx}}}$$

$$v_{alt,3} = -\,\mathrm{k_{23x}}\,\mathrm{C}\,\mathrm{X_2^{0,1}}\,\mathrm{X_3^4} - \mathrm{k_{23x}}\,\mathrm{C}\,\mathrm{X_2^4}\left(\mathrm{X_3^{0,1}} - \mathrm{X_{3T}} + \mathrm{X_3^4}\right)$$

Layer Dynamics:

$$L(F^4|F^0,F^1) = \begin{bmatrix} X_1^4 & X_2^4 & X_3^4 & Y_1^4 & Y_2^4 & 0 \end{bmatrix}^T.$$

Layer $L(F^4|F^0,F^2)$

Required input trajectories:

$$\begin{bmatrix} X_1^{0,2} \\ X_2^{0,2} \\ Y_1^{0,2} \\ Y_2^{0,2} \\ Y_3^{0,2} \end{bmatrix} = \begin{bmatrix} 1 & 0 & 0 & 0 & 0 & 0 \\ 0 & 1 & 0 & 0 & 0 & 0 \\ 0 & 0 & 0 & 1 & 0 & 0 \\ 0 & 0 & 0 & 0 & 1 & 0 \\ 0 & 0 & 0 & 0 & 0 & 1 \end{bmatrix} L(F^0,F^2).$$

Ordinary differential equations:

$$\frac{d}{dt}\begin{bmatrix} X_1^4 \\ X_2^4 \\ Y_1^4 \\ Y_2^4 \\ Y_3^4 \end{bmatrix} = \begin{bmatrix} -1 & 0 \\ 0 & 1 \\ 0 & -1 \\ 1 & 0 \\ 0 & 0 \end{bmatrix} \begin{bmatrix} v_7 \\ v_8 \end{bmatrix} + \begin{bmatrix} 0 & 0 & 0 \\ 0 & 0 & 0 \\ 1 & -1 & 0 \\ 0 & 1 & -1 \\ 0 & 0 & 1 \end{bmatrix} \begin{bmatrix} v_{alt,4} \\ v_{alt,5} \\ v_{alt,6} \end{bmatrix},$$

with

$$\begin{aligned}
v_7 &= -\,\mathrm{k_a}\,\mathrm{C}\left(\mathrm{X_1^{0,2}} + \mathrm{X_1^4}\right)\left(\mathrm{Y_2^{0,2}} - \mathrm{Y_{2T}} + \mathrm{Y_2^4}\right) \\
v_8 &= -\,\mathrm{k_a}\,\mathrm{C}\left(\mathrm{Y_1^{0,2}} + \mathrm{Y_1^4}\right)\left(\mathrm{X_2^{0,2}} - \mathrm{X_{2T}} + \mathrm{X_2^4}\right) \\
v_{alt,4} &= -\,\frac{\mathrm{S_2(t)}\,\mathrm{k_y}\,\mathrm{K_{my}}\,\mathrm{C}\,\mathrm{Y_1^4}}{\mathrm{S_2(t)} + \mathrm{K_{my}}} \\
v_{alt,5} &= -\,\mathrm{k_{12y}}\,\mathrm{C}\,\mathrm{Y_1^{0,2}}\,\mathrm{Y_2^4} - \mathrm{k_{12y}}\,\mathrm{C}\,\mathrm{Y_1^4}\left(\mathrm{Y_2^{0,2}} - \mathrm{Y_{2T}} + \mathrm{Y_2^4}\right) \\
v_{alt,6} &= -\,\mathrm{k_{23y}}\,\mathrm{C}\,\mathrm{Y_2^{0,2}}\,\mathrm{Y_3^4} - \mathrm{k_{23y}}\,\mathrm{C}\,\mathrm{Y_2^4}\left(\mathrm{Y_3^{0,2}} - \mathrm{Y_{3T}} + \mathrm{Y_3^4}\right)
\end{aligned}$$

Layer Dynamics:

$$L(F^4|F^0,F^2) = \begin{bmatrix} X_1^4 & X_2^4 & 0 & Y_1^4 & Y_2^4 & Y_3^4 \end{bmatrix}^T.$$

Layer $L(F^4|F^0,F^3)$

Required input trajectories:

$$\begin{bmatrix} X_1^{0,3} \\ X_2^{0,3} \\ Y_1^{0,3} \\ Y_2^{0,3} \end{bmatrix} = \begin{bmatrix} 1 & 0 & 0 & 0 & 0 & 0 \\ 0 & 1 & 0 & 0 & 0 & 0 \\ 0 & 0 & 0 & 1 & 0 & 0 \\ 0 & 0 & 0 & 0 & 1 & 0 \end{bmatrix} L(F^0,F^3).$$

Ordinary differential equations:

$$\frac{d}{dt}\begin{bmatrix} X_1^4 \\ X_2^4 \\ Y_1^4 \\ Y_2^4 \end{bmatrix} = \begin{bmatrix} -1 & 0 \\ 0 & 1 \\ 0 & -1 \\ 1 & 0 \end{bmatrix} \begin{bmatrix} v_7 \\ v_8 \end{bmatrix},$$

with

$$\begin{aligned} v_7 &= -\,\mathrm{k_a}\,\mathrm{C}\left(\mathrm{X}_1^{0,3} + \mathrm{X}_1^4\right)\left(\mathrm{Y}_2^{0,3} - \mathrm{Y}_{2\mathrm{T}} + \mathrm{Y}_2^4\right) \\ v_8 &= -\,\mathrm{k_a}\,\mathrm{C}\left(\mathrm{Y}_1^{0,3} + \mathrm{Y}_1^4\right)\left(\mathrm{X}_2^{0,3} - \mathrm{X}_{2\mathrm{T}} + \mathrm{X}_2^4\right) \end{aligned}$$

Layer Dynamics:

$$L(F^4|F^0, F^3) = \begin{bmatrix} X_1^4 & X_2^4 & 0 & Y_1^4 & Y_2^4 & 0 \end{bmatrix}^T.$$

Layer $L(F^4|F^0, F^1, F^2)$

Required input trajectories:

$$\begin{bmatrix} X_1^{0,1,2} \\ X_2^{0,1,2} \\ X_3^{0,1,2} \\ Y_1^{0,1,2} \\ Y_2^{0,1,2} \\ Y_3^{0,1,2} \end{bmatrix} = \begin{bmatrix} 1 & 0 & 0 & 0 & 0 & 0 \\ 0 & 1 & 0 & 0 & 0 & 0 \\ 0 & 0 & 1 & 0 & 0 & 0 \\ 0 & 0 & 0 & 1 & 0 & 0 \\ 0 & 0 & 0 & 0 & 1 & 0 \\ 0 & 0 & 0 & 0 & 0 & 1 \end{bmatrix} L(F^0, F^1, F^2).$$

Ordinary differential equations:

$$\frac{d}{dt}\begin{bmatrix} X_1^4 \\ X_2^4 \\ X_3^4 \\ Y_1^4 \\ Y_2^4 \\ Y_3^4 \end{bmatrix} = \begin{bmatrix} -1 & 0 \\ 0 & 1 \\ 0 & 0 \\ 0 & -1 \\ 1 & 0 \\ 0 & 0 \end{bmatrix} \begin{bmatrix} v_7 \\ v_8 \end{bmatrix} + \begin{bmatrix} -1 & 1 & 0 & 0 & 0 & 0 \\ 1 & 0 & -1 & 0 & 0 & 0 \\ 0 & 0 & 1 & 0 & 0 & 0 \\ 0 & 0 & 0 & 1 & -1 & 0 \\ 0 & 0 & 0 & 0 & 1 & -1 \\ 0 & 0 & 0 & 0 & 0 & 1 \end{bmatrix} \begin{bmatrix} v_{alt,1} \\ v_{alt,2} \\ v_{alt,3} \\ v_{alt,4} \\ v_{alt,5} \\ v_{alt,6} \end{bmatrix},$$

with

$$\begin{aligned} v_7 &= -\,\mathrm{k_a}\,\mathrm{C}\left(\mathrm{X}_1^{0,1,2} + \mathrm{X}_1^4\right)\left(\mathrm{Y}_2^{0,1,2} - \mathrm{Y}_{2\mathrm{T}} + \mathrm{Y}_2^4\right) \\ v_8 &= -\,\mathrm{k_a}\,\mathrm{C}\left(\mathrm{Y}_1^{0,1,2} + \mathrm{Y}_1^4\right)\left(\mathrm{X}_2^{0,1,2} - \mathrm{X}_{2\mathrm{T}} + \mathrm{X}_2^4\right) \\ v_{alt,1} &= -\,\mathrm{k_{12x}}\,\mathrm{C}\,\mathrm{X}_1^{0,1,2}\,\mathrm{X}_2^4 - \mathrm{k_{12x}}\,\mathrm{C}\,\mathrm{X}_1^4\left(\mathrm{X}_2^{0,1,2} - \mathrm{X}_{2\mathrm{T}} + \mathrm{X}_2^4\right) \\ v_{alt,2} &= -\,\frac{\mathrm{C}\,\mathrm{k_x}\,\mathrm{S}_1(\mathrm{t})\,\mathrm{K_{mx}}\,\mathrm{X}_1^4}{\mathrm{S}_1(\mathrm{t}) + \mathrm{K_{mx}}} \\ v_{alt,3} &= -\,\mathrm{k_{23x}}\,\mathrm{C}\,\mathrm{X}_2^{0,1,2}\,\mathrm{X}_3^4 - \mathrm{k_{23x}}\,\mathrm{C}\,\mathrm{X}_2^4\left(\mathrm{X}_3^{0,1,2} - \mathrm{X}_{3\mathrm{T}} + \mathrm{X}_3^4\right) \\ v_{alt,4} &= -\,\frac{\mathrm{S}_2(\mathrm{t})\,\mathrm{k_y}\,\mathrm{K_{my}}\,\mathrm{C}\,\mathrm{Y}_1^4}{\mathrm{S}_2(\mathrm{t}) + \mathrm{K_{my}}} \end{aligned}$$

$$v_{alt,5} = -\,\mathrm{k_{12y}}\,\mathrm{C}\,\mathrm{Y}_1^{0,1,2}\,\mathrm{Y}_2^4 - \mathrm{k_{12y}}\,\mathrm{C}\,\mathrm{Y}_1^4\,\left(\mathrm{Y}_2^{0,1,2} - \mathrm{Y_{2T}} + \mathrm{Y}_2^4\right)$$

$$v_{alt,6} = -\,\mathrm{k_{23y}}\,\mathrm{C}\,\mathrm{Y}_2^{0,1,2}\,\mathrm{Y}_3^4 - \mathrm{k_{23y}}\,\mathrm{C}\,\mathrm{Y}_2^4\,\left(\mathrm{Y}_3^{0,1,2} - \mathrm{Y_{3T}} + \mathrm{Y}_3^4\right)$$

Layer Dynamics:

$$L(F^4|F^0,F^1,F^2) = \begin{bmatrix} X_1^4 & X_2^4 & X_3^4 & Y_1^4 & Y_2^4 & Y_3^4 \end{bmatrix}^T.$$

Layer $L(F^4|F^0,F^1,F^3)$

Required input trajectories:

$$\begin{bmatrix} X_1^{0,1,3} \\ X_2^{0,1,3} \\ X_3^{0,1,3} \\ Y_1^{0,1,3} \\ Y_2^{0,1,3} \\ Y_3^{0,1,3} \end{bmatrix} = \begin{bmatrix} 1 & 0 & 0 & 0 & 0 & 0 \\ 0 & 1 & 0 & 0 & 0 & 0 \\ 0 & 0 & 1 & 0 & 0 & 0 \\ 0 & 0 & 0 & 1 & 0 & 0 \\ 0 & 0 & 0 & 0 & 1 & 0 \\ 0 & 0 & 0 & 0 & 0 & 1 \end{bmatrix} L(F^0,F^1,F^3).$$

Ordinary differential equations:

$$\frac{d}{dt}\begin{bmatrix} X_1^4 \\ X_2^4 \\ X_3^4 \\ Y_1^4 \\ Y_2^4 \\ Y_3^4 \end{bmatrix} = \begin{bmatrix} -1 & 0 \\ 0 & 1 \\ 0 & 0 \\ 0 & -1 \\ 1 & 0 \\ 0 & 0 \end{bmatrix} \begin{bmatrix} v_7 \\ v_8 \end{bmatrix} + \begin{bmatrix} -1 & 1 & 0 & 0 & 0 \\ 1 & 0 & -1 & 0 & 0 \\ 0 & 0 & 1 & -1 & 0 \\ 0 & 0 & 0 & 0 & 0 \\ 0 & 0 & 0 & 0 & 0 \\ 0 & 0 & 0 & 0 & -1 \end{bmatrix} \begin{bmatrix} v_{alt,1} \\ v_{alt,2} \\ v_{alt,3} \\ v_{alt,9} \\ v_{alt,10} \end{bmatrix},$$

with

$$v_7 = -\,\mathrm{k_a}\,\mathrm{C}\,\left(\mathrm{X}_1^{0,1,3} + \mathrm{X}_1^4\right)\,\left(\mathrm{Y}_2^{0,1,3} - \mathrm{Y_{2T}} + \mathrm{Y}_2^4\right)$$

$$v_8 = -\,\mathrm{k_a}\,\mathrm{C}\,\left(\mathrm{Y}_1^{0,1,3} + \mathrm{Y}_1^4\right)\,\left(\mathrm{X}_2^{0,1,3} - \mathrm{X_{2T}} + \mathrm{X}_2^4\right)$$

$$v_{alt,1} = -\,\mathrm{k_{12x}}\,\mathrm{C}\,\mathrm{X}_1^{0,1,3}\,\mathrm{X}_2^4 - \mathrm{k_{12x}}\,\mathrm{C}\,\mathrm{X}_1^4\,\left(\mathrm{X}_2^{0,1,3} - \mathrm{X_{2T}} + \mathrm{X}_2^4\right)$$

$$v_{alt,2} = -\,\frac{\mathrm{C}\,\mathrm{k_x}\,\mathrm{S_1(t)}\,\mathrm{K_{mx}}\,\mathrm{X}_1^4}{\mathrm{S_1(t)} + \mathrm{K_{mx}}}$$

$$v_{alt,3} = -\,\mathrm{k_{23x}}\,\mathrm{C}\,\mathrm{X}_2^{0,1,3}\,\mathrm{X}_3^4 - \mathrm{k_{23x}}\,\mathrm{C}\,\mathrm{X}_2^4\,\left(\mathrm{X}_3^{0,1,3} - \mathrm{X_{3T}} + \mathrm{X}_3^4\right)$$

$$v_{alt,9} = \frac{\mathrm{k_d}\,\mathrm{C}\,\left(\mathrm{X}_3^{0,1,3} + \mathrm{X}_3^4\right)\,\left(\mathrm{Y}_3^{0,1,3} + \mathrm{Y}_3^4\right)}{\frac{\mathrm{X}_3^{0,1,3}+\mathrm{X}_3^4}{\mathrm{K_{myx}}} + 1} - \frac{\mathrm{k_d}\,\mathrm{C}\,\mathrm{X}_3^{0,1,3}\,\mathrm{Y}_3^{0,1,3}}{\frac{\mathrm{X}_3^{0,1,3}}{\mathrm{K_{myx}}} + 1}$$

$$v_{alt,10} = \frac{\mathrm{k_d}\,\mathrm{C}\,\left(\mathrm{X}_3^{0,1,3} + \mathrm{X}_3^4\right)\,\left(\mathrm{Y}_3^{0,1,3} + \mathrm{Y}_3^4\right)}{\frac{\mathrm{Y}_3^{0,1,3}+\mathrm{Y}_3^4}{\mathrm{K_{mxy}}} + 1} - \frac{\mathrm{k_d}\,\mathrm{C}\,\mathrm{X}_3^{0,1,3}\,\mathrm{Y}_3^{0,1,3}}{\frac{\mathrm{Y}_3^{0,1,3}}{\mathrm{K_{mxy}}} + 1}$$

Layer Dynamics:

$$L(F^4|F^0,F^1,F^3) = \begin{bmatrix} X_1^4 & X_2^4 & X_3^4 & Y_1^4 & Y_2^4 & Y_3^4 \end{bmatrix}^T.$$

Layer $L(F^4|F^0, F^2, F^3)$

Required input trajectories:

$$\begin{bmatrix} X_1^{0,2,3} \\ X_2^{0,2,3} \\ X_3^{0,2,3} \\ Y_1^{0,2,3} \\ Y_2^{0,2,3} \\ Y_3^{0,2,3} \end{bmatrix} = \begin{bmatrix} 1 & 0 & 0 & 0 & 0 & 0 \\ 0 & 1 & 0 & 0 & 0 & 0 \\ 0 & 0 & 1 & 0 & 0 & 0 \\ 0 & 0 & 0 & 1 & 0 & 0 \\ 0 & 0 & 0 & 0 & 1 & 0 \\ 0 & 0 & 0 & 0 & 0 & 1 \end{bmatrix} L(F^0, F^2, F^3).$$

Ordinary differential equations:

$$\frac{d}{dt} \begin{bmatrix} X_1^4 \\ X_2^4 \\ X_3^4 \\ Y_1^4 \\ Y_2^4 \\ Y_3^4 \end{bmatrix} = \begin{bmatrix} -1 & 0 \\ 0 & 1 \\ 0 & 0 \\ 0 & -1 \\ 1 & 0 \\ 0 & 0 \end{bmatrix} \begin{bmatrix} v_7 \\ v_8 \end{bmatrix} + \begin{bmatrix} 0 & 0 & 0 & 0 & 0 \\ 0 & 0 & 0 & 0 & 0 \\ 0 & 0 & 0 & -1 & 0 \\ 1 & -1 & 0 & 0 & 0 \\ 0 & 1 & -1 & 0 & 0 \\ 0 & 0 & 1 & 0 & -1 \end{bmatrix} \begin{bmatrix} v_{alt,4} \\ v_{alt,5} \\ v_{alt,6} \\ v_{alt,9} \\ v_{alt,10} \end{bmatrix},$$

with

$$\begin{aligned}
v_7 &= -\,\mathrm{k_a}\,\mathrm{C}\left(\mathrm{X}_1^{0,2,3} + \mathrm{X}_1^4\right)\left(\mathrm{Y}_2^{0,2,3} - \mathrm{Y_{2T}} + \mathrm{Y}_2^4\right) \\
v_8 &= -\,\mathrm{k_a}\,\mathrm{C}\left(\mathrm{Y}_1^{0,2,3} + \mathrm{Y}_1^4\right)\left(\mathrm{X}_2^{0,2,3} - \mathrm{X_{2T}} + \mathrm{X}_2^4\right) \\
v_{alt,4} &= -\,\frac{\mathrm{S_2(t)}\,\mathrm{k_y}\,\mathrm{K_{my}}\,\mathrm{C}\,\mathrm{Y}_1^4}{\mathrm{S_2(t)} + \mathrm{K_{my}}} \\
v_{alt,5} &= -\,\mathrm{k_{12y}}\,\mathrm{C}\,\mathrm{Y}_1^{0,2,3}\,\mathrm{Y}_2^4 - \mathrm{k_{12y}}\,\mathrm{C}\,\mathrm{Y}_1^4\left(\mathrm{Y}_2^{0,2,3} - \mathrm{Y_{2T}} + \mathrm{Y}_2^4\right) \\
v_{alt,6} &= -\,\mathrm{k_{23y}}\,\mathrm{C}\,\mathrm{Y}_2^{0,2,3}\,\mathrm{Y}_3^4 - \mathrm{k_{23y}}\,\mathrm{C}\,\mathrm{Y}_2^4\left(\mathrm{Y}_3^{0,2,3} - \mathrm{Y_{3T}} + \mathrm{Y}_3^4\right) \\
v_{alt,9} &= \frac{\mathrm{k_d}\,\mathrm{C}\left(\mathrm{X}_3^{0,2,3} + \mathrm{X}_3^4\right)\left(\mathrm{Y}_3^{0,2,3} + \mathrm{Y}_3^4\right)}{\frac{\mathrm{X}_3^{0,2,3} + \mathrm{X}_3^4}{\mathrm{K_{myx}}} + 1} - \frac{\mathrm{k_d}\,\mathrm{C}\,\mathrm{X}_3^{0,2,3}\,\mathrm{Y}_3^{0,2,3}}{\frac{\mathrm{X}_3^{0,2,3}}{\mathrm{K_{myx}}} + 1} \\
v_{alt,10} &= \frac{\mathrm{k_d}\,\mathrm{C}\left(\mathrm{X}_3^{0,2,3} + \mathrm{X}_3^4\right)\left(\mathrm{Y}_3^{0,2,3} + \mathrm{Y}_3^4\right)}{\frac{\mathrm{Y}_3^{0,2,3} + \mathrm{Y}_3^4}{\mathrm{K_{mxy}}} + 1} - \frac{\mathrm{k_d}\,\mathrm{C}\,\mathrm{X}_3^{0,2,3}\,\mathrm{Y}_3^{0,2,3}}{\frac{\mathrm{Y}_3^{0,2,3}}{\mathrm{K_{mxy}}} + 1}
\end{aligned}$$

Layer Dynamics:

$$L(F^4|F^0, F^2, F^3) = \begin{bmatrix} X_1^4 & X_2^4 & X_3^4 & Y_1^4 & Y_2^4 & Y_3^4 \end{bmatrix}^T.$$

Layer $L(F^4|F^0, F^1, F^2, F^3)$

Required input trajectories:

$$\begin{bmatrix} X_1^{0,1,2,3} \\ X_2^{0,1,2,3} \\ X_3^{0,1,2,3} \\ Y_1^{0,1,2,3} \\ Y_2^{0,1,2,3} \\ Y_3^{0,1,2,3} \end{bmatrix} = \begin{bmatrix} 1 & 0 & 0 & 0 & 0 & 0 \\ 0 & 1 & 0 & 0 & 0 & 0 \\ 0 & 0 & 1 & 0 & 0 & 0 \\ 0 & 0 & 0 & 1 & 0 & 0 \\ 0 & 0 & 0 & 0 & 1 & 0 \\ 0 & 0 & 0 & 0 & 0 & 1 \end{bmatrix} L(F^0, F^1, F^2, F^3).$$

Ordinary differential equations:

$$\frac{d}{dt}\begin{bmatrix} X_1^4 \\ X_2^4 \\ X_3^4 \\ Y_1^4 \\ Y_2^4 \\ Y_3^4 \end{bmatrix} = \begin{bmatrix} -1 & 0 \\ 0 & 1 \\ 0 & 0 \\ 0 & -1 \\ 1 & 0 \\ 0 & 0 \end{bmatrix} \begin{bmatrix} v_7 \\ v_8 \end{bmatrix} + \begin{bmatrix} -1 & 1 & 0 & 0 & 0 & 0 & 0 & 0 \\ 1 & 0 & -1 & 0 & 0 & 0 & 0 & 0 \\ 0 & 0 & 1 & 0 & 0 & 0 & -1 & 0 \\ 0 & 0 & 0 & 1 & -1 & 0 & 0 & 0 \\ 0 & 0 & 0 & 0 & 1 & -1 & 0 & 0 \\ 0 & 0 & 0 & 0 & 0 & 1 & 0 & -1 \end{bmatrix} \begin{bmatrix} v_{alt,1} \\ v_{alt,2} \\ v_{alt,3} \\ v_{alt,4} \\ v_{alt,5} \\ v_{alt,6} \\ v_{alt,9} \\ v_{alt,10} \end{bmatrix},$$

with

$$\begin{aligned}
v_7 &= -\,\mathrm{k_a}\,\mathrm{C}\left(\mathrm{X}_1^{0,1,2,3} + \mathrm{X}_1^4\right)\left(\mathrm{Y}_2^{0,1,2,3} - \mathrm{Y}_{2\mathrm{T}} + \mathrm{Y}_2^4\right) \\
v_8 &= -\,\mathrm{k_a}\,\mathrm{C}\left(\mathrm{Y}_1^{0,1,2,3} + \mathrm{Y}_1^4\right)\left(\mathrm{X}_2^{0,1,2,3} - \mathrm{X}_{2\mathrm{T}} + \mathrm{X}_2^4\right) \\
v_{alt,1} &= -\,\mathrm{k_{12x}}\,\mathrm{C}\,\mathrm{X}_1^{0,1,2,3}\,\mathrm{X}_2^4 - \mathrm{k_{12x}}\,\mathrm{C}\,\mathrm{X}_1^4\left(\mathrm{X}_2^{0,1,2,3} - \mathrm{X}_{2\mathrm{T}} + \mathrm{X}_2^4\right) \\
v_{alt,2} &= -\,\frac{\mathrm{C}\,\mathrm{k_x}\,\mathrm{S}_1(\mathrm{t})\,\mathrm{K_{mx}}\,\mathrm{X}_1^4}{\mathrm{S}_1(\mathrm{t}) + \mathrm{K_{mx}}} \\
v_{alt,3} &= -\,\mathrm{k_{23x}}\,\mathrm{C}\,\mathrm{X}_2^{0,1,2,3}\,\mathrm{X}_3^4 - \mathrm{k_{23x}}\,\mathrm{C}\,\mathrm{X}_2^4\left(\mathrm{X}_3^{0,1,2,3} - \mathrm{X}_{3\mathrm{T}} + \mathrm{X}_3^4\right) \\
v_{alt,4} &= -\,\frac{\mathrm{S}_2(\mathrm{t})\,\mathrm{k_y}\,\mathrm{K_{my}}\,\mathrm{C}\,\mathrm{Y}_1^4}{\mathrm{S}_2(\mathrm{t}) + \mathrm{K_{my}}} \\
v_{alt,5} &= -\,\mathrm{k_{12y}}\,\mathrm{C}\,\mathrm{Y}_1^{0,1,2,3}\,\mathrm{Y}_2^4 - \mathrm{k_{12y}}\,\mathrm{C}\,\mathrm{Y}_1^4\left(\mathrm{Y}_2^{0,1,2,3} - \mathrm{Y}_{2\mathrm{T}} + \mathrm{Y}_2^4\right) \\
v_{alt,6} &= -\,\mathrm{k_{23y}}\,\mathrm{C}\,\mathrm{Y}_2^{0,1,2,3}\,\mathrm{Y}_3^4 - \mathrm{k_{23y}}\,\mathrm{C}\,\mathrm{Y}_2^4\left(\mathrm{Y}_3^{0,1,2,3} - \mathrm{Y}_{3\mathrm{T}} + \mathrm{Y}_3^4\right) \\
v_{alt,9} &= \frac{\mathrm{k_d}\,\mathrm{C}\left(\mathrm{X}_3^{0,1,2,3} + \mathrm{X}_3^4\right)\left(\mathrm{Y}_3^{0,1,2,3} + \mathrm{Y}_3^4\right)}{\frac{\mathrm{X}_3^{0,1,2,3} + \mathrm{X}_3^4}{\mathrm{K_{myx}}} + 1} - \frac{\mathrm{k_d}\,\mathrm{C}\,\mathrm{X}_3^{0,1,2,3}\,\mathrm{Y}_3^{0,1,2,3}}{\frac{\mathrm{X}_3^{0,1,2,3}}{\mathrm{K_{myx}}} + 1} \\
v_{alt,10} &= \frac{\mathrm{k_d}\,\mathrm{C}\left(\mathrm{X}_3^{0,1,2,3} + \mathrm{X}_3^4\right)\left(\mathrm{Y}_3^{0,1,2,3} + \mathrm{Y}_3^4\right)}{\frac{\mathrm{Y}_3^{0,1,2,3} + \mathrm{Y}_3^4}{\mathrm{K_{mxy}}} + 1} - \frac{\mathrm{k_d}\,\mathrm{C}\,\mathrm{X}_3^{0,1,2,3}\,\mathrm{Y}_3^{0,1,2,3}}{\frac{\mathrm{Y}_3^{0,1,2,3}}{\mathrm{K_{mxy}}} + 1}
\end{aligned}$$

Layer Dynamics:

$$L(F^4|F^0, F^1, F^2, F^3) = \begin{bmatrix} X_1^4 & X_2^4 & X_3^4 & Y_1^4 & Y_2^4 & Y_3^4 \end{bmatrix}^T.$$

Models for Example 2

General Setup

Parameters:

$$
\begin{aligned}
p_a &= 1 & p_b &= 1 & k_1 &= 2 \\
k_2 &= 0.01 & m_1 &= 1 & m_2 &= 1 \\
k_a &= 2 & m_a &= 10 & k_{-a} &= 1 \\
m_{-a} &= 0.0001 & k_b &= 2 & m_b &= 10 \\
k_{-b} &= 1 & m_{-b} &= 0.0001 & &
\end{aligned}
$$

Initial Conditions/"Zero Layer Dynamics" $L(F^0)$**:**

The initial conditions correspond to the steady state for $k = 3$.

$$
L(F^0) = \begin{bmatrix} y_1(t=0) \\ y_2(t=0) \\ a(t=0) \\ b(t=0) \end{bmatrix} = \begin{bmatrix} 10.0 \\ 10.0 \\ 0.5 \\ 0.8 \end{bmatrix}
$$

Inputs:

$$
k(t) = \begin{cases} 3 & 0 \le t < 50, \\ 10 & 50 \le t. \end{cases}
$$

Layer $L(F^1|F^0)$

Required input trajectories:

$$
\begin{bmatrix} y_1^0 \\ y_2^0 \\ a^0 \\ b^0 \end{bmatrix} = \begin{bmatrix} 1 & 0 & 0 & 0 \\ 0 & 1 & 0 & 0 \\ 0 & 0 & 1 & 0 \\ 0 & 0 & 0 & 1 \end{bmatrix} L(F^0).
$$

Ordinary differential equations:

$$
\frac{d}{dt} \begin{bmatrix} y_1^1 \\ y_2^1 \end{bmatrix} = \begin{bmatrix} 1 & -1 & 0 & -1 & 0 \\ 0 & 1 & -1 & 0 & -1 \end{bmatrix} \begin{bmatrix} v_1 \\ v_2 \\ v_3 \\ v_4 \\ v_7 \end{bmatrix},
$$

with

$$
\begin{aligned}
v_1 =& \frac{\mathrm{k(t)}}{\mathrm{p_a + a^0}} \\
v_2 =& \frac{\mathrm{k_1}\left(\mathrm{y_1^0 + y_1^1}\right)}{\left(\mathrm{p_b + b^0}\right)\left(\mathrm{m_1 + y_1^0 + y_1^1}\right)} \\
v_3 =& \frac{\mathrm{k_2}\left(\mathrm{y_2^0 + y_2^1}\right)}{\mathrm{m_2 + y_2^0 + y_2^1}}
\end{aligned}
$$

$$v_4 = \frac{\mathrm{k_a}\,\left(\mathrm{y}_1^0 + \mathrm{y}_1^1\right)}{\mathrm{m_a} + \mathrm{y}_1^0 + \mathrm{y}_1^1}$$

$$v_7 = \frac{\mathrm{k_b}\,\left(\mathrm{y}_2^0 + \mathrm{y}_2^1\right)}{\mathrm{m_b} + \mathrm{y}_2^0 + \mathrm{y}_2^1}$$

Layer Dynamics:

$$L(F^1|F^0) = \begin{bmatrix} y_1^1 & y_2^1 & 0 & 0 \end{bmatrix}^T.$$

Layer $L(F^1|F^0, F^2)$

Required input trajectories:

$$\begin{bmatrix} y_1^{0,2} \\ y_2^{0,2} \\ a^{0,2} \\ b^{0,2} \end{bmatrix} = \begin{bmatrix} 1 & 0 & 0 & 0 \\ 0 & 1 & 0 & 0 \\ 0 & 0 & 1 & 0 \\ 0 & 0 & 0 & 1 \end{bmatrix} L(F^0, F^2).$$

Ordinary differential equations:

$$\frac{d}{dt}\begin{bmatrix} y_1^1 \\ y_2^1 \\ a^1 \end{bmatrix} = \begin{bmatrix} 1 & -1 & 0 & -1 & 0 \\ 0 & 1 & -1 & 0 & -1 \\ 0 & 0 & 0 & 0 & 0 \end{bmatrix} \begin{bmatrix} v_1 \\ v_2 \\ v_3 \\ v_4 \\ v_7 \end{bmatrix} + \begin{bmatrix} 0 & 0 \\ 0 & 0 \\ 1 & -1 \end{bmatrix} \begin{bmatrix} v_{alt,5} \\ v_{alt,6} \end{bmatrix},$$

with

$$v_1 = \frac{\mathrm{k(t)}}{\mathrm{p_a} + \mathrm{a}^{0,2} + \mathrm{a}^1}$$

$$v_2 = \frac{\mathrm{k_1}\,\left(\mathrm{y}_1^{0,2} + \mathrm{y}_1^1\right)}{\left(\mathrm{p_b} + \mathrm{b}^{0,2}\right)\,\left(\mathrm{m_1} + \mathrm{y}_1^{0,2} + \mathrm{y}_1^1\right)}$$

$$v_3 = \frac{\mathrm{k_2}\,\left(\mathrm{y}_2^{0,2} + \mathrm{y}_2^1\right)}{\mathrm{m_2} + \mathrm{y}_2^{0,2} + \mathrm{y}_2^1}$$

$$v_4 = \frac{\mathrm{k_a}\,\left(\mathrm{y}_1^{0,2} + \mathrm{y}_1^1\right)}{\mathrm{m_a} + \mathrm{y}_1^{0,2} + \mathrm{y}_1^1}$$

$$v_7 = \frac{\mathrm{k_b}\,\left(\mathrm{y}_2^{0,2} + \mathrm{y}_2^1\right)}{\mathrm{m_b} + \mathrm{y}_2^{0,2} + \mathrm{y}_2^1}$$

$$v_{alt,5} = \frac{\mathrm{k_a}\,\mathrm{m_a}\,\mathrm{y}_1^1}{\left(\mathrm{m_a} + \mathrm{y}_1^{0,2}\right)\,\left(\mathrm{m_a} + \mathrm{y}_1^{0,2} + \mathrm{y}_1^1\right)}$$

$$v_{alt,6} = \frac{\mathrm{m_{-a}}\,\mathrm{k_{-a}}\,\mathrm{a}^1}{\left(\mathrm{m_{-a}} + \mathrm{a}^{0,2}\right)\,\left(\mathrm{m_{-a}} + \mathrm{a}^{0,2} + \mathrm{a}^1\right)}$$

Layer Dynamics:

$$L(F^1|F^0, F^2) = \begin{bmatrix} y_1^1 & y_2^1 & a^1 & 0 \end{bmatrix}^T.$$

Layer $L(F^1|F^0, F^3)$

Required input trajectories:

$$\begin{bmatrix} y_1^{0,3} \\ y_2^{0,3} \\ a^{0,3} \\ b^{0,3} \end{bmatrix} = \begin{bmatrix} 1 & 0 & 0 & 0 \\ 0 & 1 & 0 & 0 \\ 0 & 0 & 1 & 0 \\ 0 & 0 & 0 & 1 \end{bmatrix} L(F^0, F^3).$$

Ordinary differential equations:

$$\frac{d}{dt}\begin{bmatrix} y_1^1 \\ y_2^1 \\ b^1 \end{bmatrix} = \begin{bmatrix} 1 & -1 & 0 & -1 & 0 \\ 0 & 1 & -1 & 0 & -1 \\ 0 & 0 & 0 & 0 & 0 \end{bmatrix} \begin{bmatrix} v_1 \\ v_2 \\ v_3 \\ v_4 \\ v_7 \end{bmatrix} + \begin{bmatrix} 0 & 0 \\ 0 & 0 \\ 1 & -1 \end{bmatrix} \begin{bmatrix} v_{alt,8} \\ v_{alt,9} \end{bmatrix},$$

with

$$\begin{aligned}
v_1 &= \frac{\mathrm{k(t)}}{\mathrm{p_a} + \mathrm{a}^{0,3}} \\
v_2 &= \frac{\mathrm{k_1}\left(\mathrm{y}_1^{0,3} + \mathrm{y}_1^1\right)}{\left(\mathrm{m_1} + \mathrm{y}_1^{0,3} + \mathrm{y}_1^1\right)\left(\mathrm{p_b} + \mathrm{b}^{0,3} + \mathrm{b}^1\right)} \\
v_3 &= \frac{\mathrm{k_2}\left(\mathrm{y}_2^{0,3} + \mathrm{y}_2^1\right)}{\mathrm{m_2} + \mathrm{y}_2^{0,3} + \mathrm{y}_2^1} \\
v_4 &= \frac{\mathrm{k_a}\left(\mathrm{y}_1^{0,3} + \mathrm{y}_1^1\right)}{\mathrm{m_a} + \mathrm{y}_1^{0,3} + \mathrm{y}_1^1} \\
v_7 &= \frac{\mathrm{k_b}\left(\mathrm{y}_2^{0,3} + \mathrm{y}_2^1\right)}{\mathrm{m_b} + \mathrm{y}_2^{0,3} + \mathrm{y}_2^1} \\
v_{alt,8} &= \frac{\mathrm{k_b}\,\mathrm{m_b}\,\mathrm{y}_2^1}{\left(\mathrm{m_b} + \mathrm{y}_2^{0,3}\right)\left(\mathrm{m_b} + \mathrm{y}_2^{0,3} + \mathrm{y}_2^1\right)} \\
v_{alt,9} &= \frac{\mathrm{k_{-b}}\,\mathrm{m_{-b}}\,\mathrm{b}^1}{\left(\mathrm{m_{-b}} + \mathrm{b}^{0,3}\right)\left(\mathrm{m_{-b}} + \mathrm{b}^{0,3} + \mathrm{b}^1\right)}
\end{aligned}$$

Layer Dynamics:

$$L(F^1|F^0, F^3) = \begin{bmatrix} y_1^1 & y_2^1 & 0 & b^1 \end{bmatrix}^T.$$

Layer $L(F^1|F^0, F^2, F^3)$

Required input trajectories:

$$\begin{bmatrix} y_1^{0,2,3} \\ y_2^{0,2,3} \\ a^{0,2,3} \\ b^{0,2,3} \end{bmatrix} = \begin{bmatrix} 1 & 0 & 0 & 0 \\ 0 & 1 & 0 & 0 \\ 0 & 0 & 1 & 0 \\ 0 & 0 & 0 & 1 \end{bmatrix} L(F^0, F^2, F^3).$$

Ordinary differential equations:

$$\frac{d}{dt}\begin{bmatrix} y_1^1 \\ y_2^1 \\ a^1 \\ b^1 \end{bmatrix} = \begin{bmatrix} 1 & -1 & 0 & -1 & 0 \\ 0 & 1 & -1 & 0 & -1 \\ 0 & 0 & 0 & 0 & 0 \\ 0 & 0 & 0 & 0 & 0 \end{bmatrix} \begin{bmatrix} v_1 \\ v_2 \\ v_3 \\ v_4 \\ v_7 \end{bmatrix} + \begin{bmatrix} 0 & 0 & 0 & 0 \\ 0 & 0 & 0 & 0 \\ 1 & -1 & 0 & 0 \\ 0 & 0 & 1 & -1 \end{bmatrix} \begin{bmatrix} v_{alt,5} \\ v_{alt,6} \\ v_{alt,8} \\ v_{alt,9} \end{bmatrix},$$

with

$$\begin{aligned}
v_1 =& \frac{\mathrm{k(t)}}{\mathrm{p_a} + \mathrm{a}^{0,2,3} + \mathrm{a}^1} \\
v_2 =& \frac{\mathrm{k_1}\left(\mathrm{y}_1^{0,2,3} + \mathrm{y}_1^1\right)}{\left(\mathrm{m_1} + \mathrm{y}_1^{0,2,3} + \mathrm{y}_1^1\right)\left(\mathrm{p_b} + \mathrm{b}^{0,2,3} + \mathrm{b}^1\right)} \\
v_3 =& \frac{\mathrm{k_2}\left(\mathrm{y}_2^{0,2,3} + \mathrm{y}_2^1\right)}{\mathrm{m_2} + \mathrm{y}_2^{0,2,3} + \mathrm{y}_2^1} \\
v_4 =& \frac{\mathrm{k_a}\left(\mathrm{y}_1^{0,2,3} + \mathrm{y}_1^1\right)}{\mathrm{m_a} + \mathrm{y}_1^{0,2,3} + \mathrm{y}_1^1} \\
v_7 =& \frac{\mathrm{k_b}\left(\mathrm{y}_2^{0,2,3} + \mathrm{y}_2^1\right)}{\mathrm{m_b} + \mathrm{y}_2^{0,2,3} + \mathrm{y}_2^1} \\
v_{alt,5} =& \frac{\mathrm{k_a}\,\mathrm{m_a}\,\mathrm{y}_1^1}{\left(\mathrm{m_a} + \mathrm{y}_1^{0,2,3}\right)\left(\mathrm{m_a} + \mathrm{y}_1^{0,2,3} + \mathrm{y}_1^1\right)} \\
v_{alt,6} =& \frac{\mathrm{m_{-a}}\,\mathrm{k_{-a}}\,\mathrm{a}^1}{\left(\mathrm{m_{-a}} + \mathrm{a}^{0,2,3}\right)\left(\mathrm{m_{-a}} + \mathrm{a}^{0,2,3} + \mathrm{a}^1\right)} \\
v_{alt,8} =& \frac{\mathrm{k_b}\,\mathrm{m_b}\,\mathrm{y}_2^1}{\left(\mathrm{m_b} + \mathrm{y}_2^{0,2,3}\right)\left(\mathrm{m_b} + \mathrm{y}_2^{0,2,3} + \mathrm{y}_2^1\right)} \\
v_{alt,9} =& \frac{\mathrm{k_{-b}}\,\mathrm{m_{-b}}\,\mathrm{b}^1}{\left(\mathrm{m_{-b}} + \mathrm{b}^{0,2,3}\right)\left(\mathrm{m_{-b}} + \mathrm{b}^{0,2,3} + \mathrm{b}^1\right)}
\end{aligned}$$

Layer Dynamics:

$$L(F^1|F^0, F^2, F^3) = \begin{bmatrix} y_1^1 & y_2^1 & a^1 & b^1 \end{bmatrix}^T.$$

Layer $L(F^2|F^0)$

Required input trajectories:

$$\begin{bmatrix} y_1^0 \\ a^0 \end{bmatrix} = \begin{bmatrix} 1 & 0 & 0 & 0 \\ 0 & 0 & 1 & 0 \end{bmatrix} L(F^0).$$

Ordinary differential equations:

$$\frac{d}{dt}\begin{bmatrix} a^2 \end{bmatrix} = \begin{bmatrix} 1 & -1 \end{bmatrix} \begin{bmatrix} v_5 \\ v_6 \end{bmatrix},$$

with

$$v_5 = \frac{\mathrm{k_a\, y_1^0}}{\mathrm{m_a + y_1^0}}$$
$$v_6 = \frac{\mathrm{k_{-a}\, \left(a^0 + a^2\right)}}{\mathrm{m_{-a} + a^0 + a^2}}$$

Layer Dynamics:

$$L(F^2|F^0) = \begin{bmatrix}0 & 0 & a^2 & 0\end{bmatrix}^T.$$

Layer $L(F^2|F^0, F^1)$

Required input trajectories:

$$\begin{bmatrix}y_1^{0,1}\\ y_2^{0,1}\\ a^{0,1}\\ b^{0,1}\end{bmatrix} = \begin{bmatrix}1 & 0 & 0 & 0\\ 0 & 1 & 0 & 0\\ 0 & 0 & 1 & 0\\ 0 & 0 & 0 & 1\end{bmatrix} L(F^0, F^1).$$

Ordinary differential equations:

$$\frac{d}{dt}\begin{bmatrix}y_1^2\\ y_2^2\\ a^2\end{bmatrix} = \begin{bmatrix}0 & 0\\ 0 & 0\\ 1 & -1\end{bmatrix}\begin{bmatrix}v_5\\ v_6\end{bmatrix} + \begin{bmatrix}1 & -1 & 0 & -1 & 0\\ 0 & 1 & -1 & 0 & -1\\ 0 & 0 & 0 & 0 & 0\end{bmatrix}\begin{bmatrix}v_{alt,1}\\ v_{alt,2}\\ v_{alt,3}\\ v_{alt,4}\\ v_{alt,7}\end{bmatrix},$$

with

$$v_5 = \frac{\mathrm{k_a}\left(\mathrm{y_1^{0,1} + y_1^2}\right)}{\mathrm{m_a + y_1^{0,1} + y_1^2}}$$
$$v_6 = \frac{\mathrm{k_{-a}}\left(\mathrm{a^{0,1} + a^2}\right)}{\mathrm{m_{-a} + a^{0,1} + a^2}}$$
$$v_{alt,1} = -\frac{\mathrm{k(t)\, a^2}}{\mathrm{(p_a + a^{0,1})\,(p_a + a^{0,1} + a^2)}}$$
$$v_{alt,2} = \frac{\mathrm{k_1\, m_1\, y_1^2}}{\mathrm{(p_b + b^{0,1})}\left(\mathrm{m_1 + y_1^{0,1}}\right)\left(\mathrm{m_1 + y_1^{0,1} + y_1^2}\right)}$$
$$v_{alt,3} = \frac{\mathrm{k_2\, m_2\, y_2^2}}{\left(\mathrm{m_2 + y_2^{0,1}}\right)\left(\mathrm{m_2 + y_2^{0,1} + y_2^2}\right)}$$
$$v_{alt,4} = \frac{\mathrm{k_a\, m_a\, y_1^2}}{\left(\mathrm{m_a + y_1^{0,1}}\right)\left(\mathrm{m_a + y_1^{0,1} + y_1^2}\right)}$$
$$v_{alt,7} = \frac{\mathrm{k_b\, m_b\, y_2^2}}{\left(\mathrm{m_b + y_2^{0,1}}\right)\left(\mathrm{m_b + y_2^{0,1} + y_2^2}\right)}$$

Layer Dynamics:

$$L(F^2|F^0, F^1) = \begin{bmatrix} y_1^2 & y_2^2 & a^2 & 0 \end{bmatrix}^T.$$

Layer $L(F^2|F^0, F^3)$

Required input trajectories:

$$\begin{bmatrix} y_1^{0,3} \\ a^{0,3} \end{bmatrix} = \begin{bmatrix} 1 & 0 & 0 & 0 \\ 0 & 0 & 1 & 0 \end{bmatrix} L(F^0, F^3).$$

Ordinary differential equations:

$$\frac{d}{dt}\begin{bmatrix} a^2 \end{bmatrix} = \begin{bmatrix} 1 & -1 \end{bmatrix}\begin{bmatrix} v_5 \\ v_6 \end{bmatrix},$$

with

$$v_5 = \frac{\mathrm{k_a}\,\mathrm{y}_1^{0,3}}{\mathrm{m_a} + \mathrm{y}_1^{0,3}}$$
$$v_6 = \frac{\mathrm{k_{-a}}\left(\mathrm{a}^{0,3} + \mathrm{a}^2\right)}{\mathrm{m_{-a}} + \mathrm{a}^{0,3} + \mathrm{a}^2}$$

Layer Dynamics:

$$L(F^2|F^0, F^3) = \begin{bmatrix} 0 & 0 & a^2 & 0 \end{bmatrix}^T.$$

Layer $L(F^2|F^0, F^1, F^3)$

Required input trajectories:

$$\begin{bmatrix} y_1^{0,1,3} \\ y_2^{0,1,3} \\ a^{0,1,3} \\ b^{0,1,3} \end{bmatrix} = \begin{bmatrix} 1 & 0 & 0 & 0 \\ 0 & 1 & 0 & 0 \\ 0 & 0 & 1 & 0 \\ 0 & 0 & 0 & 1 \end{bmatrix} L(F^0, F^1, F^3).$$

Ordinary differential equations:

$$\frac{d}{dt}\begin{bmatrix} y_1^2 \\ y_2^2 \\ a^2 \\ b^2 \end{bmatrix} = \begin{bmatrix} 0 & 0 \\ 0 & 0 \\ 1 & -1 \\ 0 & 0 \end{bmatrix}\begin{bmatrix} v_5 \\ v_6 \end{bmatrix} + \begin{bmatrix} 1 & -1 & 0 & -1 & 0 & 0 & 0 \\ 0 & 1 & -1 & 0 & -1 & 0 & 0 \\ 0 & 0 & 0 & 0 & 0 & 0 & 0 \\ 0 & 0 & 0 & 0 & 0 & 1 & -1 \end{bmatrix}\begin{bmatrix} v_{alt,1} \\ v_{alt,2} \\ v_{alt,3} \\ v_{alt,4} \\ v_{alt,7} \\ v_{alt,8} \\ v_{alt,9} \end{bmatrix},$$

with

$$v_5 = \frac{\mathrm{k_a}\left(\mathrm{y}_1^{0,1,3} + \mathrm{y}_1^2\right)}{\mathrm{m_a} + \mathrm{y}_1^{0,1,3} + \mathrm{y}_1^2}$$
$$v_6 = \frac{\mathrm{k_{-a}}\left(\mathrm{a}^{0,1,3} + \mathrm{a}^2\right)}{\mathrm{m_{-a}} + \mathrm{a}^{0,1,3} + \mathrm{a}^2}$$

$$
\begin{aligned}
v_{alt,1} =& -\frac{\mathrm{k(t)\,a^2}}{(\mathrm{p_a + a^{0,1,3}})\,(\mathrm{p_a + a^{0,1,3} + a^2})}\\
v_{alt,2} =& \frac{\mathrm{k_1}\left(\mathrm{y_1^{0,1,3} + y_1^2}\right)}{\left(\mathrm{m_1 + y_1^{0,1,3} + y_1^2}\right)(\mathrm{p_b + b^{0,1,3} + b^2})} - \frac{\mathrm{k_1\,y_1^{0,1,3}}}{(\mathrm{p_b + b^{0,1,3}})\left(\mathrm{m_1 + y_1^{0,1,3}}\right)}\\
v_{alt,3} =& \frac{\mathrm{k_2\,m_2\,y_2^2}}{\left(\mathrm{m_2 + y_2^{0,1,3}}\right)\left(\mathrm{m_2 + y_2^{0,1,3} + y_2^2}\right)}\\
v_{alt,4} =& \frac{\mathrm{k_a\,m_a\,y_1^2}}{\left(\mathrm{m_a + y_1^{0,1,3}}\right)\left(\mathrm{m_a + y_1^{0,1,3} + y_1^2}\right)}\\
v_{alt,7} =& \frac{\mathrm{k_b\,m_b\,y_2^2}}{\left(\mathrm{m_b + y_2^{0,1,3}}\right)\left(\mathrm{m_b + y_2^{0,1,3} + y_2^2}\right)}\\
v_{alt,8} =& \frac{\mathrm{k_b\,m_b\,y_2^2}}{\left(\mathrm{m_b + y_2^{0,1,3}}\right)\left(\mathrm{m_b + y_2^{0,1,3} + y_2^2}\right)}\\
v_{alt,9} =& \frac{\mathrm{k_{-b}\,m_{-b}\,b^2}}{(\mathrm{m_{-b} + b^{0,1,3}})\,(\mathrm{m_{-b} + b^{0,1,3} + b^2})}
\end{aligned}
$$

Layer Dynamics:

$$L(F^2|F^0, F^1, F^3) = \begin{bmatrix} y_1^2 & y_2^2 & a^2 & b^2 \end{bmatrix}^T.$$

Layer $L(F^3|F^0)$

Required input trajectories:

$$\begin{bmatrix} y_2^0 \\ b^0 \end{bmatrix} = \begin{bmatrix} 0 & 1 & 0 & 0 \\ 0 & 0 & 0 & 1 \end{bmatrix} L(F^0).$$

Ordinary differential equations:

$$\frac{d}{dt}\begin{bmatrix} b^3 \end{bmatrix} = \begin{bmatrix} 1 & -1 \end{bmatrix}\begin{bmatrix} v_8 \\ v_9 \end{bmatrix},$$

with

$$
\begin{aligned}
v_8 =& \frac{\mathrm{k_b\,y_2^0}}{\mathrm{m_b + y_2^0}}\\
v_9 =& \frac{\mathrm{k_{-b}}\left(\mathrm{b^0 + b^3}\right)}{\mathrm{m_{-b} + b^0 + b^3}}
\end{aligned}
$$

Layer Dynamics:

$$L(F^3|F^0) = \begin{bmatrix} 0 & 0 & 0 & b^3 \end{bmatrix}^T.$$

Layer $L(F^3|F^0,F^1)$

Required input trajectories:

$$\begin{bmatrix} y_1^{0,1} \\ y_2^{0,1} \\ b^{0,1} \end{bmatrix} = \begin{bmatrix} 1 & 0 & 0 & 0 \\ 0 & 1 & 0 & 0 \\ 0 & 0 & 0 & 1 \end{bmatrix} L(F^0,F^1).$$

Ordinary differential equations:

$$\frac{d}{dt}\begin{bmatrix} y_1^3 \\ y_2^3 \\ b^3 \end{bmatrix} = \begin{bmatrix} 0 & 0 \\ 0 & 0 \\ 1 & -1 \end{bmatrix}\begin{bmatrix} v_8 \\ v_9 \end{bmatrix} + \begin{bmatrix} -1 & 0 & -1 & 0 \\ 1 & -1 & 0 & -1 \\ 0 & 0 & 0 & 0 \end{bmatrix}\begin{bmatrix} v_{alt,2} \\ v_{alt,3} \\ v_{alt,4} \\ v_{alt,7} \end{bmatrix},$$

with

$$\begin{aligned}
v_8 =& \frac{\mathrm{k_b}\left(\mathrm{y}_2^{0,1}+\mathrm{y}_2^3\right)}{\mathrm{m_b}+\mathrm{y}_2^{0,1}+\mathrm{y}_2^3} \\
v_9 =& \frac{\mathrm{k_{-b}}\left(\mathrm{b}^{0,1}+\mathrm{b}^3\right)}{\mathrm{m_{-b}}+\mathrm{b}^{0,1}+\mathrm{b}^3} \\
v_{alt,2} =& \frac{\mathrm{k_1}\left(\mathrm{y}_1^{0,1}+\mathrm{y}_1^3\right)}{\left(\mathrm{m_1}+\mathrm{y}_1^{0,1}+\mathrm{y}_1^3\right)\left(\mathrm{p_b}+\mathrm{b}^{0,1}+\mathrm{b}^3\right)} - \frac{\mathrm{k_1}\,\mathrm{y}_1^{0,1}}{\left(\mathrm{p_b}+\mathrm{b}^{0,1}\right)\left(\mathrm{m_1}+\mathrm{y}_1^{0,1}\right)} \\
v_{alt,3} =& \frac{\mathrm{k_2}\,\mathrm{m_2}\,\mathrm{y}_2^3}{\left(\mathrm{m_2}+\mathrm{y}_2^{0,1}\right)\left(\mathrm{m_2}+\mathrm{y}_2^{0,1}+\mathrm{y}_2^3\right)} \\
v_{alt,4} =& \frac{\mathrm{k_a}\,\mathrm{m_a}\,\mathrm{y}_1^3}{\left(\mathrm{m_a}+\mathrm{y}_1^{0,1}\right)\left(\mathrm{m_a}+\mathrm{y}_1^{0,1}+\mathrm{y}_1^3\right)} \\
v_{alt,7} =& \frac{\mathrm{k_b}\,\mathrm{m_b}\,\mathrm{y}_2^3}{\left(\mathrm{m_b}+\mathrm{y}_2^{0,1}\right)\left(\mathrm{m_b}+\mathrm{y}_2^{0,1}+\mathrm{y}_2^3\right)}
\end{aligned}$$

Layer Dynamics:

$$L(F^3|F^0,F^1) = \begin{bmatrix} y_1^3 & y_2^3 & 0 & b^3 \end{bmatrix}^T.$$

Layer $L(F^3|F^0,F^2)$

Required input trajectories:

$$\begin{bmatrix} y_2^{0,2} \\ b^{0,2} \end{bmatrix} = \begin{bmatrix} 0 & 1 & 0 & 0 \\ 0 & 0 & 0 & 1 \end{bmatrix} L(F^0,F^2).$$

Ordinary differential equations:

$$\frac{d}{dt}\begin{bmatrix} b^3 \end{bmatrix} = \begin{bmatrix} 1 & -1 \end{bmatrix}\begin{bmatrix} v_8 \\ v_9 \end{bmatrix},$$

with

$$v_8 = \frac{\mathrm{k_b}\,\mathrm{y}_2^{0,2}}{\mathrm{m_b}+\mathrm{y}_2^{0,2}}$$

$$v_9 = \frac{\mathrm{k_{-b}}\ \left(\mathrm{b}^{0,2} + \mathrm{b}^3\right)}{\mathrm{m_{-b}} + \mathrm{b}^{0,2} + \mathrm{b}^3}$$

Layer Dynamics:

$$L(F^3|F^0, F^2) = \begin{bmatrix}0 & 0 & 0 & b^3\end{bmatrix}^T.$$

Layer $L(F^3|F^0, F^1, F^2)$

Required input trajectories:

$$\begin{bmatrix} y_1^{0,1,2} \\ y_2^{0,1,2} \\ a^{0,1,2} \\ b^{0,1,2} \end{bmatrix} = \begin{bmatrix} 1 & 0 & 0 & 0 \\ 0 & 1 & 0 & 0 \\ 0 & 0 & 1 & 0 \\ 0 & 0 & 0 & 1 \end{bmatrix} L(F^0, F^1, F^2).$$

Ordinary differential equations:

$$\frac{d}{dt}\begin{bmatrix} y_1^3 \\ y_2^3 \\ a^3 \\ b^3 \end{bmatrix} = \begin{bmatrix} 0 & 0 \\ 0 & 0 \\ 0 & 0 \\ 1 & -1 \end{bmatrix} \begin{bmatrix} v_8 \\ v_9 \end{bmatrix} + \begin{bmatrix} 1 & -1 & 0 & -1 & 0 & 0 & 0 \\ 0 & 1 & -1 & 0 & -1 & 0 & 0 \\ 0 & 0 & 0 & 0 & 0 & 1 & -1 \\ 0 & 0 & 0 & 0 & 0 & 0 & 0 \end{bmatrix} \begin{bmatrix} v_{alt,1} \\ v_{alt,2} \\ v_{alt,3} \\ v_{alt,4} \\ v_{alt,7} \\ v_{alt,5} \\ v_{alt,6} \end{bmatrix},$$

with

$$v_8 = \frac{\mathrm{k_b}\ \left(\mathrm{y}_2^{0,1,2} + \mathrm{y}_2^3\right)}{\mathrm{m_b} + \mathrm{y}_2^{0,1,2} + \mathrm{y}_2^3}$$

$$v_9 = \frac{\mathrm{k_{-b}}\ \left(\mathrm{b}^{0,1,2} + \mathrm{b}^3\right)}{\mathrm{m_{-b}} + \mathrm{b}^{0,1,2} + \mathrm{b}^3}$$

$$v_{alt,1} = -\frac{\mathrm{k(t)\,a^3}}{\left(\mathrm{p_a} + \mathrm{a}^{0,1,2}\right)\ \left(\mathrm{p_a} + \mathrm{a}^{0,1,2} + \mathrm{a}^3\right)}$$

$$v_{alt,2} = \frac{\mathrm{k_1}\ \left(\mathrm{y}_1^{0,1,2} + \mathrm{y}_1^3\right)}{\left(\mathrm{m_1} + \mathrm{y}_1^{0,1,2} + \mathrm{y}_1^3\right)\ \left(\mathrm{p_b} + \mathrm{b}^{0,1,2} + \mathrm{b}^3\right)} - \frac{\mathrm{k_1\,y}_1^{0,1,2}}{\left(\mathrm{p_b} + \mathrm{b}^{0,1,2}\right)\ \left(\mathrm{m_1} + \mathrm{y}_1^{0,1,2}\right)}$$

$$v_{alt,3} = \frac{\mathrm{k_2\,m_2\,y}_2^3}{\left(\mathrm{m_2} + \mathrm{y}_2^{0,1,2}\right)\ \left(\mathrm{m_2} + \mathrm{y}_2^{0,1,2} + \mathrm{y}_2^3\right)}$$

$$v_{alt,4} = \frac{\mathrm{k_a\,m_a\,y}_1^3}{\left(\mathrm{m_a} + \mathrm{y}_1^{0,1,2}\right)\ \left(\mathrm{m_a} + \mathrm{y}_1^{0,1,2} + \mathrm{y}_1^3\right)}$$

$$v_{alt,7} = \frac{\mathrm{k_b\,m_b\,y}_2^3}{\left(\mathrm{m_b} + \mathrm{y}_2^{0,1,2}\right)\ \left(\mathrm{m_b} + \mathrm{y}_2^{0,1,2} + \mathrm{y}_2^3\right)}$$

$$v_{alt,5} = \frac{\mathrm{k_a\,m_a\,y}_1^3}{\left(\mathrm{m_a} + \mathrm{y}_1^{0,1,2}\right)\ \left(\mathrm{m_a} + \mathrm{y}_1^{0,1,2} + \mathrm{y}_1^3\right)}$$

$$v_{alt,6} = \frac{\mathrm{m_{-a}\,k_{-a}\,a^3}}{\left(\mathrm{m_{-a}} + \mathrm{a}^{0,1,2}\right)\left(\mathrm{m_{-a}} + \mathrm{a}^{0,1,2} + \mathrm{a}^3\right)}$$

Layer Dynamics:

$$L(F^3|F^0, F^1, F^2) = \begin{bmatrix} y_1^3 & y_2^3 & a^3 & b^3 \end{bmatrix}^T .$$

Models for Example 3

General Setup

For simplicity, we will in the following only present the layer models for the ordering of the functionalities as shown in Figure 9C in the main text. The layer models for other orderings are accordingly. Note, that for the glycolysis example we apply a mixed layer structure. Below, this structure is explained in more detail.

Parameters:
The mixed flow glucose concentration was set to $10mM$ to prevent glycogenic oscillations. All other parameters were kept unmodified as compared to glycolysis model [11] available at the BioModels Database [10], Model 61. We refer to [11] for the units and the interpretation of the parameters.

$$\begin{array}{rlrlrl}
P &= 0 & CN_{X0} &= 5.6 & Glc_{X0} &= 10 \\
k_0 &= 0.048 & Y_{vol} &= 59 & V_{2f} &= 1014.96 \\
K_{2Glc} &= 1.7 & P_2 &= 1 & K_{2IG6P} &= 1.2 \\
K_{2IIG6P} &= 7.2 & V_{2r} &= 1014.96 & V_{3m} &= 51.7547 \\
K_{3DGlc} &= 0.37 & K_{3ATP} &= 0.1 & K_{3Glc} &= 0 \\
V_{4f} &= 496.042 & K_{4G6P} &= 0.8 & K_{4F6P} &= 0.15 \\
V_{4r} &= 496.042 & K_{4eq} &= 0.13 & V_{5m} &= 45.4327 \\
K_5 &= 0.021 & \kappa_5 &= 0.15 & V_{6f} &= 2207.82 \\
K_{6FBP} &= 0.3 & K_{6DHAP} &= 2 & K_{6eq} &= 0.081 \\
ratio_6 &= 5 & K_{6GAP} &= 4 & K_{6IGAP} &= 10 \\
V_{7f} &= 116.365 & K_{7DHAP} &= 1.23 & K_{7GAP} &= 1.27 \\
V_{7r} &= 116.365 & K_{7eq} &= 0.055 & V_{8f} &= 833.858 \\
K_{8GAP} &= 0.6 & K_{8NAD} &= 0.1 & K_{8BPG} &= 0.01 \\
K_{8NADH} &= 0.06 & V_{8r} &= 833.858 & K_{8eq} &= 0.0055 \\
k_{9f} &= 443866 & k_{9r} &= 1528.62 & V_{10m} &= 343.096 \\
K_{10PEP} &= 0.2 & K_{10ADP} &= 0.17 & V_{11m} &= 53.1328 \\
K_{11} &= 0.3 & V_{12m} &= 89.8023 & K_{12NADH} &= 0.1 \\
K_{12ACA} &= 0.71 & k_{13} &= 16.72 & V_{15m} &= 81.4797 \\
K_{15DHAP} &= 25 & K_{15INADH} &= 0.034 & K_{15INAD} &= 0.13 \\
K_{15NADH} &= 0.13 & k_{16} &= 1.9 & k_{18} &= 24.7 \\
k_{20} &= 0.00283828 & k_{22} &= 2.25932 & k_{23} &= 3.2076 \\
k_{24f} &= 432.9 & k_{24r} &= 133.333 & C_{ext} &= 1 \\
C_{cyt} &= 1 & & & &
\end{array}$$

Initial Conditions/"Zero Layer Dynamics" $L(F^0)$:

The initial conditions correspond to the steady state for $Glc_{X0} = 10mM$. Note, that the initial conditions of the cofactors do not belong to the zero layer dynamics, but to the control layer.

$$L(F^0) = \begin{bmatrix} GlcX(t=0) \\ Glc(t=0) \\ G6P(t=0) \\ F6P(t=0) \\ FBP(t=0) \\ GAP(t=0) \\ DHAP(t=0) \\ BPG(t=0) \\ PEP(t=0) \\ Pyr(t=0) \\ ACA(t=0) \\ EtOH(t=0) \\ EtOHX(t=0) \\ Glyc(t=0) \\ GlycX(t=0) \\ ACAX(t=0) \\ CNX(t=0) \end{bmatrix} = \begin{bmatrix} 0.232254 \\ 0.0214792 \\ 2.36752 \\ 0.287489 \\ 0.899253 \\ 0.0514396 \\ 1.25181 \\ 0.000153658 \\ 0.0223765 \\ 0.420792 \\ 0.590469 \\ 12.0157 \\ 10.2753 \\ 1.68753 \\ 0.677582 \\ 0.51278 \\ 5.4352 \end{bmatrix}$$

Control Layer

As explained in the main text, we established a mixed layer structure for the glycolysis example. In this structure, a control layer communicates with all layers in the cascade without being part of the cascade. From every layer in the cascade, the control layer receives the fluxes (consumption or production rates) of the cofactors NAD^+, $NADH$, AMP, ATP, and AMP. The control layer integrates these fluxes together with the ATP consumption of the cell ($ATP \to ADP$, Reaction 23 in [11]), and the flux due to the AK reaction ($ATP + AMP \leftrightarrow 2ADP$, Reaction 24). Finally, it sends the integrated cofactor concentrations (indicated by superscript C) back to the cascaded layers.

The ordinary differential equations describing the control layer are as follows:

$$\frac{d}{dt}\begin{bmatrix} ATP^C \\ ADP^C \\ NAD^C \\ NADH^C \\ AMP^C \end{bmatrix} = \sum_{i=1}^{N_L} V\left(F^i | F^0, \dots F^{i-1}\right) + \begin{bmatrix} -1 & -1 \\ 1 & 2 \\ 0 & 0 \\ 0 & 0 \\ 0 & -1 \end{bmatrix} \begin{bmatrix} v_{23} \\ v_{24} \end{bmatrix}$$

$$\begin{bmatrix} ATP^C(t=0) \\ ADP^C(t=0) \\ NAD^C(t=0) \\ NADH^C(t=0) \\ AMP^C(t=0) \end{bmatrix} = \begin{bmatrix} 2.09266 \\ 1.50429 \\ 0.730744 \\ 0.249256 \\ 0.333054 \end{bmatrix},$$

with

$$v_{23} = C_{cyt} k23 ATP^C$$
$$v_{24} = C_{cyt}\left(k24f AMP^C ATP^C - k24r (ADP^C)^2\right).$$

Since in the mixed layer structure the states of the control layer depend on the dynamics of the cascaded layers and *vice versa*, the control layer has to be simulated simultaneously to all layers in the cascade. However, the dynamics of the mixed layer structure do not depend on the ordering of the cascaded functionalities. Thus, the layering graph can be constructed as described in the main text (Section "Reducing Computational Burden") by determining the dynamics of the control layer for any ordering of the cascaded functionalities, and using these trajectories as additional inputs for the simulation of the cascaded layers corresponding to any ordering of the functionalities. Since for $Glc_{X0} = 10mM$ the system–including the cofactor concentrations represented by the states of the control layer–are at steady state, we integrated the layering graph by replacing the states of the control layer by their corresponding initial conditions.

Layer $L(F^1|F^0)$

Required input trajectories:

$$\begin{bmatrix} GlcX^0 \\ Glc^0 \\ G6P^0 \end{bmatrix} = \begin{bmatrix} 1 & 0 & 0 & 0 & 0 & 0 & 0 & 0 & 0 & 0 & 0 & 0 & 0 & 0 & 0 & 0 & 0 & 0 & 0 & 0 & 0 & 0 \\ 0 & 1 & 0 & 0 & 0 & 0 & 0 & 0 & 0 & 0 & 0 & 0 & 0 & 0 & 0 & 0 & 0 & 0 & 0 & 0 & 0 & 0 \\ 0 & 0 & 0 & 1 & 0 & 0 & 0 & 0 & 0 & 0 & 0 & 0 & 0 & 0 & 0 & 0 & 0 & 0 & 0 & 0 & 0 & 0 \end{bmatrix} L(F^0).$$

Required inputs from control layer:
ATP^C.

Ordinary differential equations:

$$\frac{d}{dt}\begin{bmatrix} GlcX^1 \\ Glc^1 \\ G6P^1 \end{bmatrix} = \begin{bmatrix} 1 & -1 & 0 & 0 \\ 0 & 59 & -1 & 0 \\ 0 & 0 & 1 & -1 \end{bmatrix} \begin{bmatrix} v_1 \\ v_2 \\ v_3 \\ v_{22} \end{bmatrix}$$

$$\begin{bmatrix} V^1\,[ATP] \\ V^1\,[ADP] \end{bmatrix} = \begin{bmatrix} 0 & 0 & -1 & -1 \\ 0 & 0 & 1 & 1 \end{bmatrix} \begin{bmatrix} v_1 \\ v_2 \\ v_3 \\ v_{22} \end{bmatrix},$$

with

$$v_1 = -\,\mathrm{k_0}\,\mathrm{C_{ext}}\left(\mathrm{GlcX}^0 - \mathrm{Glc_{X0}} + \mathrm{GlcX}^1\right)$$

$$v_2 = \frac{\mathrm{V_{2f}}\,\mathrm{C_{ext}}\left(\mathrm{GlcX}^0 + \mathrm{GlcX}^1\right)}{\mathrm{Y_{vol}}\,\mathrm{K_{2Glc}}\left(\frac{\mathrm{GlcX}^0+\mathrm{GlcX}^1}{\mathrm{K_{2Glc}}} + \frac{\left(\frac{\mathrm{P_2}\left(\mathrm{GlcX}^0+\mathrm{GlcX}^1\right)}{\mathrm{K_{2Glc}}}+1\right)\left(\frac{\mathrm{Glc}^0+\mathrm{Glc}^1}{\mathrm{K_{2Glc}}} + \frac{\mathrm{G6P}^0+\mathrm{G6P}^1}{\mathrm{K_{2IG6P}}} + \frac{\left(\mathrm{Glc}^0+\mathrm{Glc}^1\right)\left(\mathrm{G6P}^0+\mathrm{G6P}^1\right)}{\mathrm{K_{2IIG6P}}\,\mathrm{K_{2Glc}}}+1\right)}{\frac{\mathrm{P_2}\left(\mathrm{Glc}^0+\mathrm{Glc}^1\right)}{\mathrm{K_{2Glc}}}+1} + 1\right)}$$
$$- \frac{\mathrm{V_{2r}}\,\mathrm{C_{cyt}}\left(\mathrm{Glc}^0 + \mathrm{Glc}^1\right)}{\mathrm{Y_{vol}}\,\mathrm{K_{2Glc}}\left(\frac{\mathrm{Glc}^0+\mathrm{Glc}^1}{\mathrm{K_{2Glc}}} + \frac{\mathrm{G6P}^0+\mathrm{G6P}^1}{\mathrm{K_{2IG6P}}} + \frac{\left(\frac{\mathrm{P_2}\left(\mathrm{Glc}^0+\mathrm{Glc}^1\right)}{\mathrm{K_{2Glc}}}+1\right)\left(\frac{\mathrm{GlcX}^0+\mathrm{GlcX}^1}{\mathrm{K_{2Glc}}}+1\right)}{\frac{\mathrm{P_2}\left(\mathrm{GlcX}^0+\mathrm{GlcX}^1\right)}{\mathrm{K_{2Glc}}}+1} + \frac{\left(\mathrm{Glc}^0+\mathrm{Glc}^1\right)\left(\mathrm{G6P}^0+\mathrm{G6P}^1\right)}{\mathrm{K_{2IIG6P}}\,\mathrm{K_{2Glc}}} + 1\right)}$$

$$v_3 = \frac{\mathrm{V_{3m}}\,\mathrm{C_{cyt}}\,\mathrm{ATP^C}\left(\mathrm{Glc}^0 + \mathrm{Glc}^1\right)}{\mathrm{K_{3ATP}}\left(\mathrm{Glc}^0 + \mathrm{Glc}^1\right) + \mathrm{ATP^C}\left(\mathrm{Glc}^0 + \mathrm{Glc}^1\right) + \mathrm{K_{3DGlc}}\,\mathrm{K_{3ATP}} + \mathrm{K_{3Glc}}\,\mathrm{ATP^C}}$$

$$v_{22} = \mathrm{k_{22}}\,\mathrm{C_{cyt}}\,\mathrm{ATP^C}\left(\mathrm{G6P}^0 + \mathrm{G6P}^1\right)$$

Layer Dynamics:

$$L(F^1|F^0) = \begin{bmatrix} GlcX^1 & Glc^1 & G6P^1 & 0 & 0 & 0 & 0 & 0 & 0 & 0 & 0 & 0 & 0 & 0 & 0 & 0 & 0 \end{bmatrix}^T.$$

Fluxes to control layer:

$$V(F^1|F^0) = \begin{bmatrix} V^1\,[ATP] & V^1\,[ADP] & 0 & 0 & 0 \end{bmatrix}^T.$$

Layer $L(F^2|F^0, F^1)$

Required input trajectories:

$$\begin{bmatrix} GlcX^{0,1} \\ Glc^{0,1} \\ G6P^{0,1} \\ F6P^{0,1} \\ FBP^{0,1} \\ GAP^{0,1} \\ DHAP^{0,1} \\ Glyc^{0,1} \\ GlycX^{0,1} \end{bmatrix} = \begin{bmatrix}
1&0&0&0&0&0&0&0&0&0&0&0&0&0&0&0&0&0&0&0&0&0 \\
0&1&0&0&0&0&0&0&0&0&0&0&0&0&0&0&0&0&0&0&0&0 \\
0&0&0&1&0&0&0&0&0&0&0&0&0&0&0&0&0&0&0&0&0&0 \\
0&0&0&0&0&1&0&0&0&0&0&0&0&0&0&0&0&0&0&0&0&0 \\
0&0&0&0&0&0&1&0&0&0&0&0&0&0&0&0&0&0&0&0&0&0 \\
0&0&0&0&0&0&0&1&0&0&0&0&0&0&0&0&0&0&0&0&0&0 \\
0&0&0&0&0&0&0&0&1&0&0&0&0&0&0&0&0&0&0&0&0&0 \\
0&0&0&0&0&0&0&0&0&0&0&0&0&0&0&0&0&1&0&0&0&0 \\
0&0&0&0&0&0&0&0&0&0&0&0&0&0&0&0&0&0&1&0&0&0
\end{bmatrix} L(F^0, F^1).$$

Required inputs from control layer:
ATP^C, NAD^C, $NADH^C$, and AMP^C.

Ordinary differential equations:

$$\frac{d}{dt}\begin{bmatrix} GlcX^2 \\ Glc^2 \\ G6P^2 \\ F6P^2 \\ FBP^2 \\ GAP^2 \\ DHAP^2 \\ Glyc^2 \\ GlycX^2 \end{bmatrix} = \begin{bmatrix} 0 & 0 & 0 & 0 & 0 & 0 & 0 \\ 0 & 0 & 0 & 0 & 0 & 0 & 0 \\ -1 & 0 & 0 & 0 & 0 & 0 & 0 \\ 1 & -1 & 0 & 0 & 0 & 0 & 0 \\ 0 & 1 & -1 & 0 & 0 & 0 & 0 \\ 0 & 0 & 1 & 1 & 0 & 0 & 0 \\ 0 & 0 & 1 & -1 & -1 & 0 & 0 \\ 0 & 0 & 0 & 0 & 1 & -59 & 0 \\ 0 & 0 & 0 & 0 & 0 & 1 & -1 \end{bmatrix} \begin{bmatrix} v_4 \\ v_5 \\ v_6 \\ v_7 \\ v_{15} \\ v_{16} \\ v_{17} \end{bmatrix} + \begin{bmatrix} 1 & -1 & 0 & 0 \\ 0 & 59 & -1 & 0 \\ 0 & 0 & 1 & -1 \\ 0 & 0 & 0 & 0 \\ 0 & 0 & 0 & 0 \\ 0 & 0 & 0 & 0 \\ 0 & 0 & 0 & 0 \\ 0 & 0 & 0 & 0 \\ 0 & 0 & 0 & 0 \end{bmatrix} \begin{bmatrix} v_{alt,1} \\ v_{alt,2} \\ v_{alt,3} \\ v_{alt,22} \end{bmatrix}$$

$$\begin{bmatrix} V^2\,[ATP] \\ V^2\,[ADP] \\ V^2\,[NAD] \\ V^2\,[NADH] \end{bmatrix} = \begin{bmatrix} 0 & -1 & 0 & 0 & 0 & 0 & 0 \\ 0 & 1 & 0 & 0 & 0 & 0 & 0 \\ 0 & 0 & 0 & 0 & 1 & 0 & 0 \\ 0 & 0 & 0 & 0 & -1 & 0 & 0 \end{bmatrix} \begin{bmatrix} v_4 \\ v_5 \\ v_6 \\ v_7 \\ v_{15} \\ v_{16} \\ v_{17} \end{bmatrix} + \begin{bmatrix} 0 & 0 & -1 & -1 \\ 0 & 0 & 1 & 1 \\ 0 & 0 & 0 & 0 \\ 0 & 0 & 0 & 0 \end{bmatrix} \begin{bmatrix} v_{alt,1} \\ v_{alt,2} \\ v_{alt,3} \\ v_{alt,22} \end{bmatrix},$$

with

$$v_4 = -\frac{\mathrm{K_{4F6P}\,C_{cyt}}\left(\mathrm{V_{4r}\,F6P^{0,1} + V_{4r}\,F6P^2 - V_{4f}\,K_{4eq}\,G6P^{0,1} - V_{4f}\,K_{4eq}\,G6P^2}\right)}{\mathrm{K_{4eq}}\left(\mathrm{K_{4G6P}\,K_{4F6P} + K_{4F6P}\,G6P^{0,1} + K_{4F6P}\,G6P^2 + K_{4G6P}\,F6P^{0,1} + K_{4G6P}\,F6P^2}\right)}$$

$$v_5 = \frac{\mathrm{V_{5m}\,C_{cyt}}\left(\mathrm{F6P^{0,1} + F6P^2}\right)^2}{\mathrm{K_5}\left(\frac{\kappa_5\,\mathrm{ATP^{C^2}}}{\mathrm{AMP^{C^2}}} + 1\right) + \left(\mathrm{F6P^{0,1} + F6P^2}\right)^2}$$

$$v_6 = -\frac{\mathrm{V_{6f}\,ratio_6\,K_{6IGAP}\,C_{cyt}}\left(\mathrm{GAP^{0,1}\,DHAP^{0,1} - K_{6eq}\,FBP^2 - K_{6eq}\,FBP^{0,1} + GAP^{0,1}\,DHAP^2 + GAP^2\,DHAP^{0,1} + GAP^2\,DHAP^2}\right)}{\alpha_1 + \mathrm{K_{6GAP}\,K_{6IGAP}\,(DHAP^{0,1} + DHAP^2) + K_{6FBP}\,K_{6eq}\,ratio_6\,K_{6IGAP} + K_{6eq}\,ratio_6\,K_{6IGAP}\,(FBP^{0,1} + FBP^2)}}$$

$$\alpha_1 = \left(\mathrm{K_{6DHAP}\,K_{6IGAP} + K_{6IGAP}\,(DHAP^{0,1} + DHAP^2) + K_{6eq}\,ratio_6\,(FBP^{0,1} + FBP^2)}\right)\mathrm{(GAP^{0,1} + GAP^2)}$$

$$v_7 = -\frac{\mathrm{K_{7GAP}\,C_{cyt}}\left(\mathrm{V_{7r}\,GAP^{0,1} + V_{7r}\,GAP^2 - V_{7f}\,K_{7eq}\,DHAP^{0,1} - V_{7f}\,K_{7eq}\,DHAP^2}\right)}{\mathrm{K_{7eq}}\left(\mathrm{K_{7DHAP}\,K_{7GAP} + K_{7DHAP}\,GAP^{0,1} + K_{7DHAP}\,GAP^2 + K_{7GAP}\,DHAP^{0,1} + K_{7GAP}\,DHAP^2}\right)}$$

$$v_{15} = \frac{\mathrm{V_{15m}\,C_{cyt}}\left(\mathrm{DHAP^{0,1} + DHAP^2}\right)}{\left(\frac{\mathrm{K_{15NADH}}\left(\frac{\mathrm{NAD^C}}{\mathrm{K_{15INAD}}}+1\right)}{\mathrm{NADH^C}} + 1\right)\left(\mathrm{DHAP^{0,1} + DHAP^2}\right) + \mathrm{K_{15DHAP}}\left(\frac{\mathrm{K_{15INADH}}\left(\frac{\mathrm{NAD^C}}{\mathrm{K_{15INAD}}}+1\right)}{\mathrm{NADH^C}} + 1\right)}$$

$$v_{16} = \frac{\mathrm{k_{16}}\left(\mathrm{C_{cyt}}\left(\mathrm{Glyc^{0,1} + Glyc^2}\right) - \mathrm{C_{ext}}\left(\mathrm{GlycX^{0,1} + GlycX^2}\right)\right)}{\mathrm{Y_{vol}}}$$

$$v_{17} = \mathrm{k_0\,C_{ext}}\left(\mathrm{GlycX^{0,1} + GlycX^2}\right)$$

$$v_{alt,1} = -\,\mathrm{k_0\,C_{ext}\,GlcX^2}$$

$$v_{alt,2} = \frac{\mathrm{V_{2r}\,C_{cyt}\,Glc^{0,1}}}{\mathrm{Y_{vol}\,K_{2Glc}}\left(\frac{\mathrm{Glc^{0,1}}}{\mathrm{K_{2Glc}}} + \frac{\mathrm{G6P^{0,1}}}{\mathrm{K_{2IG6P}}} + \frac{\left(\frac{\mathrm{GlcX^{0,1}}}{\mathrm{K_{2Glc}}}+1\right)\left(\frac{\mathrm{P_2\,Glc^{0,1}}}{\mathrm{K_{2Glc}}}+1\right)}{\frac{\mathrm{P_2\,GlcX^{0,1}}}{\mathrm{K_{2Glc}}}+1} + \frac{\mathrm{Glc^{0,1}\,G6P^{0,1}}}{\mathrm{K_{2IIG6P}\,K_{2Glc}}} + 1\right)}$$
$$- \frac{\mathrm{V_{2f}\,C_{ext}\,GlcX^{0,1}}}{\mathrm{Y_{vol}\,K_{2Glc}}\left(\frac{\mathrm{GlcX^{0,1}}}{\mathrm{K_{2Glc}}} + \frac{\left(\frac{\mathrm{P_2\,GlcX^{0,1}}}{\mathrm{K_{2Glc}}}+1\right)\left(\frac{\mathrm{Glc^{0,1}}}{\mathrm{K_{2Glc}}} + \frac{\mathrm{G6P^{0,1}}}{\mathrm{K_{2IG6P}}} + \frac{\mathrm{Glc^{0,1}\,G6P^{0,1}}}{\mathrm{K_{2IIG6P}\,K_{2Glc}}}+1\right)}{\frac{\mathrm{P_2\,Glc^{0,1}}}{\mathrm{K_{2Glc}}}+1} + 1\right)}$$
$$- \frac{\mathrm{V_{2r}\,C_{cyt}}\left(\mathrm{Glc^{0,1} + Glc^2}\right)}{\mathrm{Y_{vol}\,K_{2Glc}}\left(\frac{\mathrm{Glc^{0,1}+Glc^2}}{\mathrm{K_{2Glc}}} + \frac{\mathrm{G6P^{0,1}+G6P^2}}{\mathrm{K_{2IG6P}}} + \frac{\left(\frac{\mathrm{P_2}\left(\mathrm{Glc^{0,1}+Glc^2}\right)}{\mathrm{K_{2Glc}}}+1\right)\left(\frac{\mathrm{GlcX^{0,1}+GlcX^2}}{\mathrm{K_{2Glc}}}+1\right)}{\frac{\mathrm{P_2}\left(\mathrm{GlcX^{0,1}+GlcX^2}\right)}{\mathrm{K_{2Glc}}}+1} + \frac{\left(\mathrm{Glc^{0,1}+Glc^2}\right)\left(\mathrm{G6P^{0,1}+G6P^2}\right)}{\mathrm{K_{2IIG6P}\,K_{2Glc}}} + 1\right)}$$
$$+ \frac{\mathrm{V_{2f}\,C_{ext}}\left(\mathrm{GlcX^{0,1} + GlcX^2}\right)}{\mathrm{Y_{vol}\,K_{2Glc}}\left(\frac{\mathrm{GlcX^{0,1}+GlcX^2}}{\mathrm{K_{2Glc}}} + \frac{\left(\frac{\mathrm{P_2}\left(\mathrm{GlcX^{0,1}+GlcX^2}\right)}{\mathrm{K_{2Glc}}}+1\right)\left(\frac{\mathrm{Glc^{0,1}+Glc^2}}{\mathrm{K_{2Glc}}} + \frac{\mathrm{G6P^{0,1}+G6P^2}}{\mathrm{K_{2IG6P}}} + \frac{\left(\mathrm{Glc^{0,1}+Glc^2}\right)\left(\mathrm{G6P^{0,1}+G6P^2}\right)}{\mathrm{K_{2IIG6P}\,K_{2Glc}}}+1\right)}{\frac{\mathrm{P_2}\left(\mathrm{Glc^{0,1}+Glc^2}\right)}{\mathrm{K_{2Glc}}}+1} + 1\right)}$$

$$v_{alt,3} = \frac{\mathrm{V_{3m}\,C_{cyt}\,ATP^C}\left(\mathrm{Glc^{0,1} + Glc^2}\right)}{\mathrm{K_{3ATP}}\left(\mathrm{Glc^{0,1} + Glc^2}\right) + \mathrm{ATP^C}\left(\mathrm{Glc^{0,1} + Glc^2}\right) + \mathrm{K_{3DGlc}\,K_{3ATP} + K_{3Glc}\,ATP^C}} - \frac{\mathrm{V_{3m}\,C_{cyt}\,Glc^{0,1}\,ATP^C}}{\mathrm{K_{3DGlc}\,K_{3ATP} + K_{3ATP}\,Glc^{0,1} + K_{3Glc}\,ATP^C + Glc^{0,1}\,ATP^C}}$$

$$v_{alt,22} = \mathrm{k_{22}\,C_{cyt}\,ATP^C\,G6P^2}$$

Layer Dynamics:

$$L(F^2|F^0, F^1) = \begin{bmatrix} GlcX^2 & Glc^2 & G6P^2 & F6P^2 & FBP^2 & GAP^2 & DHAP^2 & 0 & 0 & 0 & 0 & 0 & 0 & Glyc^2 & GlycX^2 & 0 & 0 \end{bmatrix}^T .$$

Fluxes to control layer:

$$V(F^2|F^0, F^1) = \begin{bmatrix} V^2\,[ATP] & V^2\,[ADP] & V^2\,[NAD] & V^2\,[NADH] & 0 \end{bmatrix}^T .$$

Layer $L(F^3|F^0, F^1, F^2)$

Required input trajectories:

$$\begin{bmatrix} FBP^{0,1,2} \\ GAP^{0,1,2} \\ DHAP^{0,1,2} \\ BPG^{0,1,2} \\ PEP^{0,1,2} \\ Pyr^{0,1,2} \\ ACA^{0,1,2} \\ EtOH^{0,1,2} \\ EtOHX^{0,1,2} \end{bmatrix} = \begin{bmatrix} 0&0&0&0&0&0&1&0&0&0&0&0&0&0&0&0&0&0&0&0&0&0 \\ 0&0&0&0&0&0&0&1&0&0&0&0&0&0&0&0&0&0&0&0&0&0 \\ 0&0&0&0&0&0&0&0&1&0&0&0&0&0&0&0&0&0&0&0&0&0 \\ 0&0&0&0&0&0&0&0&0&0&1&0&0&0&0&0&0&0&0&0&0&0 \\ 0&0&0&0&0&0&0&0&0&0&0&0&1&0&0&0&0&0&0&0&0&0 \\ 0&0&0&0&0&0&0&0&0&0&0&0&0&1&0&0&0&0&0&0&0&0 \\ 0&0&0&0&0&0&0&0&0&0&0&0&0&0&1&0&0&0&0&0&0&0 \\ 0&0&0&0&0&0&0&0&0&0&0&0&0&0&0&1&0&0&0&0&0&0 \\ 0&0&0&0&0&0&0&0&0&0&0&0&0&0&0&0&1&0&0&0&0&0 \end{bmatrix} L(F^0, F^1, F^2).$$

Required inputs from control layer:
ATP^C, ADP^C, NAD^C, and $NADH^C$.
Ordinary differential equations:

$$\frac{d}{dt}\begin{bmatrix} FBP^3 \\ GAP^3 \\ DHAP^3 \\ BPG^3 \\ PEP^3 \\ Pyr^3 \\ ACA^3 \\ EtOH^3 \\ EtOHX^3 \\ Glyc^3 \\ GlycX^3 \end{bmatrix} = \begin{bmatrix} 0&0&0&0&0&0&0 \\ -1&0&0&0&0&0&0 \\ 0&0&0&0&0&0&0 \\ 1&-1&0&0&0&0&0 \\ 0&1&-1&0&0&0&0 \\ 0&0&1&-1&0&0&0 \\ 0&0&0&1&-1&0&0 \\ 0&0&0&0&1&-59&0 \\ 0&0&0&0&0&1&-1 \\ 0&0&0&0&0&0&0 \\ 0&0&0&0&0&0&0 \end{bmatrix} \begin{bmatrix} v_8 \\ v_9 \\ v_{10} \\ v_{11} \\ v_{12} \\ v_{13} \\ v_{14} \end{bmatrix} + \begin{bmatrix} -1&0&0&0&0 \\ 1&1&0&0&0 \\ 1&-1&-1&0&0 \\ 0&0&0&0&0 \\ 0&0&0&0&0 \\ 0&0&0&0&0 \\ 0&0&0&0&0 \\ 0&0&0&0&0 \\ 0&0&0&0&0 \\ 0&0&1&-59&0 \\ 0&0&0&1&-1 \end{bmatrix} \begin{bmatrix} v_{alt,6} \\ v_{alt,7} \\ v_{alt,15} \\ v_{alt,16} \\ v_{alt,17} \end{bmatrix}$$

$$\begin{bmatrix} V^3\,[ATP] \\ V^3\,[ADP] \\ V^3\,[NAD] \\ V^3\,[NADH] \end{bmatrix} = \begin{bmatrix} 0&1&1&0&0&0&0 \\ 0&-1&-1&0&0&0&0 \\ -1&0&0&0&1&0&0 \\ 1&0&0&0&-1&0&0 \end{bmatrix} \begin{bmatrix} v_8 \\ v_9 \\ v_{10} \\ v_{11} \\ v_{12} \\ v_{13} \\ v_{14} \end{bmatrix} + \begin{bmatrix} 0&0&0&0&0 \\ 0&0&0&0&0 \\ 0&0&1&0&0 \\ 0&0&-1&0&0 \end{bmatrix} \begin{bmatrix} v_{alt,6} \\ v_{alt,7} \\ v_{alt,15} \\ v_{alt,16} \\ v_{alt,17} \end{bmatrix},$$

with

$$v_8 = -\frac{\mathrm{K_{8BPG}}\,\mathrm{K_{8NADH}}\,\mathrm{C_{cyt}}\left(\mathrm{V_{8r}}\,\mathrm{NADH^C}\,\mathrm{BPG^{0,1,2}} + \mathrm{V_{8r}}\,\mathrm{NADH^C}\,\mathrm{BPG^3} - \mathrm{V_{8f}}\,\mathrm{K_{8eq}}\,\mathrm{NAD^C}\,\mathrm{GAP^{0,1,2}} - \mathrm{V_{8f}}\,\mathrm{K_{8eq}}\,\mathrm{NAD^C}\,\mathrm{GAP^3}\right)}{\mathrm{K_{8eq}}\left(\mathrm{K_{8NAD}}\,\mathrm{K_{8NADH}} + \mathrm{K_{8NAD}}\,\mathrm{NADH^C} + \mathrm{K_{8NADH}}\,\mathrm{NAD^C}\right)\left(\mathrm{K_{8GAP}}\,\mathrm{K_{8BPG}} + \mathrm{K_{8GAP}}\,\mathrm{BPG^{0,1,2}} + \mathrm{K_{8GAP}}\,\mathrm{BPG^3} + \mathrm{K_{8BPG}}\,\mathrm{GAP^{0,1,2}} + \mathrm{K_{8BPG}}\,\mathrm{GAP^3}\right)}$$

$$v_9 = -\,\mathrm{C_{cyt}}\left(\mathrm{k_{9r}}\,\mathrm{ATP^C}\left(\mathrm{PEP^{0,1,2}} + \mathrm{PEP^3}\right) - \mathrm{k_{9f}}\,\mathrm{ADP^C}\left(\mathrm{BPG^{0,1,2}} + \mathrm{BPG^3}\right)\right)$$

$$v_{10} = \frac{\mathrm{V_{10m}}\,\mathrm{C_{cyt}}\,\mathrm{ADP^C}\left(\mathrm{PEP^{0,1,2}} + \mathrm{PEP^3}\right)}{\left(\mathrm{K_{10ADP}} + \mathrm{ADP^C}\right)\left(\mathrm{K_{10PEP}} + \mathrm{PEP^{0,1,2}} + \mathrm{PEP^3}\right)}$$

$$v_{11} = \frac{\mathrm{V_{11m}}\,\mathrm{C_{cyt}}\left(\mathrm{Pyr^{0,1,2}} + \mathrm{Pyr^3}\right)}{\mathrm{K_{11}} + \mathrm{Pyr^{0,1,2}} + \mathrm{Pyr^3}}$$

$$v_{12} = \frac{\mathrm{V_{12m}}\,\mathrm{C_{cyt}}\,\mathrm{NADH^C}\left(\mathrm{ACA^{0,1,2}} + \mathrm{ACA^3}\right)}{\left(\mathrm{K_{12NADH}} + \mathrm{NADH^C}\right)\left(\mathrm{K_{12ACA}} + \mathrm{ACA^{0,1,2}} + \mathrm{ACA^3}\right)}$$

$$v_{13} = \frac{\mathrm{k_{13}}\left(\mathrm{C_{cyt}}\left(\mathrm{EtOH^{0,1,2}} + \mathrm{EtOH^3}\right) - \mathrm{C_{ext}}\left(\mathrm{EtOHX^{0,1,2}} + \mathrm{EtOHX^3}\right)\right)}{\mathrm{Y_{vol}}}$$

$$v_{14} = \mathrm{k_0}\,\mathrm{C_{ext}}\left(\mathrm{EtOHX^{0,1,2}} + \mathrm{EtOHX^3}\right)$$

$$v_{alt,6} = \frac{\mathrm{C_{cyt}\,V_{6f}}\left(\mathrm{FBP^{0,1,2} + FBP^3} - \frac{\left(\mathrm{GAP^{0,1,2}+GAP^3}\right)\left(\mathrm{DHAP^{0,1,2}+DHAP^3}\right)}{\mathrm{K_{6eq}}}\right)}{\mathrm{K_{6FBP} + FBP^{0,1,2} + FBP^3} + \frac{\left(\mathrm{FBP^{0,1,2}+FBP^3}\right)\left(\mathrm{GAP^{0,1,2}+GAP^3}\right)}{\mathrm{K_{6IGAP}}} + \frac{\left(\mathrm{K_{6DHAP}+DHAP^{0,1,2}+DHAP^3}\right)\left(\mathrm{GAP^{0,1,2}+GAP^3}\right)+\mathrm{K_{6GAP}}\left(\mathrm{DHAP^{0,1,2}+DHAP^3}\right)}{\mathrm{K_{6eq}\,ratio_6}}}$$

$$- \frac{\mathrm{C_{cyt}\,V_{6f}\,FBP^{0,1,2}}}{\mathrm{K_{6FBP} + FBP^{0,1,2}} + \frac{\mathrm{FBP^{0,1,2}\,GAP^{0,1,2}}}{\mathrm{K_{6IGAP}}} + \frac{\mathrm{K_{6DHAP}\,GAP^{0,1,2}}}{\mathrm{K_{6eq}\,ratio_6}} + \frac{\mathrm{K_{6GAP}\,DHAP^{0,1,2}}}{\mathrm{K_{6eq}\,ratio_6}} + \frac{\mathrm{GAP^{0,1,2}\,DHAP^{0,1,2}}}{\mathrm{K_{6eq}\,ratio_6}}}$$

$$- \frac{\mathrm{C_{cyt}\,V_{6f}\,GAP^{0,1,2}\,DHAP^{0,1,2}}}{\mathrm{K_{6eq}}\left(\mathrm{K_{6FBP} + FBP^{0,1,2}} + \frac{\mathrm{FBP^{0,1,2}\,GAP^{0,1,2}}}{\mathrm{K_{6IGAP}}} + \frac{\mathrm{K_{6DHAP}\,GAP^{0,1,2}}}{\mathrm{K_{6eq}\,ratio_6}} + \frac{\mathrm{K_{6GAP}\,DHAP^{0,1,2}}}{\mathrm{K_{6eq}\,ratio_6}} + \frac{\mathrm{GAP^{0,1,2}\,DHAP^{0,1,2}}}{\mathrm{K_{6eq}\,ratio_6}}\right)}$$

$$v_{alt,7} = \frac{\mathrm{K_{7GAP}\,C_{cyt}}\left(\mathrm{V_{7f}\,K_{7eq}\,DHAP^{0,1,2} + V_{7f}\,K_{7eq}\,DHAP^3 - V_{7r}\,GAP^{0,1,2} - V_{7r}\,GAP^3}\right)}{\mathrm{K_{7eq}}\left(\mathrm{K_{7DHAP}\,K_{7GAP} + K_{7DHAP}\,GAP^{0,1,2} + K_{7DHAP}\,GAP^3 + K_{7GAP}\,DHAP^{0,1,2} + K_{7GAP}\,DHAP^3}\right)}$$

$$- \frac{\mathrm{K_{7GAP}\,C_{cyt}}\left(\mathrm{V_{7f}\,K_{7eq}\,DHAP^{0,1,2} - V_{7r}\,GAP^{0,1,2}}\right)}{\mathrm{K_{7eq}}\left(\mathrm{K_{7DHAP}\,K_{7GAP} + K_{7DHAP}\,GAP^{0,1,2} + K_{7GAP}\,DHAP^{0,1,2}}\right)}$$

$$v_{alt,15} = \frac{\mathrm{V_{15m}\,C_{cyt}}\left(\mathrm{DHAP^{0,1,2} + DHAP^3}\right)}{\left(\frac{\mathrm{K_{15NADH}}\left(\frac{\mathrm{NAD^C}}{\mathrm{K_{15INAD}}}+1\right)}{\mathrm{NADH^C}} + 1\right)\left(\mathrm{DHAP^{0,1,2} + DHAP^3}\right) + \mathrm{K_{15DHAP}}\left(\frac{\mathrm{K_{15INADH}}\left(\frac{\mathrm{NAD^C}}{\mathrm{K_{15INAD}}}+1\right)}{\mathrm{NADH^C}} + 1\right)}$$

$$- \frac{\mathrm{V_{15m}\,C_{cyt}\,DHAP^{0,1,2}}}{\mathrm{K_{15DHAP}}\left(\frac{\mathrm{K_{15INADH}}\left(\frac{\mathrm{NAD^C}}{\mathrm{K_{15INAD}}}+1\right)}{\mathrm{NADH^C}} + 1\right) + \mathrm{DHAP^{0,1,2}}\left(\frac{\mathrm{K_{15NADH}}\left(\frac{\mathrm{NAD^C}}{\mathrm{K_{15INAD}}}+1\right)}{\mathrm{NADH^C}} + 1\right)}$$

$$v_{alt,16} = -\frac{\mathrm{k_{16}}\left(\mathrm{C_{ext}\,GlycX^3 - C_{cyt}\,Glyc^3}\right)}{\mathrm{Y_{vol}}}$$

$$v_{alt,17} = \mathrm{k_0\,C_{ext}\,GlycX^3}$$

Layer Dynamics:

$$L(F^3|F^0, F^1, F^2) = \begin{bmatrix} 0 & 0 & 0 & 0 & FBP^3 & GAP^3 & DHAP^3 & BPG^3 & PEP^3 & Pyr^3 & ACA^3 & EtOH^3 & EtOHX^3 & Glyc^3 & GlycX^3 & 0 & 0 \end{bmatrix}^T.$$

Fluxes to control layer:

$$V(F^3|F^0, F^1, F^2) = \begin{bmatrix} V^3[ATP] & V^3[ADP] & V^3[NAD] & V^3[NADH] & 0 \end{bmatrix}^T.$$

Layer $L(F^4|F^0, F^1, F^2, F^3)$

Required input trajectories:

$$\begin{bmatrix} ACA^{0,1,2,3} \\ ACAX^{0,1,2,3} \end{bmatrix} = \begin{bmatrix} 0 & 0 & 0 & 0 & 0 & 0 & 0 & 0 & 0 & 0 & 0 & 0 & 0 & 0 & 1 & 0 & 0 & 0 & 0 & 0 & 0 & 0 \\ 0 & 0 & 0 & 0 & 0 & 0 & 0 & 0 & 0 & 0 & 0 & 0 & 0 & 0 & 0 & 0 & 0 & 0 & 0 & 1 & 0 & 0 \end{bmatrix} L(F^0, F^1, F^2, F^3).$$

Required inputs from control layer:
$NADH^C$.

Ordinary differential equations:

$$\frac{d}{dt}\begin{bmatrix} ACA^4 \\ EtOH^4 \\ EtOHX^4 \\ ACAX^4 \end{bmatrix} = \begin{bmatrix} -59 & 0 \\ 0 & 0 \\ 0 & 0 \\ 1 & -1 \end{bmatrix}\begin{bmatrix} v_{18} \\ v_{19} \end{bmatrix} + \begin{bmatrix} -1 & 0 & 0 \\ 1 & -59 & 0 \\ 0 & 1 & -1 \\ 0 & 0 & 0 \end{bmatrix}\begin{bmatrix} v_{alt,12} \\ v_{alt,13} \\ v_{alt,14} \end{bmatrix}$$

$$\begin{bmatrix} V^4[NAD] \\ V^4[NADH] \end{bmatrix} = \begin{bmatrix} 0 & 0 \\ 0 & 0 \end{bmatrix}\begin{bmatrix} v_{18} \\ v_{19} \end{bmatrix} + \begin{bmatrix} 1 & 0 & 0 \\ -1 & 0 & 0 \end{bmatrix}\begin{bmatrix} v_{alt,12} \\ v_{alt,13} \\ v_{alt,14} \end{bmatrix},$$

with

$$v_{18} = \frac{\mathrm{k_{18}}\left(\mathrm{C_{cyt}}\left(\mathrm{ACA^{0,1,2,3} + ACA^4}\right) - \mathrm{C_{ext}}\left(\mathrm{ACAX^{0,1,2,3} + ACAX^4}\right)\right)}{\mathrm{Y_{vol}}}$$

$$v_{19} = \mathrm{k_0\,C_{ext}}\left(\mathrm{ACAX^{0,1,2,3} + ACAX^4}\right)$$

$$
\begin{aligned}
v_{alt,12} =& \frac{\mathrm{V_{12m}\,K_{12ACA}\,C_{cyt}\,NADH^{C}\,ACA^{4}}}{\left(\mathrm{K_{12NADH}+NADH^{C}}\right)\left(\mathrm{K_{12ACA}+ACA^{0,1,2,3}}\right)\left(\mathrm{K_{12ACA}+ACA^{0,1,2,3}+ACA^{4}}\right)}\\
v_{alt,13} =& -\frac{\mathrm{k_{13}}\left(\mathrm{C_{ext}\,EtOHX^{4}-C_{cyt}\,EtOH^{4}}\right)}{\mathrm{Y_{vol}}}\\
v_{alt,14} =& \mathrm{k_{0}\,C_{ext}\,EtOHX^{4}}
\end{aligned}
$$

Layer Dynamics:

$$
L(F^4|F^0,F^1,F^2,F^3) = \begin{bmatrix}0 & 0 & 0 & 0 & 0 & 0 & 0 & 0 & 0 & 0 & ACA^4 & EtOH^4 & EtOHX^4 & 0 & 0 & ACAX^4 & 0\end{bmatrix}^T.
$$

Fluxes to control layer:

$$
V(F^4|F^0,F^1,F^2,F^3) = \begin{bmatrix}0 & 0 & V^4\,[NAD] & V^4\,[NADH] & 0\end{bmatrix}^T.
$$

Layer $L(F^5|F^0,F^1,F^2,F^3,F^4)$

Required input trajectories:

$$
\begin{bmatrix}ACA^{0,1,2,3,4}\\ ACAX^{0,1,2,3,4}\\ CNX^{0,1,2,3,4}\end{bmatrix} = \begin{bmatrix}0&0&0&0&0&0&0&0&0&0&0&0&0&0&1&0&0&0&0&0&0&0\\0&0&0&0&0&0&0&0&0&0&0&0&0&0&0&0&0&0&0&1&0&0\\0&0&0&0&0&0&0&0&0&0&0&0&0&0&0&0&0&0&0&0&1&0\end{bmatrix} L(F^0,F^1,F^2,F^3,F^4).
$$

Required inputs from control layer:
$NADH^C$.
Ordinary differential equations:

$$
\frac{d}{dt}\begin{bmatrix}ACA^5\\ EtOH^5\\ EtOHX^5\\ ACAX^5\\ CNX^5\end{bmatrix} = \begin{bmatrix}0&0\\0&0\\0&0\\-1&0\\-1&1\end{bmatrix}\begin{bmatrix}v_{20}\\ v_{21}\end{bmatrix} + \begin{bmatrix}-1&0&0&-59&0\\1&-59&0&0&0\\0&1&-1&0&0\\0&0&0&1&-1\\0&0&0&0&0\end{bmatrix}\begin{bmatrix}v_{alt,12}\\ v_{alt,13}\\ v_{alt,14}\\ v_{alt,18}\\ v_{alt,19}\end{bmatrix}
$$

$$
\begin{bmatrix}V^5\,[NAD]\\ V^5\,[NADH]\end{bmatrix} = \begin{bmatrix}0&0\\0&0\end{bmatrix}\begin{bmatrix}v_{20}\\ v_{21}\end{bmatrix} + \begin{bmatrix}1&0&0&0&0\\-1&0&0&0&0\end{bmatrix}\begin{bmatrix}v_{alt,12}\\ v_{alt,13}\\ v_{alt,14}\\ v_{alt,18}\\ v_{alt,19}\end{bmatrix},
$$

with

$$
\begin{aligned}
v_{20} =& \mathrm{k_{20}\,C_{ext}}\left(\mathrm{ACAX^{0,1,2,3,4}+ACAX^{5}}\right)\left(\mathrm{CNX^{0,1,2,3,4}+CNX^{5}}\right)\\
v_{21} =& -\mathrm{k_{0}\,C_{ext}}\left(\mathrm{CNX^{0,1,2,3,4}-CN_{X0}+CNX^{5}}\right)\\
v_{alt,12} =& \frac{\mathrm{V_{12m}\,K_{12ACA}\,C_{cyt}\,NADH^{C}\,ACA^{5}}}{\left(\mathrm{K_{12NADH}+NADH^{C}}\right)\left(\mathrm{K_{12ACA}+ACA^{0,1,2,3,4}}\right)\left(\mathrm{K_{12ACA}+ACA^{0,1,2,3,4}+ACA^{5}}\right)}\\
v_{alt,13} =& -\frac{\mathrm{k_{13}}\left(\mathrm{C_{ext}\,EtOHX^{5}-C_{cyt}\,EtOH^{5}}\right)}{\mathrm{Y_{vol}}}\\
v_{alt,14} =& \mathrm{k_{0}\,C_{ext}\,EtOHX^{5}}\\
v_{alt,18} =& \frac{\mathrm{k_{18}}\left(\mathrm{C_{cyt}\,ACA^{5}-C_{ext}\,ACAX^{5}}\right)}{\mathrm{Y_{vol}}}\\
v_{alt,19} =& \mathrm{k_{0}\,C_{ext}\,ACAX^{5}}
\end{aligned}
$$

Layer Dynamics:

$$
L(F^5|F^0,F^1,F^2,F^3,F^4) = \begin{bmatrix}0 & 0 & 0 & 0 & 0 & 0 & 0 & 0 & 0 & 0 & ACA^5 & EtOH^5 & EtOHX^5 & 0 & 0 & ACAX^5 & CNX^5\end{bmatrix}^T.
$$

Fluxes to control layer:

$$
V(F^5|F^0,F^1,F^2,F^3,F^4) = \begin{bmatrix}0 & 0 & V^5\,[NAD] & V^5\,[NADH] & 0\end{bmatrix}^T.
$$

References

1. Chiang M, Low SH, Calderbank AR, Doyle JC (2007) Layering as optimization decomposition: A mathematical theory of network architectures. Proceedings of the IEEE 95: 255–312.

2. Prescott TP, Papachristodoulou A (2014) Signal propagation across layered biochemical networks. In: Proceedings of the American Control Conference (ACC). pp. 3399–3404.

3. Prescott TP, Papachristodoulou A (2014) Layered decomposition for the model order reduction of timescale separated biochemical reaction networks. Journal of Theoretical Biology 356: 113 - 122.

4. Lang M, Summers S, Stelling J (2014) Cutting the wires: Modularization of cellular networks for experimental design. Biophysical journal 106: 321–331.

5. Saez-Rodriguez J, Gayer S, Ginkel M, Gilles ED (2008) Automatic decomposition of kinetic models of signaling networks minimizing the retroactivity among modules. Bioinformatics 24: i213–i219.

6. Sivakumar H, Proulx SR, Hespanha JP (2014) Modular decomposition and analysis of biological networks. arXiv preprint arXiv:14120742 .

7. Del Vecchio D, Ninfa AJ, Sontag ED (2008) Modular cell biology: retroactivity and insulation. Molecular Systems Biology 4: 161.

8. Gyorgy A, Del Vecchio D (2014) Modular composition of gene transcription networks. PLoS computational biology 10: e1003486.

9. McClean MN, Mody A, Broach JR, Ramanathan S (2007) Cross-talk and decision making in MAP kinase pathways. Nature Genetics 39: 409–414.

10. Li C, Donizelli M, Rodriguez N, Dharuri H, Endler L, et al. (2010) BioModels Database: An enhanced, curated and annotated resource for published quantitative kinetic models. BMC Syst Biol 4: 92.

11. Hynne F, DanøS, Sørensen PG (2001) Full-scale model of glycolysis in Saccharomyces cerevisiae. Biophysical Chemistry 94: 121–163.
